# Design, synthesis, and cytotoxic evaluation of quinazoline-based derivatives as VEGER-2 inhibitors: comparative study against EGFR kinase activity, induction of apoptosis, and molecular docking study

**DOI:** 10.1039/d5ra03829d

**Published:** 2025-08-21

**Authors:** Reda R. Mabrouk, Arafa Musa, Maged Mohammed Saleh Al Ward, Shaimaa Hussein, Ahmed K. B. Aljohani, Mohamed Ayman El-Zahabi, Alaa Elwan

**Affiliations:** a Pharmaceutical Medicinal Chemistry& Drug Design Department, Faculty of Pharmacy (Boys), Al-Azhar University Cairo 11884 Egypt alaaelwan34@azhar.edu.eg malzahaby@azhar.edu.eg; b Directorate of Health Affairs in Buhaira-Clinical Research Department, Ministry of Health and Population Damanhour 22511 Egypt; c Department of Pharmacognosy, College of Pharmacy, Jouf University Sakaka Aljouf 72341 Saudi Arabia akmusa@ju.edu.sa; d Medicinal Chemistry Department, Faculty of Pharmacy, Al Razi University Sana'a Yemen; e Department of Pharmacology, College of Pharmacy, Jouf University Sakaka Aljouf 72341 Saudi Arabia; f Pharmacognosy and Pharmaceutical Chemistry Department, College of Pharmacy, Taibah University Medina Saudi Arabia

## Abstract

A novel series of quinazoline-based compounds were designed and synthesized as modified analogues to certain known VEGFR-2 inhibitors, as an extension of our work on the design and synthesis of new VEGFR-2 inhibitors. The anti-proliferative properties of the synthesized compounds were assessed *in vitro* against three tumor cell lines (MCF-7, HepG-2, and K-562). Compounds 8a (IC_50_ = 0.6955, 0.1871, 0.1884 μM), 8b (IC_50_ = 0.1908, 0.2242, 0.4642 μM), 8c (IC_50_ = 0.1875, 0.7344, 0.5444 μM), 8e (IC_50_ = 0.5523, 1.4357, 0.2664 μM), 9a (IC_50_ = 0.2824, 0.1871, 0.3858 μM), 9b (IC_50_ = 0.2090, 0.1944, 0.1902 μM), and 9d (IC_50_ = 0.2042, 0.3227, 2.2742 μM) showed the highest levels of the cytotoxic activity against the cell lines under investigation respectively, with IC_50_ values that were comparable to those of sorafenib (IC_50_ = 0.1283, 0.0844, 0.0606 μM). Next, the inhibitory action against VEGFR-2 kinase activity was also estimated for the synthesized compounds to confirm their mechanism of action to induce antiproliferative actions. The cytotoxicity and VEGFR-2 inhibition results were highly consistent, identifying compounds 8a (IC_50_ = 67.623, 74.864, 62.505 nM), 8b (IC_50_ = 80.740, 88.327, 78.668 nM), 9a (IC_50_ = 80.036, 85.240, 64.017 nM), 9b (IC_50_ = 19.320, 66.436, 43.052 nM), and 9d (IC_50_ = 47.042, 58.752, 80.182 nM) as top VEGFR-2 inhibitors comparing to sorafenib (IC_50_ = 87.993, 92.775, 95.735 nM). In addition, the implemented comparative study against EGFR kinase activity specifies the inhibition of VEGFR-2 kinase activity as the major mechanism correlated to the cytotoxic activity of the synthesized compounds. Furthermore, extra mechanistic studies were conducted for the synthesized compounds, including cell cycle analysis that revealed the ability of compounds 8a and 9b to arrest the HepG-2 cells at the sub-G1 phase while increasing the population of the cells to 96.3% for 8a and 94.68% for 9b in comparison to the control 68.12%. Additionally, the titled compounds caused a significant decrease in Bcl-2 expression levels, a significant increase in caspase-3, caspase-9, and BAX gene expression levels, and a suppression of TNF-α and IL-6R protein levels, indicating their significant apoptotic impact. Ultimately, the synthesized compounds' high affinity and proper binding manner inside the VEGFR-2 active site were demonstrated by molecular docking modeling.

## Abstract 2

A novel series of quinazoline-based compounds were designed and synthesized as modified analogues to certain known VEGFR-2 inhibitors, as an extension of our work on the design and synthesis of new VEGFR-2 inhibitors.

## Graphical abstract

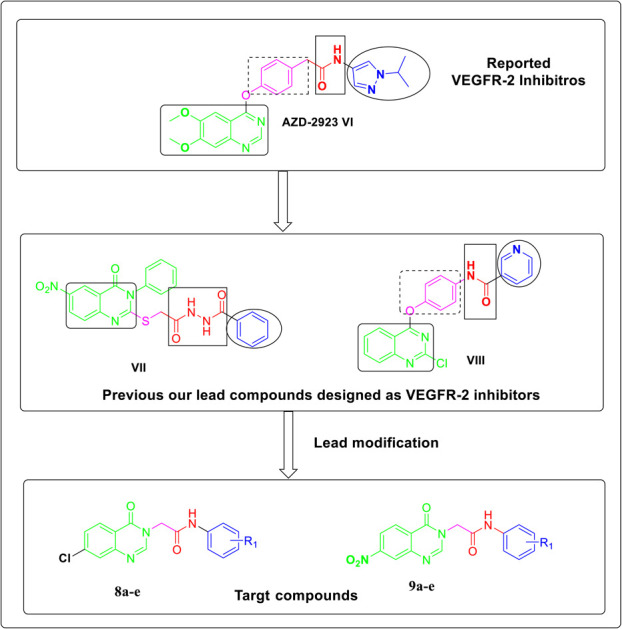

## 1. Introduction

Cancer is a disease defined by uncontrolled and excessively accelerated cell division and differentiation processes and cancerous cells have the propensity to invade or spread to other body areas through a process known as metastasis, which ultimately results in death.^1^ Cancer cells are characterized by intercellular irregularities different than normal cells.^2^ Cancerous cells must be close to blood vessels to gain access to the blood circulation system, since they require oxygen and nutrients to survive and grow uncontrollably.^3^ Angiogenesis has long been recognized as a key component of tumor growth, progression, and metastasis.^4^ Angiogenesis, the formation of new blood vessels from existing ones, is an essential process that occurs in all tumors for their progression into a clinically significant disease.^5^ Inhibiting angiogenesis is a compelling strategy for creating potent anticancer drugs to treat a wide variety of cancers.^6^ Angiogenesis is controlled by a variety of protein kinases, including growth factors.^7^ Vascular endothelial growth factor (VEGF) is a tyrosine kinase that is important for controlling angiogenesis.^8^ Its biological role includes regulating the formation of embryonic vessels and increasing vascular permeability, which in turn regulates all angiogenic processes implicated in various cancer types.^9^ VEGFs interact with three different receptors (VEGFR-1, -2, and -3) to produce their angiogenic effects.^10^ Nearly all solid tumors release VEGFR-2, the subtype that drives angiogenesis, in response to hypoxia.^4^ Increased vascular density with an additional supply of oxygen and nutrients is linked to excessive VEGFR-2 expression levels, which are then followed by the growth, metastasis, and recurrence of cancer. The VEGF/VEGFR-2 pathway is consequently a highly effective target that selectively targets malignant cells instead of healthy cells.^11^ In addition, the capacity of cancer cells to evade apoptosis is one of the main guarantees of cancer. Apoptosis, known as a process of planned cell death, is a series of metabolic responses that lead to particular cell alterations and eventual cell death.^12^ There is strong evidence that blocking VEGFR-2 can directly stop the growth of tumors by triggering apoptosis without the need for angiogenesis.^13^ From these verdicts, it is evident that one important strategy for finding new, powerful, and specific anticancer drugs is to disrupt the VEGF/VEGFR-2 signaling pathway.^14^

Over the past few years, a wide range of VEGFR-2 inhibitors have been designed; these small molecules can inhibit the VEGFR-2 pathway, resulting in diminished VEGF signal transduction in cancer cells. These include sorafenib I,^15^ sunitinib II,^16^ tivozanib III,^17^ lucitanib IV,^18^ lenvatinib V,^19^ and AZD-2923 VI.^20^

These therapeutically prescribed VEGFR-2 inhibitors were linked to detrimental side effects, including back pain, neutropenia, thrombocytopenia, left ventricular dysfunction, hand-foot syndrome, diarrhea, exhaustion, itchy skin rash, hypothyroidism, hypertension, increased alkaline phosphatase, high bilirubin, elevated AST and ALT, and osteonecrosis.^21–25^ These serious drawbacks come from the inability of traditionally used VEGFR-2 inhibitors to discriminate between cancerous and normal cell types, which is primarily linked to increased organ toxicity, a lack of cell selectivity, and a discernible propensity to cause the target cells to become resistant. Discovering harmless and effective VEGFR-2 targeted chemotherapeutic agents that prevent cancer growth is still a challenging area for medicinal chemist researchers.

For years, our research journey continued to discover new VEGFR-2 inhibitors as potent and safe anticancer agents. Our team utilized different scaffolds, including quinoxalines,^26,27^ benzoxazoles,^13,28^ thiazolidines,^29,30^ and nicotinamides^31–33^ for the synthesis of several VEGFR-2 inhibitors that showed promising anticancer activities.

Furthermore, the quinazoline nucleus represents the backbone of AZD-2932 VI (Fig. 2)^20^ and many other reported VEGFR-2 inhibitors.^34–36^ It highlights how important the quinazoline nucleus is as a flat hetero aromatic moiety in inhibitors of VEGFR-2.

In 2021,^37^ considering AZD-2932 VI, a quinazoline-based VEGFR-2 inhibitor, as the lead molecule, we developed a number of quinazoline-based derivatives that function as VEGFR-2 inhibitors. Among the synthesized derivatives, compound VII was the most potent candidate, showing an IC_50_ value of 4.6 ± 0.06 μM against VEGFR-2 kinase. Such a derivative presented strong cytotoxic activity, having IC_50_ values of 17.23, 26.10, and 30.85 μg mL^−1^ against HepG2, PC3, and MCF-7, respectively. It showed a significantly higher IC_50_ against normal WI-38 cells (145.9 μM) than the IC_50_*versus* cancer cell lines, exhibiting selectivity indices of 8.47, 5.59, and 4.73 to cell lines, respectively.

After that, in 2022, we simplified the previous derivative VII, designing a new series of quinazoline derivatives as anticancer agents that inhibit VEGFR-2 kinase. Compound VIII was the most active member, showing strong VEGFR-2 inhibition activity (IC_50_ = 60.00 Nm). This compound demonstrated IC_50_ values of 24.10, 40.90, and 33.40 μg mL^−1^ against HepG2, PC3, and MCF-7 cancer cells, respectively. It showed selectivity indices of 9.22, 5.53, and 6.92 to cell lines, respectively, in comparison to normal cell WI-38 (IC_50_ = 145.9 μM).^2^

Depending on these promising results, and in continuation of our work for the development of new VEGFR-2 inhibitors having potent and selective anti-tumor activity, we utilized the encouraging lead compounds (VII and VIII) for the design and synthesis of a new series of quinazoline-based derivatives to act as VEGFR-2 inhibitors. The developed compounds were evaluated *in vitro* for their VEGFR-2 inhibitory properties and anti-proliferative effects against definite cancer cell lines. Also, the cytotoxicity against normal cells was evaluated to ensure the safety of the synthesized compounds. In addition, the apoptotic efficacy of the target compounds has been investigated by deep biological investigations, which found the expression levels of apoptotic proteins (caspase-3, caspase-9, BAX, Bcl-2, TNF-α, and IL-6R). Finally, the synthesized derivatives were tested *in silico via* molecular docking simulation to assess their VEGFR-2 inhibitory activities.

### 1.1. Rationale of molecular design

**Fig. 1:**
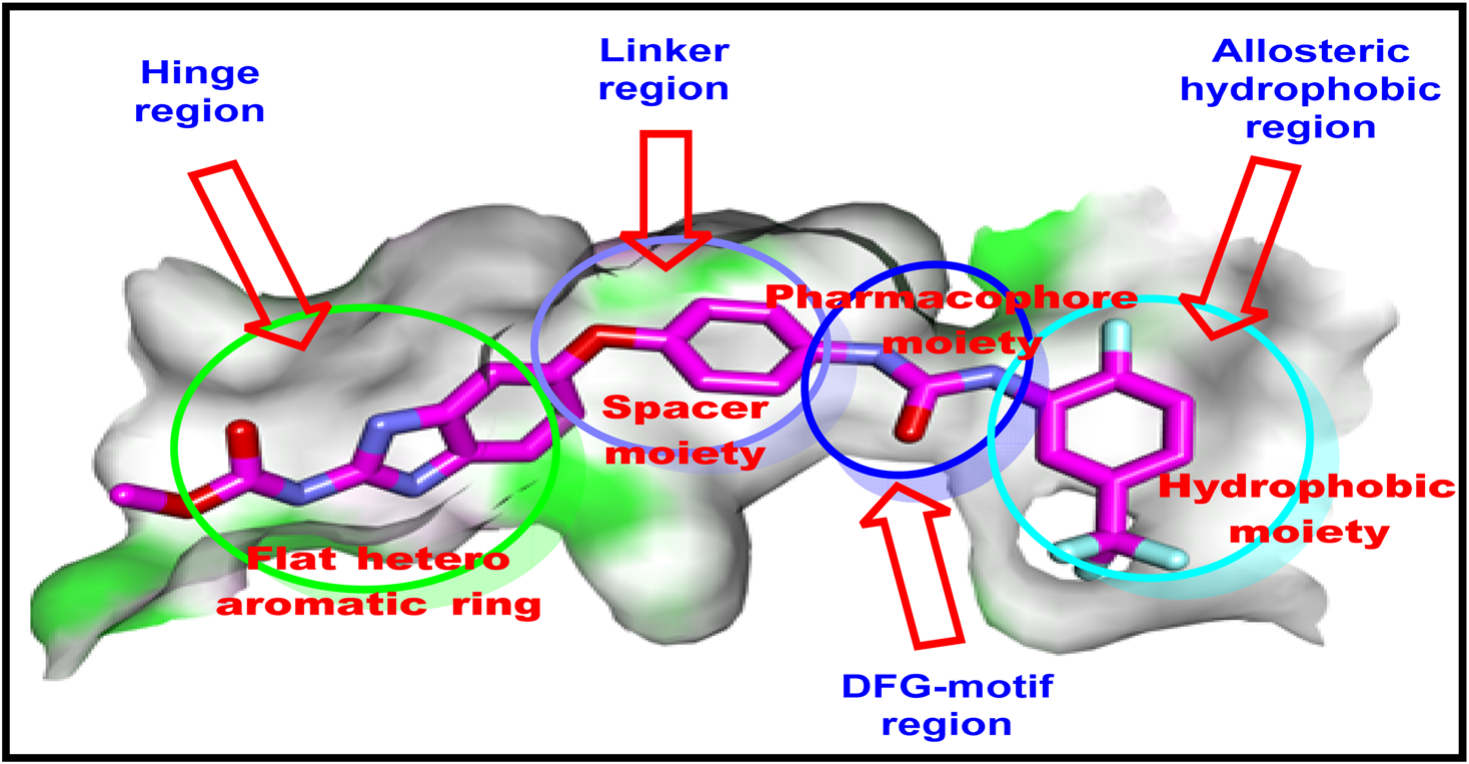
Sorafenib, as an FDA-approved anti-VEGFR-2, has the pharmacophoric features of VEGFR-2 inhibitors.

An important pharmacophoric characteristics were reported for VEGFR-2 inhibitors,^38–43^Fig. 1. These features comprise: (i) a flat hetero aromatic ring that fits at the hinge region of the ATP domain and forms an essential hydrogen bond with Cys917,^39^ (ii) linker group that spans the gap between the DFG domain and the hinge region and has a length of three to five carbon atoms,^44^ (iii) a pharmacophore moiety functions as an H-bond donor and acceptor; this modulatory group was stabilized at the DFG motif area, generating at least two hydrogen bonds with the essential amino acid (Glu883 and Asp1044),^45^ and lastly, (iv) a terminal hydrophobic moiety that forms tight hydrophobic contacts inside the allosteric hydrophobic pocket.^46^

**Fig. 2:**
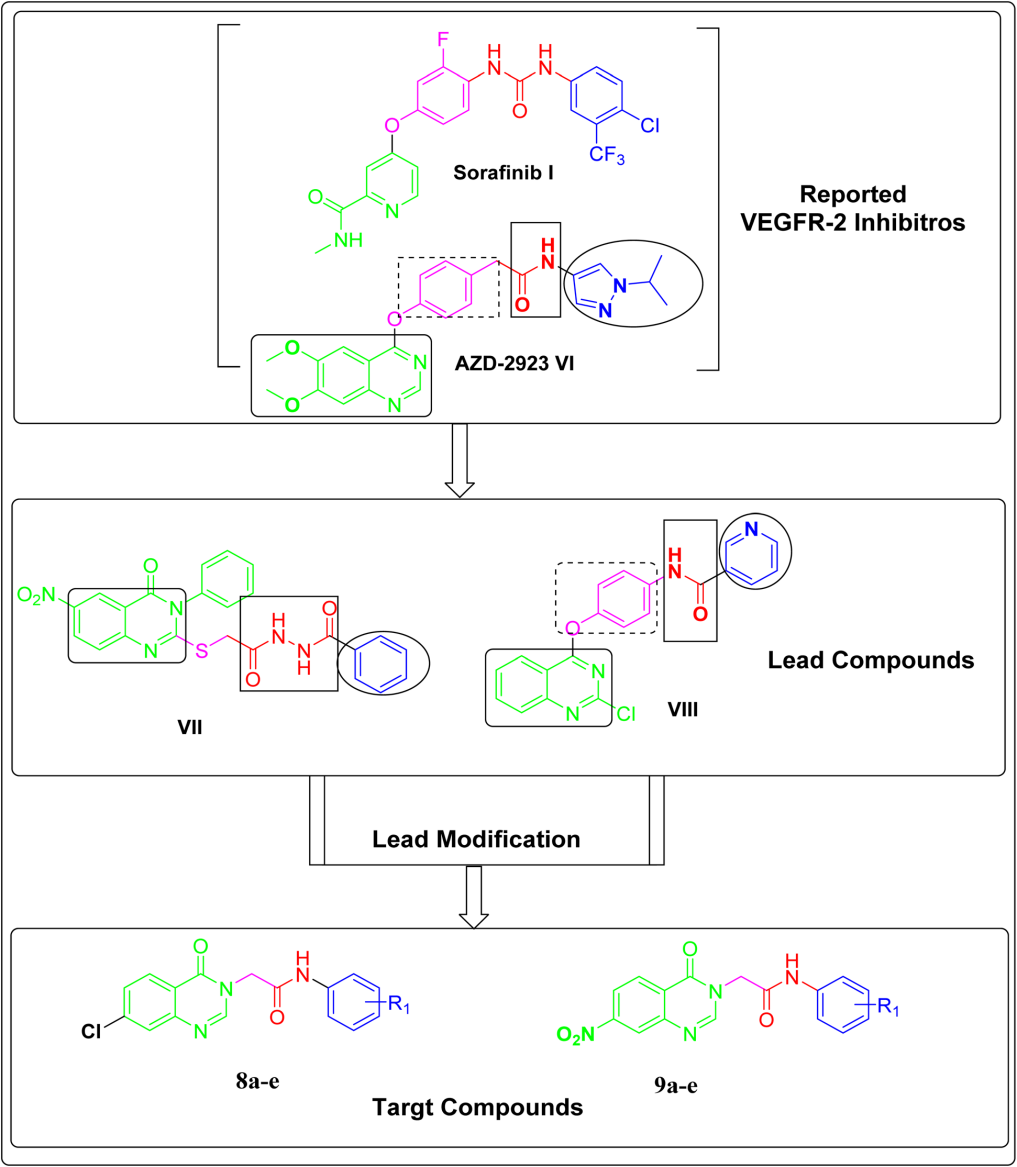
The proposed design of the target compounds.

In this current work, we modified the previous lead candidates (VII and VIII) in hopes of finding novel VEGFR-2 inhibitors. The modification of our lead compounds was carried out based on the outlined four VEGFR-2 pharmacophoric features, as follows: firstly, we maintain quinazoline-4(3*H*)-one as a heteroaromatic moiety. Utilizing the quinazoline nucleus in the strong AZD-2932 and other reported VEGFR-2 inhibitors indicates the importance and efficacy of such a nucleus as a head heteroaromatic ring. The selection of this moiety was predicated on bio-isosteric considerations. Quinazoline is a bicyclic heterocycle consisting of two fused six-membered rings, benzene, and pyrimidine, which is convenient to the large space of the ATP binding region.^47^ Due to the presence of two nitrogen atoms, it mimics the function of the pyridine ring in sorafenib that fits into the adenine binding site, facilitating H-bonding in the hinge region. In addition, we made two different substitutions (nitro and/or chloro groups) at position-7 of quinazoline-4(3*H*)-one nucleus to explore the preference of the two substituents, establishing a reliable SAR. Secondly, we selected the amide group to be the pharmacophore moiety in the designed compounds; the amide pharmacophore functioned as an H-bond donor and acceptor in AZD-2932 VI, sunitinib II, and lucitanib IV. Thirdly, to establish hydrophobic interactions with the allosteric pocket, substituted and unsubstituted phenyl rings were intended as terminal hydrophobic groups. To illustrate how both substitution and electron density affect biological activity, various substituents were selected for the terminal phenyl ring. Fig. 2.

## 2. Findings and discussion

### 2.1. Chemistry

**Scheme 1:**
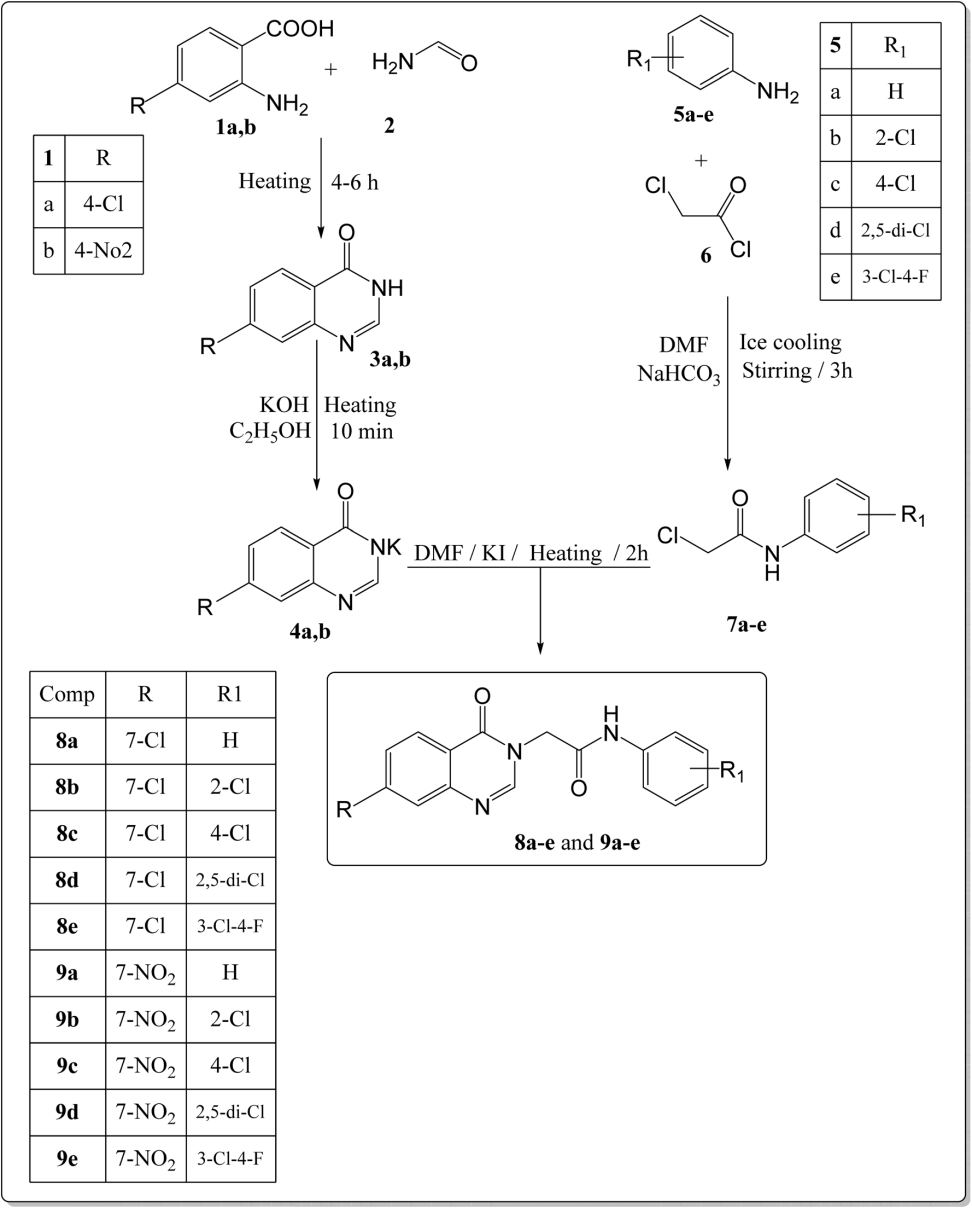
Synthetic pathways for the target compounds.

The target compounds were synthesized following to methods demonstrated in Scheme 1. Initially, 4-chloro(or 4-nitro)-2-amino-benzoic acid 1a,b was heated with formamide 2 to obtain substituted quinazoline-4(3*H*)-one 3a,b according to the reported procedure.^48^ The obtained starting materials 3a,b were subsequently treated with potassium hydroxide to get the corresponding key potassium salts 4a,b.^49^ On the other hand, following the reported procedure,^43,50^ the un/-substituted aniline derivatives 5a–e were allowed to react with the commercially available chloroacetyl chloride 6 in DMF at cold conditions to produce the corresponding 2-chloro-*N*-phenylacetamide intermediates 7a–e, respectively. Finally, the potassium salts 4a,b; each separately, were heated in DMF with the appropriate phenylacetamide intermediates 7a–e to obtain the final target compounds 8a–e and 9a–e, respectively.

Spectral data verified the structures of final compounds 8a–e and 9a–e. These compounds displayed prominent bands in their IR spectra about 3992–3200 cm^−1^, which corresponded to NHs. Furthermore, the NMR spectra supported the assigned structures of the titled compounds, revealing the presence of a singlet peak identical to NH of the amide group at a range of *δ* 10.73–10.10 ppm and a sharp singlet peak at 4.86–5.03 ppm equivalent to the aliphatic CH_2_ group. Increased integration of the aromatic protons, which corresponded to the extra phenyl ring, was also shown in NMR charts of the synthesized compounds. Moreover, ^13^C NMR spectra of these derivatives revealed the appearance of a single peak at 48.17 to 49.50 ppm corresponding to the introduced CH_2_ group of the 2-chloro-*N*-phenylacetamide moiety.

### 2.2. Biological testing

#### 2.2.1. *In vitro* anti-proliferative activities

To evaluate the anti-proliferative properties of the produced compounds, three human cancer cell lines (MCF-7, breast cancer, HepG-2, hepatocellular carcinoma, and K-562, myelogenous leukemia) were used. Based on their VEGF overexpression,^51–53^ the cell lines investigated were carefully selected. MTT assay was utilized in this test using sorafenib as a standard cytotoxic drug.^42^ The results of cytotoxic activity recorded in Table 1 revealed that all synthesized compounds were very sensitive to all three tested cell lines.

**Table 1:** *In vitro* anti-proliferative activities of the synthesized compounds against cancerous MCF-7, HepG-2, K-562, and normal HEK-293 cell lines compared to sorafenib^a^

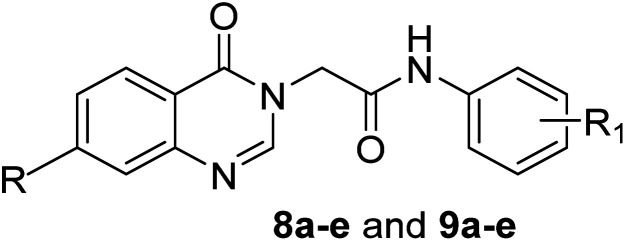						
Comp.	R	R1	Cytotoxicity against cancer cells IC_50_ (μM)			Cytotoxicity against normal cell IC_50_ (μM)
			MCF-7	HepG-2	K-562	HEK-293
8a	7-Cl	H	0.6955 ± 0.0051	0.1871 ± 0.0018	0.1884 ± 0.0017	NTb
8b	7-Cl	2-Cl	0.1908 ± 0.0040	0.2242 ± 0.0017	0.4642 ± 0.003	NTb
8c	7-Cl	4-Cl	0.1875 ± 0.0026	0.7344 ± 0.0023	0.5444 ± 0.0032	NTb
8d	7-Cl	2,5-Di-Cl	0.7462 ± 0.0024	0.6358 ± 0.0060	1.6713 ± 0.1872	NTb
8e	7-Cl	3-Cl-4-F	0.5523 ± 0.0017	1.4357 ± 0.0073	0.2664 ± 0.0017	NTb
9a	7-NO_2_	H	0.2824 ± 0.0014	0.1871 ± 0.0017	0.3858 ± 0.0032	NTb
9b	7-NO_2_	2-Cl	0.2090 ± 0.0037	0.1944 ± 0.0066	0.1902 ± 0.0037	1.7468 ± 0.0026
9c	7-NO_2_	4-Cl	0.6724 ± 0.0049	1.0001 ± 0.0049	0.5388 ± 0.0017	NTb
9d	7-NO_2_	2,5-Di-Cl	0.2042 ± 0.0026	0.3227 ± 0.0029	2.2742 ± 0.0035	NTb
9e	7-NO_2_	3-Cl-4-F	0.2314 ± 0.0023	1.5103 ± 0.0020	0.3469 ± 0.0029	NTb
Sorafenib	—	—	0.1283 ± 0.0031	0.0844 ± 0.0020	0.0606 ± 0.0026	0.1310 ± 0.0032

a NTb: not tested.

In detail, for anti-proliferative activity against MCF-7 cells, the results indicated that compounds 8b (IC_50_ = 0.1908 μM) and 8c (IC_50_ = 0.1875 μM), 9b (IC_50_ = 0.2090 μM), and 9d (IC_50_ = 0.2042 μM) were the most active members, showing strong anti-proliferative activity very close to that of sorafenib (IC_50_ = 0.1283 μM). Besides, compounds 9a (IC_50_ = 0.2824 μM) and 9e (IC_50_ = 0.2314 μM) showed moderate anti-proliferative activities compared to sorafenib. However, compounds 8a (IC_50_ = 0.6955 μM), 8d (IC_50_ = 0.7462 μM), 8e (IC_50_ = 0.5523 μM), and 9c (IC_50_ = 0.6724 μM) showed the lowest cytotoxic activities against the MCF-7 cell line.

Concerning anti-proliferative activity against HepG-2 cells, the synthesized compounds showed less sensitivity compared to MCF-7 cells. Compounds 8a (IC_50_ = 0.1871 μM), 8b (IC_50_ = 0.2242 μM), 9a (IC_50_ = 0.1871 μM), 9b (IC_50_ = 0.1944 μM), and 9d (IC_50_ = 0.3227 μM) were the most active members against the tested cell line compared to sorafenib (IC_50_ = 0.0844 μM). Compounds 8c (IC_50_ = 0.7344 μM) and 8d (IC_50_ = 0.6358 μM), on the other hand, exhibited moderate activity against the HepG-2 cell line. Compounds 8e (IC_50_ = 1.4357 μM), 9c (IC_50_ = 1.0001 μM), and 9e (IC_50_ = 1.5103 μM) appeared to be weak or inactive against the HepG-2 cell line.

With respect to anti-proliferative activity against leukemia cancer cells, K-562, the synthesized compounds showed sensitivity comparable to that of the MCF-7 cells. Particularly, compounds 8a (IC_50_ = 0.1884 μM), 8e (IC_50_ = 0.2664 μM), 9a (IC_50_ = 0.3858 μM), 9b (IC_50_ = 0.1902 μM), and 9e (IC_50_ = 0.3469 μM) were the most anti-proliferative derivatives compared to sorafenib (IC_50_ = 0.0606 μM). Moreover, compounds 8b (IC_50_ = 0.4642 μM), 8c (IC_50_ = 0.5444 μM), and 9c (IC_50_ = 0.5388 μM) displayed modest cytotoxic effects. Finally, compounds 8d (IC_50_ = 1.6713 μM) and 9d (IC_50_ = 2.2742 μM) appeared to show no significant activity against K-562 cells.

#### 2.2.2. Evaluation of cytotoxic action against normal cells

One of the main requirements for anticancer drugs is to be safe and have the least side effects on normal cells. The cytotoxic activity of the most potent anti-proliferative candidate, 9b, was tested *in vitro* against Human Embryonic Kidney 293 cells (HEK-293) in order to determine the compounds' selectivity against cancer cells as opposed to healthy ones, referring to sorafenib.^54^

The selectivity index (SI) was calculated as a ratio that measures the window between cytotoxicity (TOX) and anticancer activity (ACA) by dividing the given ACA value into the TOX value (ACA/TOX). The resulting IC_50_ value (1.7468 μM, Table 1) showed more cytotoxic activity against cancer cell lines MCF-7 (8.4-fold), HepG-2 (10-fold), and K-562 (9-fold) than against normal human kidney cells when compared to sorafenib (the corresponding IC_50_ value was 0.1310 μM, 1-, 1.5-, and 2-fold against MCF-7, HepG-2, and K-562 respectively).

#### 2.2.3. *In vitro* VEGFR-2 inhibitory assay

Among the most cytotoxic compounds (8a, 8b, 8c, 8e, 9a, 9b, 9d, and 9e), five compounds 8a, 8b, 9a, 9b, and 9d were selected to be assayed for their inhibitory activity against VEGFR-2 kinase in the examined three cell lines (MCF-7, HepG-2, and K-562). This was accomplished to validate the proposed design of the synthesized compounds and to predict the possible mechanism responsible for their induced cytotoxicity.^6^

Essentially, the results of the VEGFR-2 inhibitory assay (Table 2) greatly matched those of cytotoxicity, confirming the cytotoxic mechanism of the designed compounds. The results indicated that all the target compounds conferred excellent VEGFR-2 inhibitory activities with IC_50_ values exceeding that of the positive control sorafenib. Of all the prepared compounds, compound 9b was the most effective VEGFR-2 inhibitor; such derivative demonstrated strong VEGFR-2 inhibitory activity toward all examined cell lines MCF-7, HepG-2, and K-562 having IC_50_ values of (19.320, 66.436, 43.052 nM) respectively, that far exceeding that of sorafenib (IC_50_ = 87.993, 92.775, 95.735 nM). Compound 9d came second, displaying very strong VEGFR-2 inhibitory activity against MCF-7 and K-562 cells (IC_50_ = 47.042 and 58.752 nM, respectively) and less activity against the HepG-2 cell line (IC_50_ = 80.182 nM). Compound 8a was the third; such a compound revealed robust VEGFR-2 inhibitory activity against MCF-7, HepG-2, and K-562 with IC_50_ values equal to (67.623, 74.864, 62.505 nM), respectively. The fourth order was for compound 9a, which showed strong inhibitory action against VEGFR-2 with IC_50_ values of (80.036, 85.240, 64.017 nM) against MCF-7, HepG-2, and K-562, respectively. Finally, the last compound was 8b, which showed the least activity with IC_50s_ of 80.740, 88.327, and 78.668 nM, against MCF-7, HepG-2, and K-562, respectively.

**Table 2:** Inhibitory effects of the most cytotoxic candidates and sorafenib on VEGFR-2

Comp.	IC_50_ (nM) VEGFR-2		
	MCF-7	HepG-2	K-562
8a	67.623 ± 1.55	74.864 ± 1.22	62.505 ± 1.45
8b	80.740 ± 1.24	88.327 ± 138	78.668 ± 1.24
9a	80.036 ± 1.21	85.240 ± 1.34	64.017 ± 1.13
9b	19.320 ± 1.14	66.436 ± 1.12	43.052 ± 1.38
9d	47.042 ± 1.35	80.182 ± 1.35	58.752 ± 1.44
Sorafenib	87.993 ± 1.17	92.775 ± 1.29	95.735 ± 1.06

#### 2.2.4. Comparative study for EGFR kinase activity

It is reported that overexpression of the receptor tyrosine kinase (RTK) proteins or functional alterations result in the expression of dysregulated cell growth and cancer.^55^ Receptor tyrosine kinase (RTK) comprises subtypes, including growth factors (EGFR, VEGFR, PDGFR, FGFR, and ILGFR).^39^ Due to the deep structure similarity of receptor tyrosine kinase subtypes, the anti-cancer drug can have several targets. To identify the inhibition of VEGFR-2 kinase activity as the major mechanism of action correlated to the cytotoxic effect of the synthesized compounds, further enzyme assay was performed for the most related enzymes exhibiting kinase activity, epidermal growth factor receptor (EGFR).

EGFR, the quintessential growth factor receptor tyrosine kinase (RTK), is a crucial component in the development of numerous deadly malignancies worldwide.^56,57^ It is overexpressed in approximately 43–89% of many solid tumors, including hepatocellular, breast, colorectal, and ovarian malignancies.^58^

Assaying the inhibition activity against EGFR kinase was conducted as a comparative study to specify the mechanism and measure the sensitivity of the synthesized compounds to the VEGFR-2 enzyme rather than other kinases comprising the EGFR enzyme.

From Table 3, it is indicated that all selected compounds exhibited high IC_50_ values in comparison to erlotinib as a standard EGFR inhibitor; however, they showed low IC_50_ values very close to or may exceed that of sorafenib as a standard VEGFR inhibitor. We can infer from these findings that the prepared compounds were more sensitive to VEGFR kinase rather than EGFR kinase, so that VEGFR-2 inhibition activity may be the main possible mechanism for the cytotoxicity induced by the synthesized compounds.

**Table 3:** Comparative analysis of EGFR inhibitory activities of the most active compounds against sorafenib and erlotinib

Comp.	IC_50_ (nM) EGFR		
	MCF-7	HepG-2	K-562
8a	79.758 ± 1.07	83.210 ± 1.38	66.098 ± 1.75
8b	81.264 ± 1.53	83.722 ± 1.82	82.695 ± 1.54
9a	80.437 ± 1.59	83.703 ± 1.57	78.046 ± 1.57
9b	74.708 ± 1.76	47.738 ± 2.25	52.544 ± 2.50
9d	69.774 ± 1.36	81.572 ± 2.83	83.362 ± 1.16
Erlotinib	33.671 ± 1.34	42.927 ± 1.68	42.238 ± 1.10
Sorafenib	84.190 ± 1.40	89.177 ± 1.32	92.484 ± 1.51

#### 2.2.5. Examination of the cell cycle

In order to cause cytotoxicity, anticancer medicines must stop cell division at specific checkpoints, which are discrete stages of the cell cycle. Disturbing these phases leads to the loss of cellular functions, which significantly assured the great connection between the cell cycle and apoptosis.^59^ To explore the phase at which the synthesized compounds terminate cell proliferation, cell cycle analysis was investigated for the most active compounds 8a and 9b on HepG-2 cells.

**Fig. 3:**
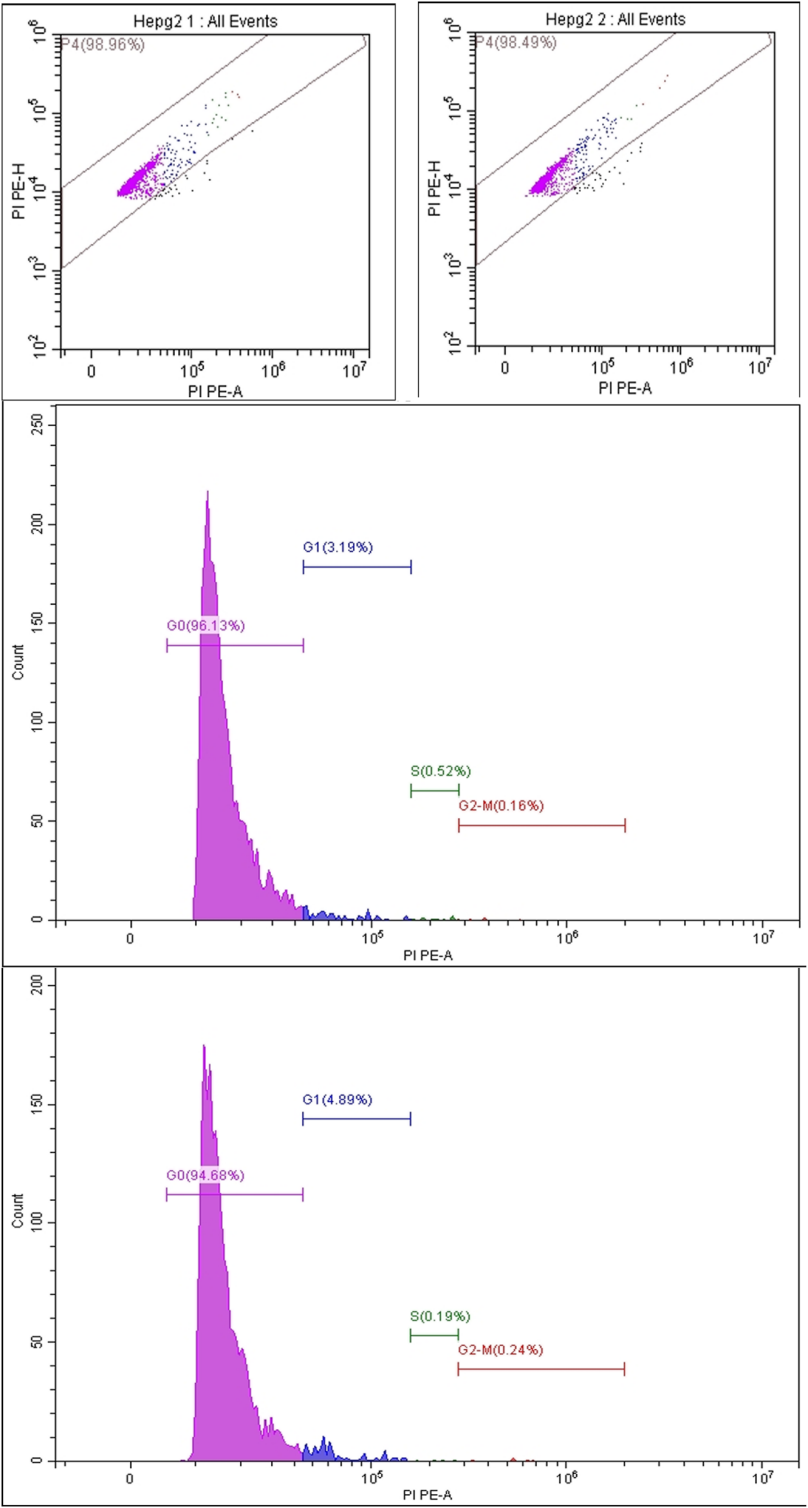
Analysis of cell cycle phases in HepG-2 cells after compounds 8a (left) and 9b (right) treatment.

From Table 4, it can be detected that compounds 8a and 9b stimulated a substantial increase in the cell population at the sub-G1 phase from 68.12% (in control cells) to 96.13% (for 8a) and 94.68% (for 9b). These compounds also caused a marked decrease in the population of HepG-2 cells at the phases G1, S, and G2/M. These results indicated that both compounds 8a and 9b were able to arrest the HepG-2 cells at the sub-G1 phase. Fig. 3.

**Table 4:** Impact of compounds 8a and 9b on HepG-2 cell cycle progression

Sample	Cell cycle phases as a percentage			
	% Sub-G1	% G1	% S	% G2/M
HepG-2 (control)	68.12	23.14	2.23	6.52
8a	96.13	3.19	0.52	0.16
9b	94.68	4.89	0.19	0.24

#### 2.2.6. Gene expression analysis for caspase-3, caspase-9, BAX, Bcl-2, TNF-a, and IL-6R

Numerous mediators regulate the induction of apoptosis in cells. Protease caspases, particularly caspase-3 and caspase-9, which are important apoptosis regulators, are among these mediators.^60^ Caspase-3 initiates extrinsic apoptosis execution, such as protease, leading to the disintegration of specific regulatory proteins required for the survival and upkeep of cells.^61^ Caspase-9 triggers apoptosis by activating other executioner caspases, including caspase-3, -6, and -7, which cleave multiple additional cellular targets.^62^ The apoptotic process is also mediated by BAX and Bcl-2, two mediators with conflicting functions. While BAX has a pro-apoptotic effect, Bcl-2 has an anti-apoptotic effect.^63,64^ Cell fate is controlled by the ratio of pro-apoptotic to anti-apoptotic proteins (BAX/Bcl-2).^6,65^ Moreover, the cytokine tumor necrosis factor-a (TNF-a) and interleukin-6 receptor (IL-6R) were reported to have pro-apoptotic effects.^66,67^ The blockade of such mediators (TNF-a & IL-6R) induced tumor cell apoptosis.^67^

All synthesized compounds were tested at their cytotoxic concentrations in HepG-2 cells for analysis of gene expression levels of apoptotic markers caspase-3, caspase-9, TNF-α, and IL-6 proteins, and only two active compounds were tested for BAX and Bcl-2 proteins.

##### 2.2.6.1. Impacts on the levels of the apoptotic markers (caspase-3 and caspase-9)

**Fig. 4:**
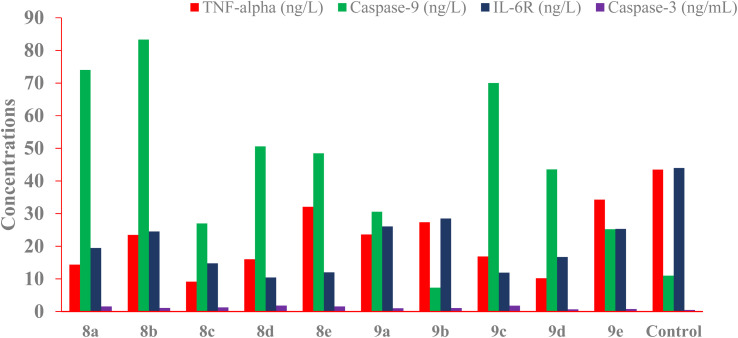
The impact of the synthesized compounds on the levels of caspase-3, caspase-9, TNF-alpha, and IL-6R gene expression in HepG-2 cells.

In comparison to control cells, the results listed in Table 5 revealed that the synthesized compounds induced a marked increase in the gene expression levels of both caspase-3 and caspase-9 (Fig. 4), indicating the significant apoptotic effect of the tested compounds. For activity toward caspase-3, compounds 8a, 8b, 8c, 8d, 8e, 9a, 9b, and 9c showed the highest expression level of the practiced protein with great folds of (3.30, 2.40, 2.72, 3.40, 3.35, 2.1, 2.35, and 3.85) respectively, while compounds 9d and 9e showed low-fold increase of (1.5, and 1.6-folds) respectively. Concerning activity against caspase-9, compounds 8a, 8b, 8c, 8d, 8e, 9a, 9c, 9d, and 9e were the most active members, causing an exceptional increase in caspase-9 expression levels with very high-folds of (6.72, 7.5, 2.5, 4.6, 4.4, 2.77, 6.37, 3.95, and 2.29) respectively. In contrast, compound 9b showed no apoptotic effect, opposing the caspase-9 protein.

**Table 5:** The effects of the synthesized compounds on levels of caspase-3, caspase-9, TNF-alpha, and IL-6R gene expression in HepG-2 cells

Sample	Caspase-3 (ng ml^−1^) ± SE	Caspase-9 (ng L^−1^) ± SE	TNF-alpha (ng L^−1^) ± SE	IL-6R (ng L^−1^) ± SE
8a	1.52 ± 0.017	74.01 ± 3.92	14.37 ± 0.45	19.54 ± 1.62
8b	1.10 ± 0.046	83.31 ± 1.28	23.47 ± 0.56	24.59 ± 0.95
8c	1.25 ± 0.090	26.98 ± 1.30	9.13 ± 0.37	14.73 ± 1.06
8d	1.82 ± 0.047	50.58 ± 4.19	16.01 ± 1.33	10.13 ± 2.40
8e	1.54 ± 0.11	48.44 ± 1.24	32.07 ± 2.32	11.93 ± 0.25
9a	0.98 ± 0.04	30.57 ± 3.39	23.6 ± 1.35	26.0 ± 0.97
9b	1.08 ± 0.028	7.31 ± 0.93	27.34 ± 1.95	28.78 ± 1.23
9c	1.77 ± 0.08	70.03 ± 0.47	16.86 ± 2.39	11.87 ± 0.80
9d	0.68 ± 0.013	43.52 ± 3.69	10.2 ± 1.53	16.64 ± 0.83
9e	0.77 ± 0.069	25.20 ± 1.27	34.27 ± 1.94	25.25 ± 0.16
Control (HepG-2)	0.460 ± 0.013	11.01 ± 5.42	43.48 ± 0.67	44.10 ± 2.57

##### 2.2.6.2. Impacts on immunomodulatory protein levels (TNF-α and IL-6R)

The examination of the Table 5 data indicated that the immunomodulatory proteins, TNF-α, and IL-6R were markedly inhibited by the synthesized compounds. Compounds 8a, 8b, 8c, 8d, 9a, 9b, 9c, and 9d were found to cause a marked inhibition of TNF-α expression levels down to (14.37%, 23.47%, 9.13%, 16.01%, 23.6%, 27.34%, 16.86%, and 10.2%) respectively comparing to the control (43.48%). Whereas compounds 8e and 9e caused mild inhibition to a percentage of (32.07% and 34.27%) respectively, Fig. 4. On the other hand, the effect on IL-6R protein was demonstrated by compounds, 8a, 8c, 8d, 8e, 9c, and 9d as these compounds offered significant inhibition of IL-6R protein to the levels of (19.54%, 14.73%, 10.13%, 11.93%, 11.87%, and 16.64%) respectively comparing the control (44.10%). Other compounds 8b, 9a, 9b, and 9c exerted mild inhibition of IL-6R protein with level values of (24.59%, 26.05%, 28.78%, and 25.25%), respectively. Fig. 4.

##### 2.2.6.3. Effects on the BAX and Bcl-2 protein levels (BAX/Bcl-2 ratio)

**Fig. 5:**
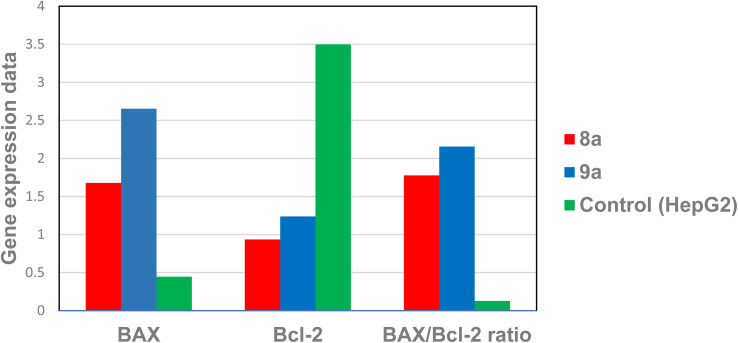
The impact of compounds 8a and 9a on HepG-2 cell expression levels of the BAX and Bcl-2 genes.

The listed data in Table 6 of studying the effect of the synthesized compounds 8a and 9a on the expression levels of the BAX and Bcl-2 proteins showed that the selected compound 9a triggered a great fold increase in BAX expression levels (6-fold) from 0.44 (control) to 2.65% in the treated cell. However, compound 8a produced a less-fold increase (3.8-fold) from 0.44 to 1.67%. In a related context, compound 9a stimulated a marked reduction in Bcl-2 expression level (2.8-fold) from 3.49 (control) to 1.23% thus BAX/Bcl-2 ratio will be elevated to 2.15% in comparison to the control 0.12% however compound 8a produced much reduction in Bcl-2 level (3.75-fold) from 3.49 (control) to 0.93 increasing the BAX/Bcl-2 ratio much less to be 1.77. Fig. 5.

**Table 6:** The effects of compounds 8a and 9a on levels of BAX and Bcl-2 gene expression in HepG-2 cells

Sample	Gene expression (fold change)		
	BAX	Bcl-2	BAX/Bcl-2 ratio
8a	1.6792 ± 0.74	0.9352 ± 0.09	1.7777 ± 0.11
9a	2.6533 ± 0.40	1.2368 ± 0.30	2.1544 ± 0.96
Control (HepG-2)	0.4448 ± 0.16	3.4983 ± 0.80	0.1260 ± 0.17

#### 2.2.7. Structure–activity relationship

The favorable outcomes of the cytotoxicity and well-matched VEGFR-2 inhibition experiment provided a useful structure–activity relationship for the synthesized compounds. The SAR of the synthesized compounds was principally built on the results of cytotoxicity and thoroughly examined in conjunction with the pharmacophoric features described in the rational design of the target compounds as VEGFR-2 inhibitors. Concerning the first feature of selecting a hetero aromatic ring to fit the hinge region, we can categorize the synthesized compounds into two scaffolds, scaffold-1 for compounds bearing 7-Cl-quinazolin-4(3*H*)-one moiety, and scaffold-2 for compounds containing 7-NO_2_-quinazolin-4(3*H*)-one moiety. The other defined features, including the linker and pharmacophore moiety (HBA + HBD), were kept unchanged for all synthesized compounds. Next, there is a reliable SAR of the synthesized compounds that can be established based on different substitutions at the hydrophobic tail moiety. So, as an assumption, the SAR of the target compounds can be instituted based on modification carried out on the hydrophobic tail in relation to substitutions of quinazoline-4(3*H*)-one moiety at 7-position with NO_2_ and/or Cl groups.

In exploring the effect of the hydrophobic tail as a key parameter affecting the activity of the synthesized compounds, it was primarily found that the activity fluctuated or was nearly more active than for the derivatives tailed with un-substituted terminal phenyl ring than those with substituted ones, this was achieved for the two scaffolds of our designed compounds, as example compounds 8a (scaffold-1) and compound 9a (scaffold-2) appeared to be more active than other derivatives. Then we studied the effect of substitution on the terminal phenyl ring; the activity depended on the nature of substituents as well as the site of the substitution as follows: Upon fixing the nature of the substituents (to be electron withdrawing group in all designed compounds) while changing the site of substitution, we can assume mainly that the activity fluctuated for mono and di-substitution with difficulty to determine the predominance of one compound over another. Then, by comparing the cytotoxicity of di-substituent derivatives as compounds 8d and 8e (scaffold-1) with that of their corresponding compounds 9d and 9e (in scaffold-2), we can conclude that 7-NO_2_-quinazolin-4(3*H*)-one moiety containing derivatives were more favorable than compounds with 7-Cl-quinazolin-4(3*H*)-one moiety. Within the di-substitutions category, the cytotoxic efficacy oscillated between similar and dissimilar substituents, which was confirmed by observing the IC_50_ values of compounds 9d and 9e against the examined cell lines. For mono substations with the same group (Cl-group, it was found that substitutions at ortho-position and para-position were with equal potency for scaffold-1(compounds 8b and 8c); however, with priority for *para*-position over *ortho*-position for scaffold-2 (9c more active than 9b).

### 2.3. Molecular docking studies

Molecular docking modeling sheds light on the drug–receptor interactions and its affinity to a specific target.^68^ Higher binding energy and a binding manner similar to the reference ligand are indicative of promising biological activity.^68^ Docking investigations of the recently synthesized derivatives were implemented to identify the orientations of these compounds and their suggested binding mode at the ATP binding site of the VEGFR-2 kinase enzyme (PDB ID: 4ASD).^14^ Sorafenib was utilized as a reference compound.

Initially, the validation procedure was carried out to confirm the docking algorithm's legitimacy. This was succeeded by re-docking the co-crystallized ligand inside the active pocket of the target protein. Achieving a low root mean square deviation (RMSD = 0.79), together with a proportionate superimposition in orientation between the native and re-docked poses, guaranteed the efficient operation of the employed protocol for the intended docking of molecules (Fig. S1 in the SI).

After the validation step, the proposed docking protocol was initially run to obtain the reported binding mode of the sorafenib.^2,69^ Such a binding pattern showed an ideal and tight interaction of sorafenib as a standard VEGFR-2 inhibitor within the active site of the VEGFR-2 (*S* score = −25.17 kcal mol^−1^). The binding mode of sorafenib presented the essential three hydrogen bonds for binding inside the VEGFR-2 active pocket. It formed one H-bond with amino acid Cys917 at the hinge region and another two with Glu883 & Asp1044 in the DFG-binding domain. In addition, it facilitated various hydrophobic interactions at the hinge region, spacer region, and terminal allosteric site, forming strong interactions with the target protein (Fig. S2 in the SI).

The newly synthesized compounds were then docked into the VEGFR-2 active pocket to estimate their binding modes and interactions in comparison to sorafenib. Table 7 summarizes the binding scores of the tested ligands, and their binding characteristics within the target protein's active region are illustrated.

**Table 7:** The binding scores of the target compounds and sorafenib against VEGFR-2 (4ASD) (computed as Δ*G* in kcal mol^−1^)

Comp.	Δ*G* [kcal mol^−1^]	Comp.	Δ*G* [kcal mol^−1^]
8a	−18.40	9a	−14.53
8b	−17.89	9b	−14.82
8c	−17.25	9c	−16.49
8d	−10.69	9d	−13.39
8e	−14.25	9e	−16.08
Sorafenib	−25.17	—	—

According to the docking studies, the proposed compounds interacted with crucial amino acids similarly to sorafenib and exhibited a high affinity toward the VEGFR-2 active site. The most cytotoxic derivatives (8a, 8b, 9a, and 9b) were chosen for examination.

**Fig. 6:**
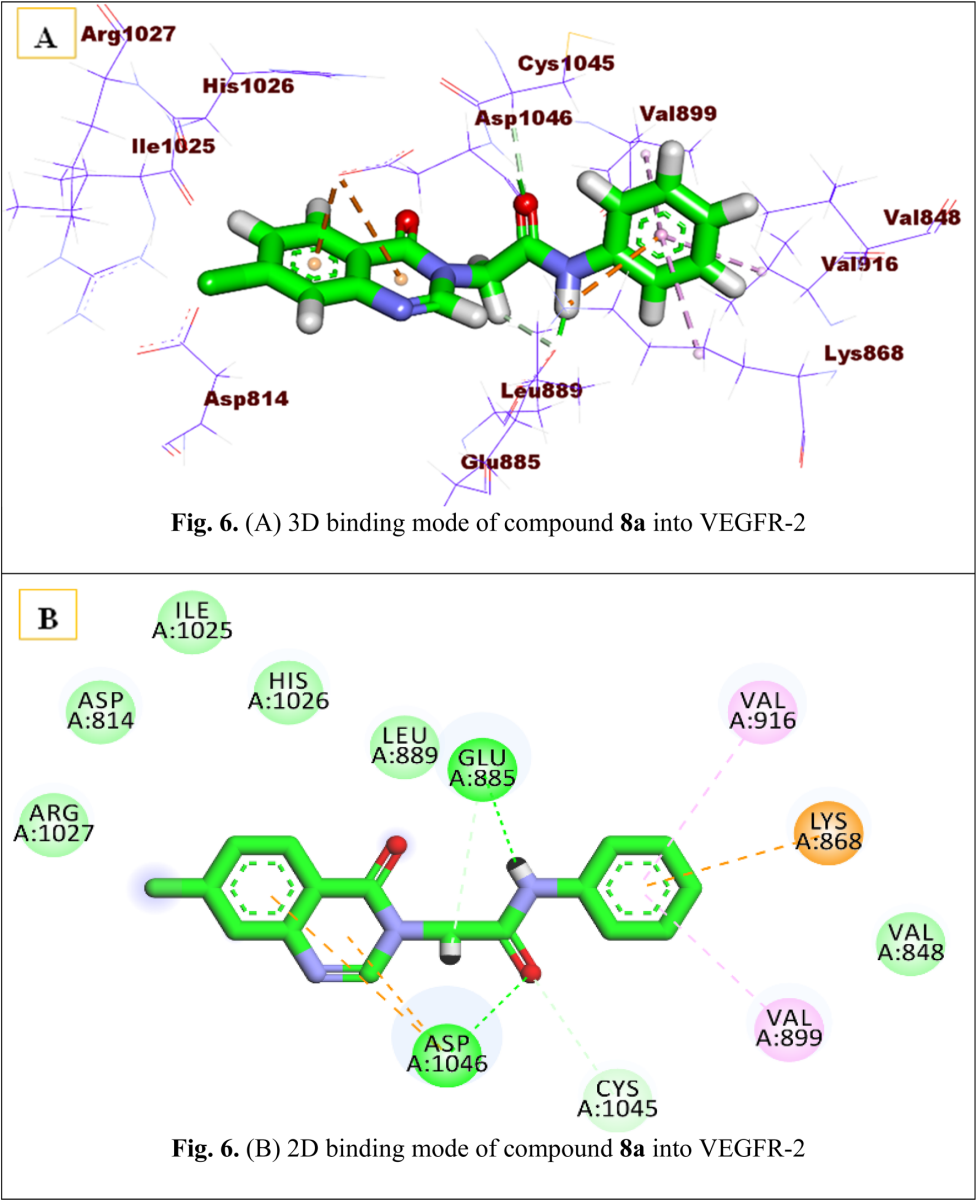
(A) 3D binding mode of compound 8a into VEGFR-2. (B) 2D binding mode of compound 8a into VEGFR-2.

Investigation of the highest-scoring pose of compound 8a revealed that it had a promising binding pattern similar to sorafenib, with an affinity value of −18.40 kcal mol^−1^. This compound interacts closely with the ATP binding domain of VEGFR-2 at the DFG region, establishing two hydrogen bonds with the essential amino acids Glu885 and Asp1046. In addition to the amide moiety achieving its required job as a pharmacophore, it also formed an extra hydrophobic interaction with Cys1045. Furthermore, the quinazoline ring fits the hinge region quite well. It stabilized at this head *via* two hydrophobic interactions with Asp1046, even though it lost interaction with the essential amino acid Cys919. Finally, the terminal phenyl ring traveled to the allosteric site, and it strongly bonded *via* hydrophobic interactions with Val916, Val899, and Lys868 Fig. 6.

**Fig. 7:**
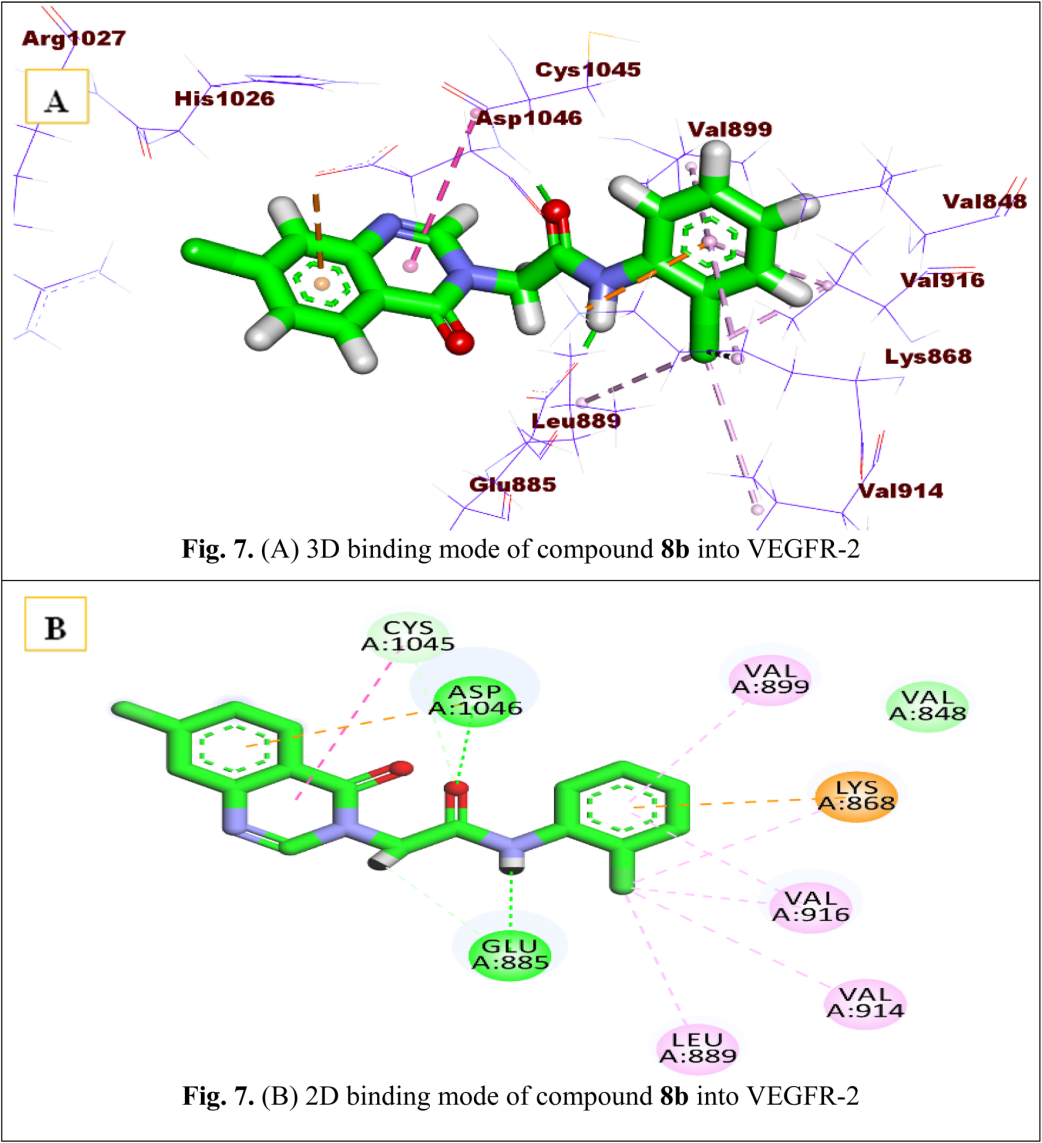
(A) 3D binding mode of compound 8b into VEGFR-2. (B) 2D binding mode of compound 8b into VEGFR-2.

Concerning the binding mode of compound 8b, such a derivative has significant docking scores of −17.89 kcal mol; it fits well into the enzyme active pocket in a way like sorafenib. The hinge area was occupied by the quinazoline moiety, which created hydrophobic contacts with Cys1045 and Asp1046 while losing hydrogen bond formation with Cys919. Additionally, the amide moiety was delivered to the DFG region, forming the two essential hydrogen bonds with Glu885 and Asp1046 amino acids. Finally, the terminal allosteric binding area was efficiently captured by the 2-chlorophenyl moiety of the designed compound, this hydrophobic tail created strong hydrophobic interactions with Val899, Ile890, Lys868, Val914, Val916, and Leu889 Fig. 7.

**Fig. 8:**
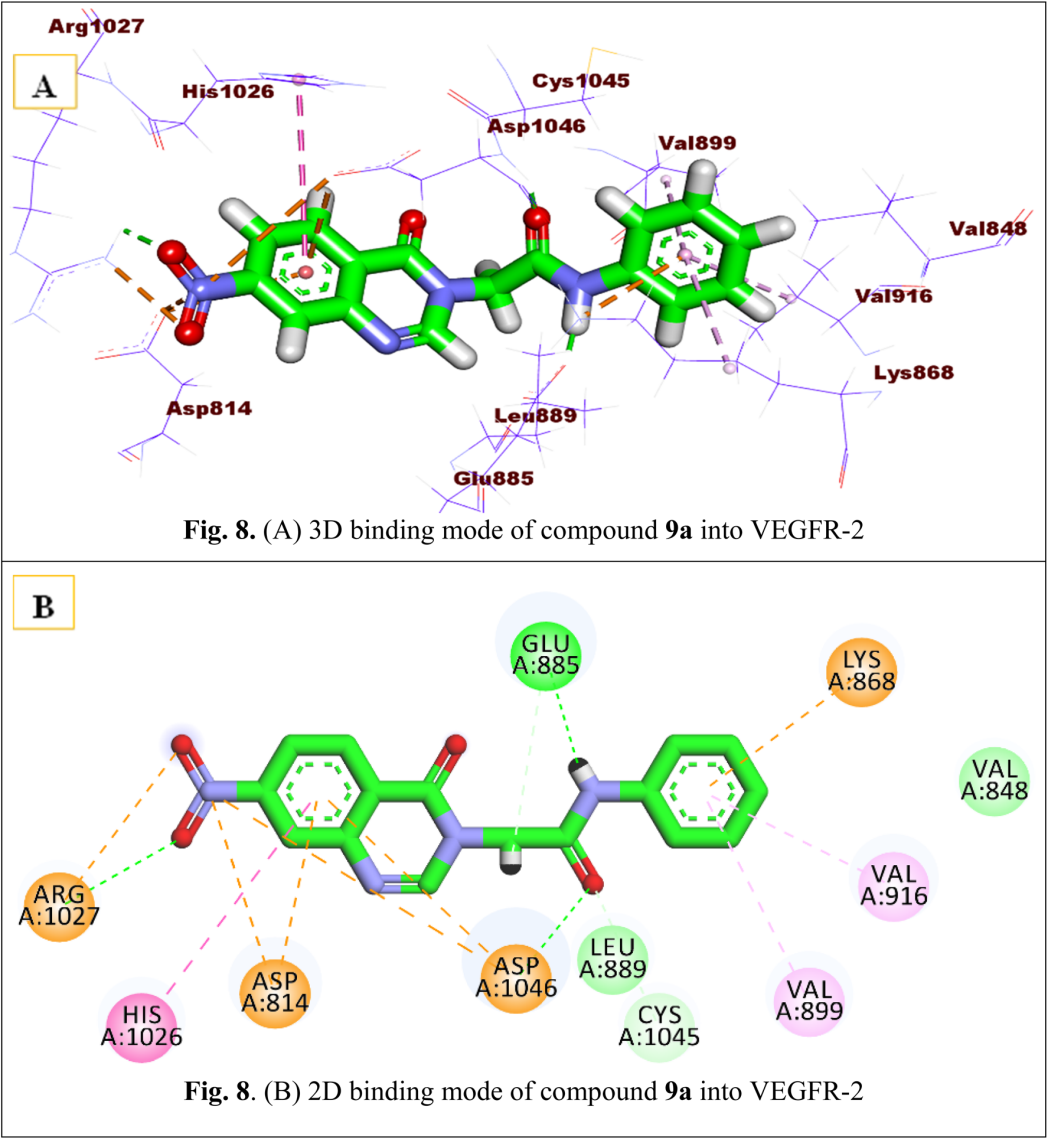
(A) 3D binding mode of compound 9a into VEGFR-2. (B) 2D binding mode of compound 9a into VEGFR-2.

Similar to the co-crystallized ligand, the proposed molecule 9a replicated the essential interactions. Such a compound could tightly bind to the receptor with a binding affinity value of −14.53 kcal mol^−1^. In the way that it is binding, the quinazoline head occupied the hinge region and was well bound through hydrophobic interaction with Asp1046, Asp814, and His1026 in addition to electrostatic attraction with Arg1027 and Asp814. Moreover, the amide group acted as an H-bond donor and acceptor and formed two hydrogen bonds, one with Glu885 and another with Asp1046. An unsubstituted phenyl ring interacted hydrophobically with Val899, lys868, and Val916 to engage the terminal allosteric hydrophobic site Fig. 8.

**Fig. 9:**
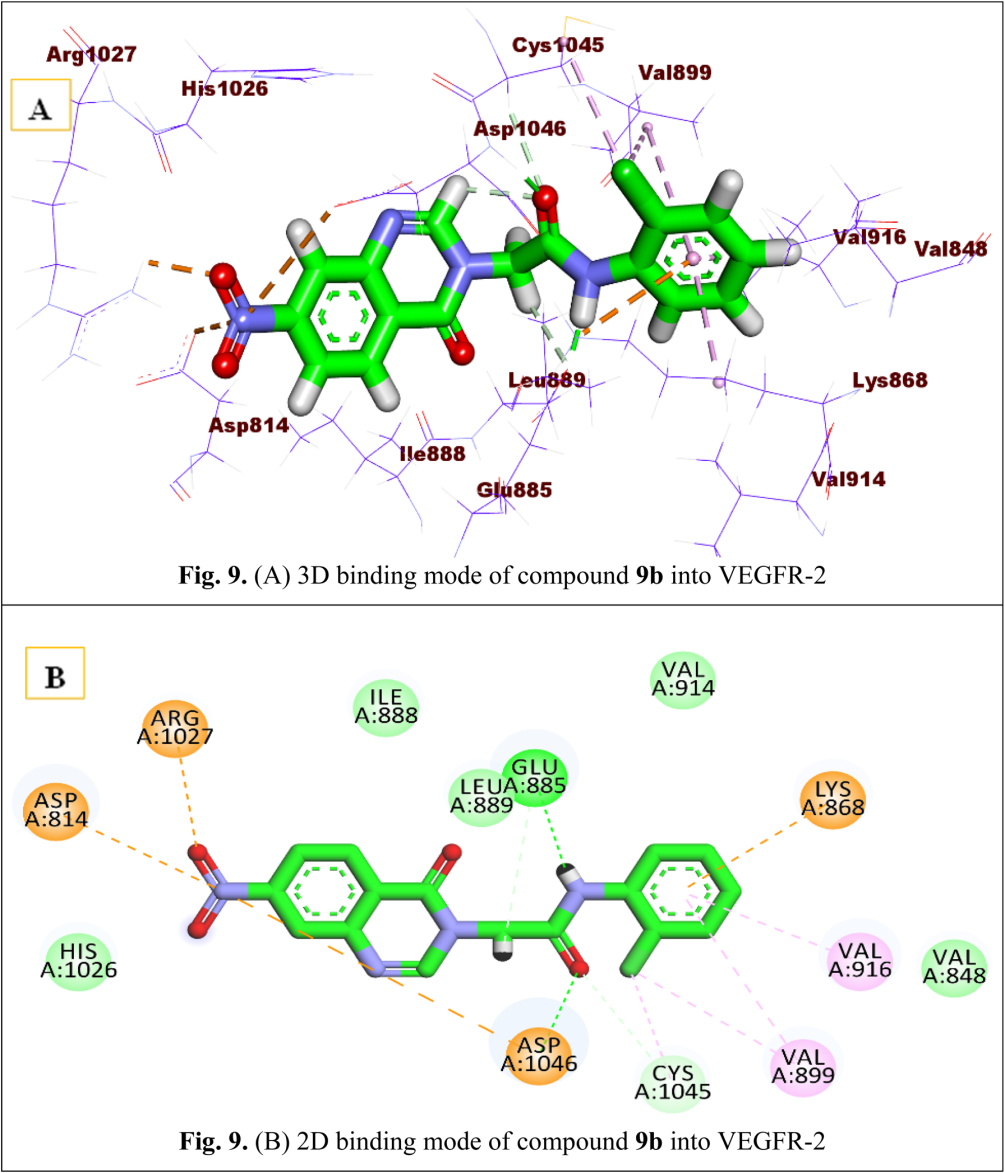
(A) 3D binding mode of compound 9b into VEGFR-2. (B) 2D binding mode of compound 9b into VEGFR-2.

Finally, the binding affinity of component 9b was −14.82 (kcal mol^−1^). It interacted with a receptor in a binding manner like that of sorafenib. The quinazoline head of this compound was attached to the hinge region and stabilized through electrostatic attraction between the oxygen atom of the NO_2_ group and Arg1027. In addition, the amide group was involved in hydrogen bonding interactions with Glu885 and Asp1046 at the DGF motif. The terminal tail, the 2-chloro phenyl ring, was well tailored in the allosteric site by the formation of several hydrophobic interactions with Val899, Lys868, Val916, and Cys1045 Fig. 9.

## 3. Conclusion

In this work, new quinazoline-based series were synthesized as modified analogues of our previously prepared VEGFR-2 inhibitors. The synthesized derivatives were designed to have the basic pharmacophoric features of the reported VEGFR-2 inhibitors and then evaluated for their anticancer activities as well as their inhibition actions against VEGFR-2 kinase. All synthesized derivatives displayed promising antiproliferative activities against three human tumor cell lines (MCF-7, HepG-2, and K-562). Particularly, compounds 8a, 8b, 8c, 8e, 9a, 9b, 9d, and 9e were the most active cytotoxic members compared to sorafenib. Correspondingly, the synthesized candidates displayed strong inhibition effects toward VEGFR-2 kinase activity; compounds 8a, 8b, 9a, 9b, and 9d were the most potent VEGFR-2 inhibitors. In addition, a definite comparative study was performed against EGFR kinase activity, which specifies the VEGFR-2 inhibition activity as the foremost mechanism for the cytotoxicities of the synthesized compounds. Furthermore, deep biological studies were conducted for the synthesized compounds to confirm their substantial apoptotic effect. This includes cell cycle analysis that demonstrated the ability of compounds 8a and 9b to arrest the HepG-2 cells with a high population at the sub-G1 phase. Also, the synthesized compounds induced a marked increase in the gene expression levels of caspase-3, caspase-9, and BAX, with a significant reduction in Bcl-2 expression level as well as marked inhibition of TNF-α and IL-6R proteins. Furthermore, the docking results assured high affinity of the synthesized compounds for VEGFR-2 kinase enzyme, exhibiting a binding mode like that of sorafenib. These biological and *in silico* results greatly supported the accessibility of the work design that represents the quinazoline-based derivatives as a chemical scaffold that may be used to develop novel VEGFR-2 inhibitors with effective anticancer properties. In the future development of this work, we must overcome the limitations of this study, which include improving the design of the target compound to be completely matched with the assigned features of the reported VEGFR-2 inhibitors. This can be achieved through maintaining the quinazoline scaffold while elongating the designed compounds by inserting a linker moiety. Also, extra biological studies concerning VEGFR-2 inhibition must be included in the future study to further confirm the approachability of the quinazoline-based derivatives to act as good VEGFR-2 inhibitors.

## 4. Experimental

### 4.1. Chemistry and materials

The solvents and equipment used in the synthesis and characterization of the target compounds are shown in the Supplementary data. Utilizing the reported methods, compounds 3a,b,^48^4a,b,^70^ and 7a–e (ref. 43) were prepared.

#### 4.1.1. General procedure for the synthesis of target compounds 8a–e and 9a–e

A proper amount of 2-chloro-*N*-phenylacetamide intermediates 7a–e, each was added to a mixture containing an equimolar amount of every potassium salt 4a and 4b separately (0.5 g, 0.0022 mol) dissolved in DMF (10 mL) with the presence of a catalytic amount of KI (0.5 g). The reaction mixture was heated for 2 h, then poured into cold water after the reaction was finished. The resulting precipitate was filtered and crystallized from ethanol to provide the corresponding final target compounds 8a–e and 9a–e, respectively.

##### 4.1.1.1. 2-(7-Chloro-4-oxoquinazolin-3(4*H*)-yl)-*N*-phenylacetamide (8a)

Pale yellow crystal (yield, 85%); m.p. = 265–268 °C; IR (KBr) *ν* cm^−1^: 3322, 3267 (NH), 3042 (CH aromatic), 2977 (CH aliphatic), 1668, 1631 (C=O), 1584 (C=N); ^1^H NMR (400 MHz, DMSO-*d*_6_) *δ* 10.44 (s, 1H, NH), 8.42 (s, 1H, N=CH–N), 8.15 (d, *J* = 8.6 Hz, 1H, CH quinaz.), 7.80 (d, *J* = 2.1 Hz, 1H, CH quinaz.), 7.59 (m, 3H, CH aromatic), 7.35–7.30 (m, 2H, CH quinaz.), 7.07 (m, *J* = 7.3, 1.2 Hz, 1H, CH aromatic), 4.87 (s, 2H, CH_2_); ^13^C NMR (101 MHz, DMSO-*d*_6_) *δ* 165.69, 160.19, 150.65, 150.41, 149.67, 139.66, 139.05, 129.42, 128.71, 127.95, 126.97, 124.17, 120.74, 119.55 (2C), 49.30; anal. calcd for C_16_H_12_ClN_3_O_2_ (313.74): C, 61.25; H, 3.86; N, 13.39; found C, 61.13; H, 4.05; N, 13.62%.

##### 4.1.1.2. 2-(7-Chloro-4-oxoquinazolin-3(4*H*)-yl)-*N*-(2-chlorophenyl)acetamide (8b)

Off-white crystal (yield, 73%); m.p. = 290–292 °C; IR (KBr) *ν* cm^−1^: 3328 (NH), 3066 (CH aromatic), 2917 (CH aliphatic), 1692, 1651 (C=O), 1598 (C=N); ^1^H NMR (400 MHz, DMSO-*d*_6_) *δ* 10.10 (s, 1H, NH), 8.45 (s, 1H, N=CH–N), 8.16 (d, *J* = 8.6 Hz, 1H, CH quinaz.), 7.79 (d, *J* = 2.1 Hz, 1H, CH quinaz.), 7.74 (dd, *J* = 8.1, 1.6 Hz, 1H, CH quinaz.), 7.61 (dd, *J* = 8.6, 2.1 Hz, 1H, CH aromatic), 7.52 (dd, *J* = 8.0, 1.5 Hz, 1H, CH aromatic), 7.33 (dd, *J* = 7.7, 1.5 Hz, 1H, CH aromatic), 7.21(dd, *J* = 7.7, 1.6 Hz, 1H, CH aromatic), 4.99 (s, 2H, CH_2_); ^13^C NMR (101 MHz, DMSO-*d*_6_) *δ* 166.44, 160.15, 150.50, 149.64, 139.64, 134.82, 130.08, 128.64, 127.97, 127.93, 127.00, 126.92, 126.60, 126.35, 120.74, 49.09; anal. calcd for C_16_H_11_Cl_2_N_3_O_2_ (348.18): C, 55.19; H, 3.18; N, 12.07; found C, 55.40; H, 3.29; N, 12.25%.

##### 4.1.1.3. 2-(7-Chloro-4-oxoquinazolin-3(4*H*)-yl)-*N*-(4-chlorophenyl)acetamide (8c)

Yellow crystal (yield, 82%); m.p. = 280–282 °C; IR (KBr) *ν* cm^−1^: 3310 (NH), 3077 (CH aromatic), 2959 (CH aliphatic), 1651 (C=O), 1599 (C=N); ^1^H NMR (400 MHz, DMSO-*d*_6_) *δ* 10.59 (s, 1H, NH), 8.42 (s, 1H, N=CH–N), 8.14 (d, *J* = 8.5 Hz, 1H, CH quinaz.), 7.80 (d, *J* = 2.0 Hz, 1H, CH quinaz.), 7.63–7.59 (m, 3H, CH aromatic & CH quinaz.), 7.40–7.36 (m, 2H, CH aromatic), 4.86 (s, 2H, CH_2_); ^13^C NMR (101 MHz, DMSO-*d*_6_) *δ* 165.40, 159.69, 149.98, 149.17, 139.20, 137.49, 128.81 (2C) 128.14, 127.49, 127.18, 126.46, 120.65 (2C), 120.23, 48.84; anal. calcd for C_16_H_11_Cl_2_N_3_O_2_ (348.18): C, 55.19; H, 3.18; N, 12.07; found C, 55.37; H, 3.40; N, 12.31%.

##### 4.1.1.4. 2-(7-Chloro-4-oxoquinazolin-3(4*H*)-yl)-*N*-(2,6-dichlorophenyl)acetamide (8d)

White crystal (yield, 87%); m.p. = 260–263 °C; IR (KBr) *ν* cm^−1^: 3313, 3200 (NH), 3082 (CH aromatic), 2947 (CH aliphatic), 1690, 1670 (C=O), 1602 (C=N); ^1^H NMR (400 MHz, DMSO-*d*_6_) *δ* 10.39 (s, 1H, NH), 8.44 (s, 1H, N=CH–N), 8.17 (d, *J* = 8.5 Hz, 1H, CH quinaz.), 7.79 (d, *J* = 2.1 Hz, 1H, CH quinaz.), 7.62–7.53 (m, 3H, CH aromatic & CH quinaz.), 7.39–7.33 (m, 1H, CH aromatic), 4.96 (s, 2H, CH_2_); ^13^C NMR (101 MHz, DMSO-*d*_6_) *δ* 166.09, 160.01, 150.41, 150.32, 149.61, 139.62, 133.95 (2C), 132.74, 129.89, 129.05 (2C), 127.93, 126.92, 120.78, 48.39; anal. aalcd for C_16_H_10_Cl_3_N_3_O_2_ (382.63): C, 50.23; H, 2.63; N, 10.98; found C, 50.41; H, 2.89; N, 11.20%.

##### 4.1.1.5. *N*-(3-Chloro-4-fluorophenyl)-2-(7-chloro-4-oxoquinazolin-3(4*H*)-yl)acetamide (8e)

Pale yellow crystal (yield, 85%); m.p. = 175–177 °C; IR (KBr) *ν* cm^−1^: 3353, 3322 (NH), 3067 (CH aromatic), 2954 (CH aliphatic), 1692, 1674 (C=O), 1600 (C=N); ^1^H NMR (400 MHz, DMSO-*d*_6_) *δ* 10.69 (s, 1H, NH), 8.43 (s, 1H, N=CH–N), 8.15 (d, *J* = 8.6 Hz, 1H, CH quinaz.), 7.89 (dd, *J* = 6.8, 2.5 Hz, 1H, CH quinaz.), 7.80 (d, *J* = 2.0 Hz, 1H, CH quinaz.), 7.61 (dd, *J* = 8.6, 2.1 Hz, 1H, CH aromatic), 7.48–7.38 (m, 1H, CH aromatic), 7.40 (m, *J* = 9.1 Hz, 1H, CH aromatic), 4.87 (s, 2H, CH_2_); ^13^C NMR (101 MHz, DMSO-*d*_6_) *δ* 166.07, 160.18, 150.41, 149.63, 139.70, 136.25, 128.61, 127.99, 126.95, 121.02, 120.69, 119.93, 119.86, 117.73, 117.52, 49.31; anal. calcd for C_16_H_10_Cl_2_FN_3_O_2_ (366.17): C, 52.48; H, 2.75; N, 11.48; found C, 52.67; H, 2.98; N, 11.75%.

##### 4.1.1.6. 2-(7-Nitro-4-oxoquinazolin-3(4*H*)-yl)-*N*-phenylacetamide (9a)

Off-white crystal (yield, 74%); m.p. = 245–248 °C; IR (KBr) *ν* cm^−1^: 3459 (NH), 3061 (CH aromatic), 2977 (CH aliphatic), 1701, 1664 (C=O), 1619 (C=N); ^1^H NMR (400 MHz, DMSO-*d*_6_) *δ* 10.48 (s, 1H, NH), 8.56 (s, 1H, N=CH–N), 8.45 (d, *J* = 2.2 Hz, 1H, CH quinaz.), 8.39 (d, *J* = 8.7 Hz, 1H, CH quinaz.), 8.30 (dd, *J* = 8.8, 2.3 Hz, 1H, CH quinaz.), 7.59 (d, *J* = 8.0 Hz, 2H, CH aromatic), 7.33 (m, *J* = 7.7 Hz, 2H, CH aromatic), 7.08 (m, *J* = 7.4 Hz, 1H, CH aromatic), 4.93 (s, 2H, CH_2_); ^13^C NMR (101 MHz, DMSO-*d*_6_) *δ* 165.45, 159.86, 151.70, 151.13, 148.91, 139.00, 129.43, 129.38, 129.32, 126.10, 122.94, 122.76, 121.24, 119.58 (2C), 49.50; anal. calcd for C_16_H_12_N_4_O_4_ (324.30): C, 59.26; H, 3.73; N, 17.28; found C, 59.37; H, 3.96; N, 17.46%.

##### 4.1.1.7. *N*-(2-Chlorophenyl)-2-(7-nitro-4-oxoquinazolin-3(4*H*)-yl)acetamide (9b)

Greenish white crystal (yield, 65%); m.p. = 265–266 °C; IR (KBr) *ν* cm^−1^: 3992 (CH aromatic), 2892 (CH aliphatic), 1696, 1658 (C=O), 1586 (C=N); ^1^H NMR (400 MHz, DMSO-*d*_6_) *δ* 10.13 (s, 1H, NH), 8.57 (s, 1H, N=CH–N), 8.50–8.35 (m, 2H, CH aromatic), 8.30 (dd, *J* = 8.8, 2.3 Hz, 1H, CH quinaz.), 7.73 (d, *J* = 8.1 Hz, 1H, CH quinaz.), 7.53 (d, *J* = 8.0 Hz, 1H, CH quinaz.), 7.34 (m, *J* = 7.6 Hz, 1H, CH aromatic), 7.22 (m, *J* = 7.8 Hz, 1H, CH aromatic), 5.03 (s, 2H, CH_2_); ^13^C NMR (101 MHz, DMSO-*d*_6_) *δ* 166.21, 159.83, 151.69, 151.22, 148.89, 134.77, 130.10, 128.92, 127.99, 127.07, 126.64, 126.39, 126.12, 122.85, 121.35, 49.31; anal. calcd for C_16_H_11_ClN_4_O_4_ (358.74): C, 53.57; H, 3.09; N, 15.62; found C, 53.81; H, 3.26; N, 15.84%.

##### 4.1.1.8. *N*-(4-Chlorophenyl)-2-(7-nitro-4-oxoquinazolin-3(4*H*)-yl)acetamide (9c)

Pale yellow crystal (yield, 95%); m.p. = 255–258 °C; IR (KBr) *ν* cm^−1^: 3302 (NH), 3069 (CH aromatic), 2981(CH aliphatic), 1643 (C=O), 1599 (C=N); ^1^H NMR (400 MHz, DMSO-*d*_6_) *δ* 10.63 (s, 1H, NH), 8.55 (s, 1H, N=CH–N), 8.46 (d, *J* = 2.3 Hz, 1H, CH quinaz.), 8.39 (d, *J* = 8.8 Hz, 1H, CH quinaz.), 8.30 (dd, *J* = 8.7, 2.3 Hz, 1H, CH quinaz.), 7.65–7.58 (m, 2H, CH aromatic), 7.41–7.38 (m, 2H, CH aromatic), 4.92 (s, 2H, CH_2_); ^13^C NMR (101 MHz, DMSO-*d*_6_) *δ* 165.65, 159.86, 151.72, 151.18, 148.90, 137.93, 129.31 (2C), 128.91, 127.72, 126.08, 122.87, 121.39, 121.15 (2C), 49.54; anal. calcd for C_16_H_11_ClN_4_O_4_ (358.74): C, 53.57; H, 3.09; N, 15.62; found C, 53.71; H, 3.32; N, 15.89%.

##### 4.1.1.9. *N*-(2,6-Dichlorophenyl)-2-(7-nitro-4-oxoquinazolin-3(4*H*)-yl)acetamide (9d)

Off-white crystal (yield, 96%); m.p. = 285–287 °C; IR (KBr) *ν* cm^−1^: 3364, 3290 (NH), 3086 (CH aromatic), 2962 (CH aliphatic), 1684, 1643 (C=O), 1599 (C=N); ^1^H NMR (400 MHz, DMSO-*d*_6_) *δ* 10.43 (s, 1H, NH), 8.39 (m, 3H, N=CH–N & CH quinaz.), 7.45 (m, 4H, CH quinaz. & CH aromatic), 5.00 (s, 2H, CH_2_); ^13^C NMR (101 MHz, DMSO-*d*_6_) *δ* 165.39, 159.18, 151.15, 150.61, 148.36, 133.46 (2C), 132.21, 129.41, 128.56 (2C), 128.45, 125.68, 122.32, 120.81, 48.17; anal. calcd for C_16_H_10_Cl_2_N_4_O_4_ (393.18): C, 48.88; H, 2.56; N, 14.25; found C, 49.09; H, 2.70; N, 14.51%.

##### 4.1.1.10. *N*-(3-Chloro-4-fluorophenyl)-2-(7-nitro-4-oxoquinazolin-3(4*H*)-yl)acetamide (9e)

White crystal (yield, 85%); m.p. > 300 °C; IR (KBr) *ν* cm^−1^:, 3074 (CH aromatic), 2923 (CH aliphatic), 1626 (C=O), 1603 (C=N); ^1^H NMR (400 MHz, DMSO-*d*_6_) *δ* 10.73 (s, 1H, NH), 8.60–8.23 (m, 4H, N=CH–N & CH quinaz.), 7.81–7.66 (m, 1H, CH aromatic), 7.46–7.25 (m, 2H, CH aromatic), 4.91 (s, 2H, CH_2_); ^13^C NMR (101 MHz, DMSO-*d*_6_) *δ* 165.33, 159.37, 151.24, 150.65, 148.41, 135.46, 128.41, 125.57, 122.38, 120.91, 117.77, 117.59, 115.46, 108.30, 108.09, 49.01; anal. calcd for C_16_H_10_ClFN_4_O_4_ (376.73): C, 51.01; H, 2.68; N, 14.87; found C, 51.27; H, 2.89; N, 15.06%.

### 4.2. Biological testing

#### 4.2.1. *In vitro* anti-proliferative activity

Anti-proliferative activities of the synthesized compounds were assessed against cancer cell lines (MCF-7, breast cancer, HepG-2, hepatocellular carcinoma, and K-562, myelogenous leukemia), and normal cell line (HEK-293, Human Embryonic Kidney 293 cells) using the MTT assay protocol^71,72^ as described in the SI.

#### 4.2.2. *In vitro* VEGFR-2 and EGFR kinases assay

An ELISA kit was used to test the VEGFR-2 and EGFR inhibitory activities of the most cytotoxic compounds in accordance with the reported technique^73^ as detailed in the SI.

#### 4.2.3. Analysis of cell cycle

Propidium iodide (PI) staining and flow cytometry analysis^74,75^ were used to analyze the cell cycle for derivatives 8a and 9a, as illustrated in the SI.

#### 4.2.4. Gene expression analysis for caspase-3, caspase-9, BAX, Bcl-2, TNF-a, and IL-6R

The effect of the synthesized compounds on the expression of cleaved caspase-3, caspase-9, BAX, Bcl-2, TNF-α, and IL-6R proteins was determined using qRT-PCR^76–78^ as designated in the SI.

### 4.3. Molecular docking studies

The docking studies were performed against the crystal structure of VEGFR-2 [PDB ID: 4ASD] utilizing MOE.14 software^31,33,79^ as described in the Supplementary data. The final figures were visualized using Discovery Studio 4.0.^80^

## Conflicts of interest

There is no conflict of interest.

## Supplementary Material

RA-015-D5RA03829D-s001

## Data Availability

Supplementary data related to this manuscript are found in a separate file. See DOI: https://doi.org/10.1039/d5ra03829d.
