# Supplementary material for: Design, synthesis, and cytotoxic evaluation of quinazoline-based derivatives as VEGER-2 inhibitors: comparative study against EGFR kinase activity, induction of apoptosis, and molecular docking study
Source: RSC Adv. 2025 Aug 21;15(36):29593–612. doi: 10.1039/d5ra03829d (PMC12377202; doi:10.1039/d5ra03829d)
Supplement: RA-015-D5RA03829D-s001 [file RA-015-D5RA03829D-s001.pdf]

Supplementary Information (SI) for RSC Advances.
This journal is © The Royal Society of Chemistry 2025

Design, synthesis, and cytotoxic evaluation of quinazoline-based derivatives as VEGER-2 inhibitors; comparative study against EGFR kinase activity, induction of apoptosis, and molecular docking study

Reda R. Mabrouk[1,2], Arafa Musa[3*], Maged Mohammed Saleh Al Ward[1,4], Shaimaa Hussein[5], Ahmad K. B. Aljohani[6], Mohamed Ayman El-Zahabi[1*], and Alaa Elwan[1*]

[1]Pharmaceutical Medicinal Chemistry& Drug Design Department, Faculty of Pharmacy (Boys), Al-Azhar University, Cairo 11884, Egypt.

[2]Directorate of Health Affairs in Buhaira-Clinical Research Department, Ministry of Health and Population, Damanhour 22511, Egypt.

[3]Department of Pharmacognosy, College of Pharmacy, Jouf University, Sakaka, Aljouf 72341, Saudi Arabia.

[4]Medicinal Chemistry Department, Faculty of Pharmacy, Al Razi University, Sana'a, Yemen

[5]Department of Pharmacology, College of Pharmacy, Jouf University, Sakaka, Aljouf 72341, Saudi Arabia.

[6]Pharmacognosy and Pharmaceutical Chemistry Department, College of Pharmacy, Taibah University, Medina, Saudi Arabia.

*** Corresponding authors:**

Alaa Elwan

Pharmaceutical Medicinal Chemistry& Drug Design Department, Faculty of Pharmacy (Boys), Al-Azhar University, Cairo 11884, Egypt.

Email: alaaelwan34@azhar.edu.eg

Mohamed Ayman ElZahabi

Pharmaceutical Medicinal Chemistry& Drug Design Department, Faculty of Pharmacy (Boys), Al-Azhar University, Cairo 11884, Egypt.

Email: malzahaby@azhar.edu.eg

Arafa Musa

[3]Department of Pharmacognosy, College of Pharmacy, Jouf University, Sakaka, Aljouf 72341, Saudi Arabia

Email: akmusa@ju.edu.sa

Content

1	**4.1. Chemistry and materials**
2	**Spectral data of final target compounds 8a-e and 9a-e**
3	***In vitro* studies**<br>**4.2. Experimental of Biological testing**<br>**4.2.1**. *In vitro* anti-proliferative activity<br>**4.2.2.** *In vitro* VEGFR-2 and EGFR kinases assay<br>**4.2.3.** Flow cytometry analysis for cell cycle<br>**4.2.4.** Quantitative Real Time Reverse-Transcriptase PCR technique analysis for expression of expression levels of Caspase-3, Caspase-9, BAX, Bcl-2, TNF-a, and IL-6R
4	Raw data of biological testing, cytotoxicity and VEGFR-2 and EGFR inhibition assay
5	***In silico* studies**<br>**4.3.** Experimental of molecular docking studies<br>-Fig. S1; illustrating validation step<br>-Fig. S2; illustrating binding mode of sorafenib to the active site of VEGFR-2

1- **4.1. Chemistry and material**

All melting points were carried out by open capillary method on a Gallen kamp Melting point apparatus. The infrared spectra were recorded on pye Unicam SP 1000 IR spectrophotometer using potassium bromide disc technique. Proton magnetic resonance 1HNMR spectra were recorded on a Bruker 400 Megahertz-nuclear magnetic resonance (400 MHZ-NMR) spectrophotometer. Carbon-13 (C13) nuclear magnetic resonance (13CNMR) spectra were recorded on a Bruker 100 Megahertz-nuclear magnetic resonance (101 MHZ-NMR) spectrophotometer. Elemental analyses (C, H, N) were performed on a CHN analyzer at Regional Center for Mycology and Biotechnology, Al-Azhar University. Tetramethylsilane (TMS) was used as internal standard and chemical shifts were measured in δ scale one part per million (ppm). All compounds were within ±0.4 of the theoretical values. The reactions were monitored by thin-layer chromatography (TLC) using TLC sheets precoated with UV fluorescent silica gel Merck 60 F254 plates and were visualized using ultraviolet (UV) lamp and different solvents as mobile phases.

2- **Spectral data of final target compounds 8a-e and 9a-e**

IR of compound 8a

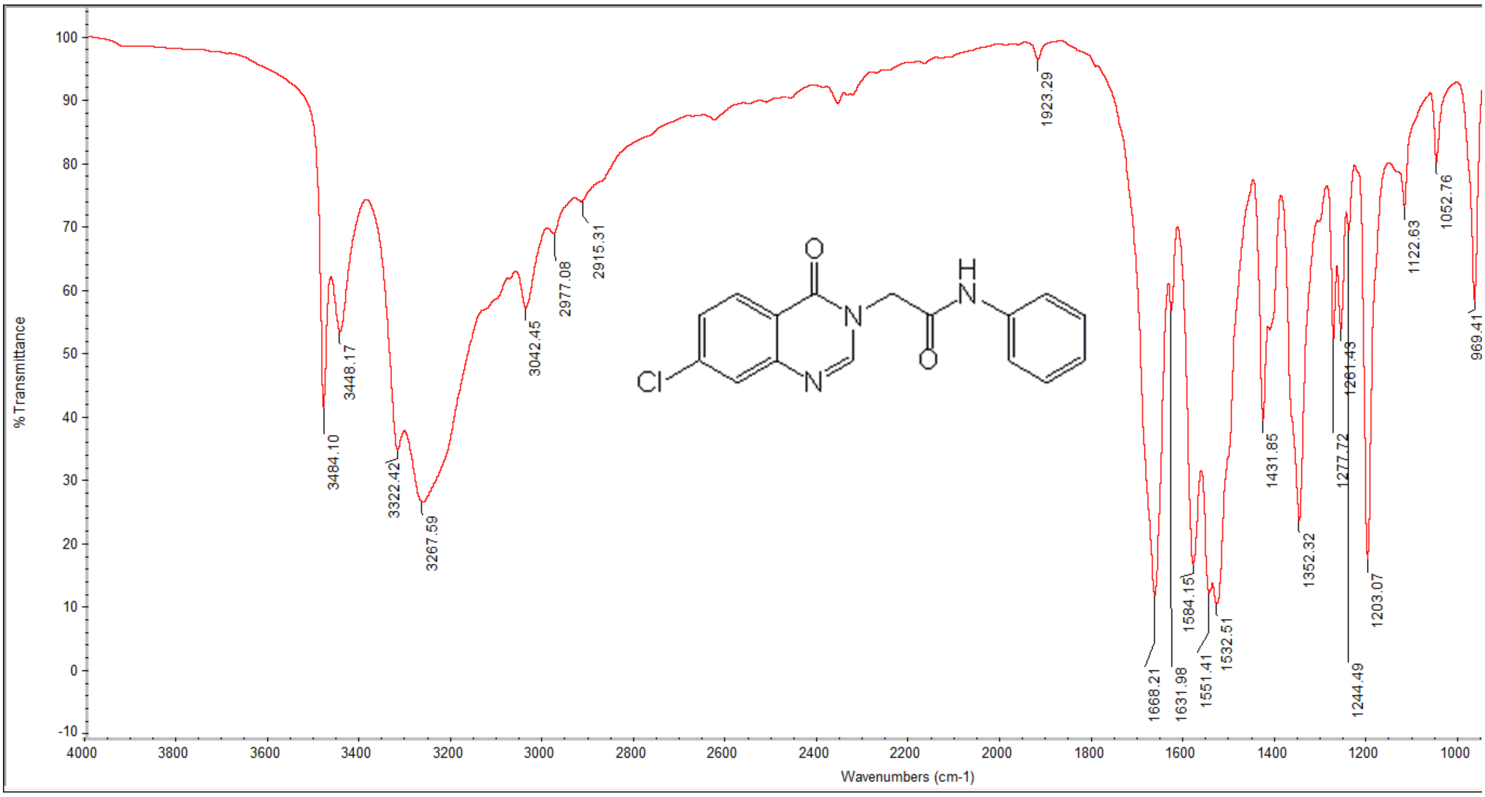

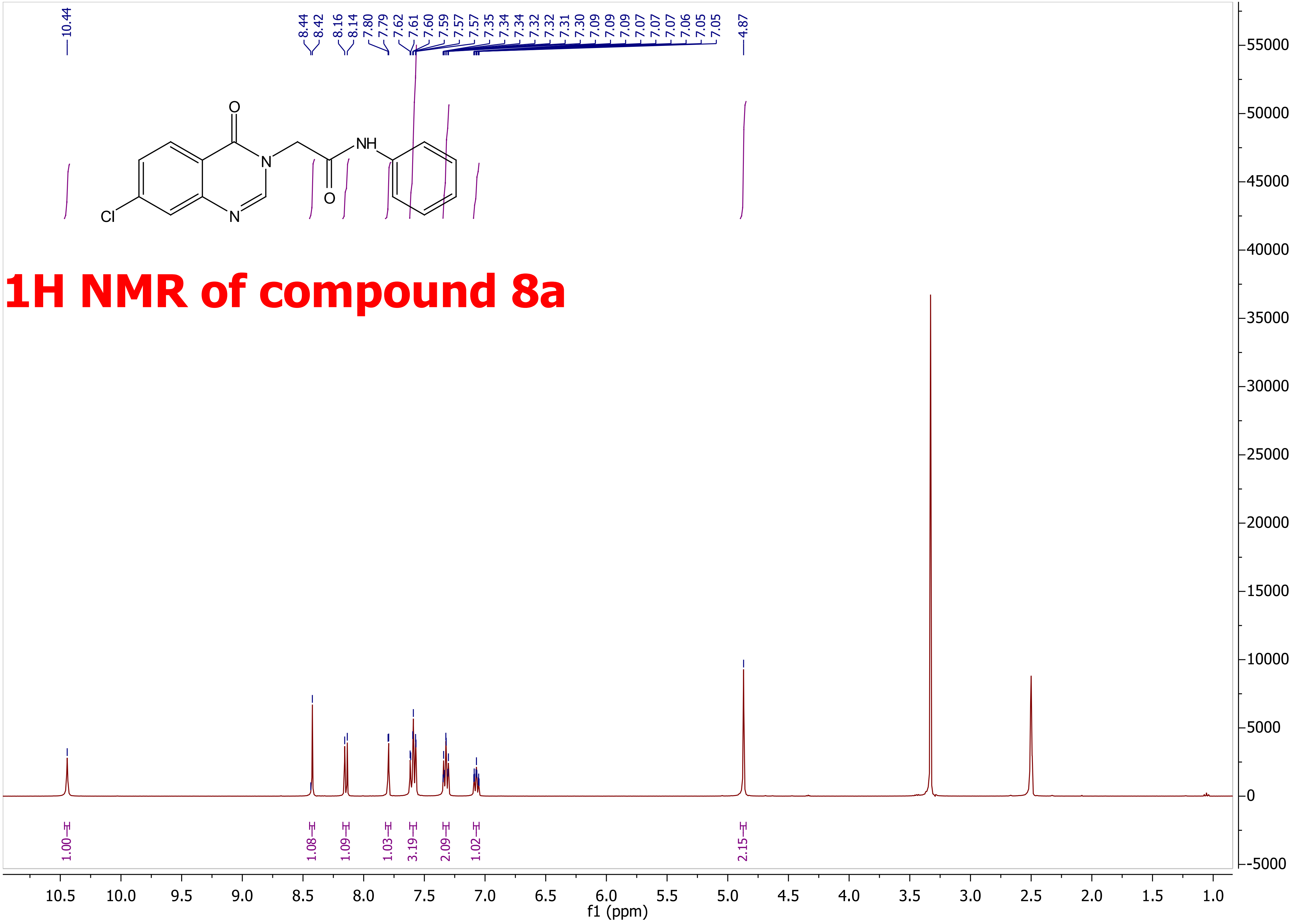

1H NMR of compound 8a

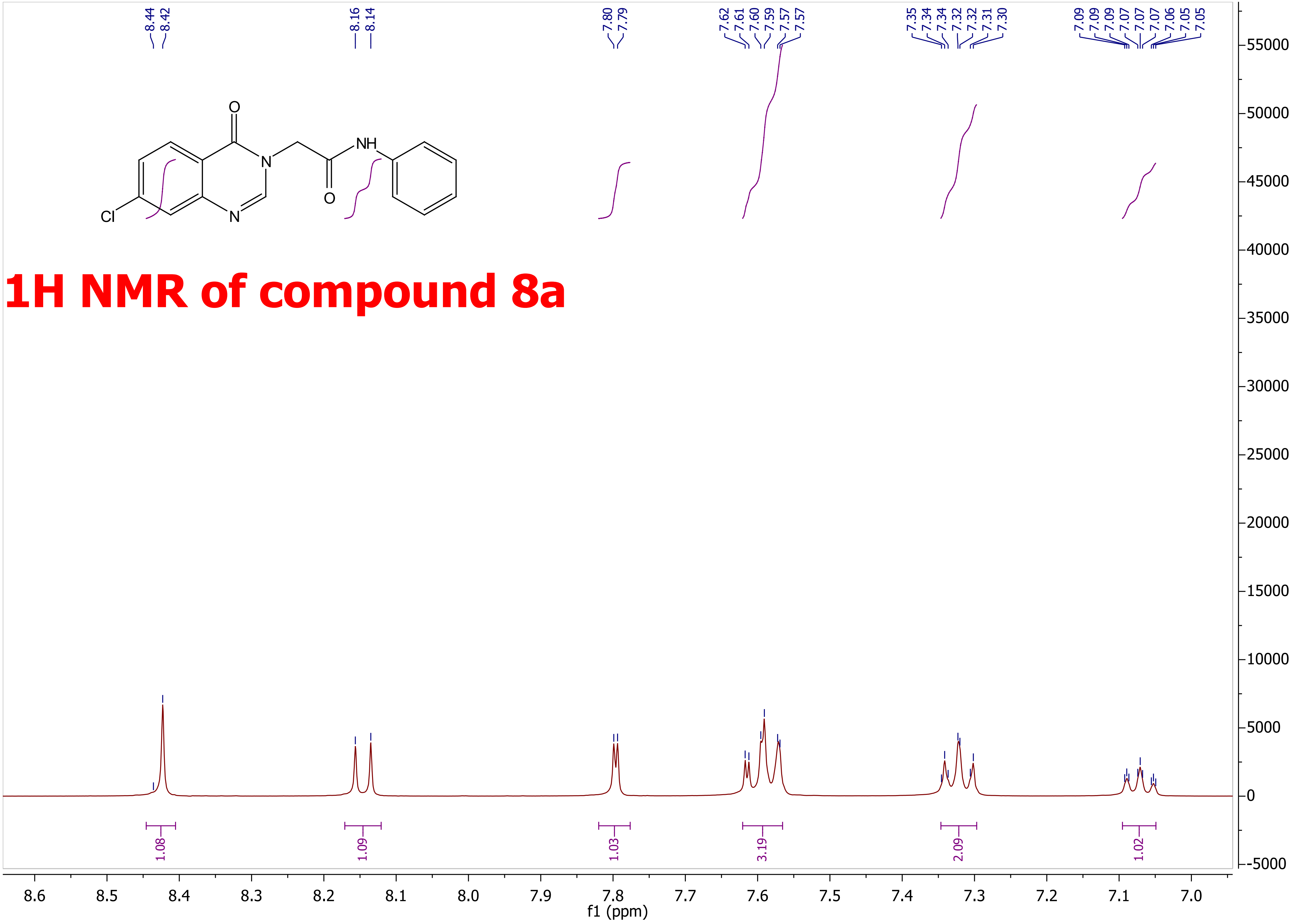
1H NMR of compound 8a
O
NH
N
O
Cl
N
8.44
8.42
8.16
8.14
7.80
7.79
7.62
7.61
7.60
7.59
7.57
7.57
7.35
7.34
7.34
7.32
7.32
7.31
7.30
7.09
7.09
7.09
7.07
7.07
7.07
7.06
7.05
7.05
1.08
1.09
1.03
3.19
2.09
1.02
f1 (ppm)

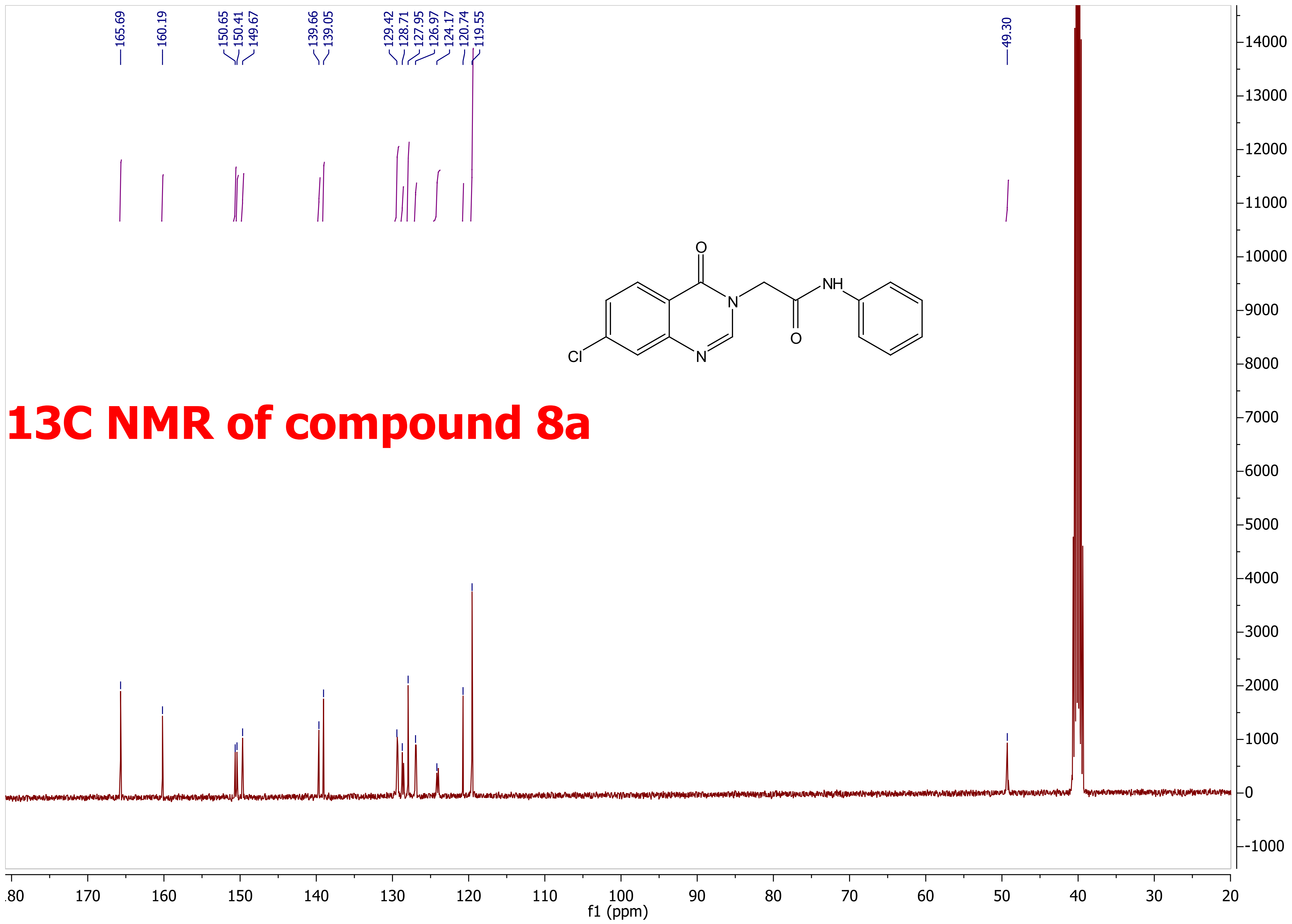

165.69
160.19
150.65
150.41
149.67
139.66
139.05
129.42
128.71
127.95
126.97
124.17
120.74
119.55
49.30
O
N
NH
O
Cl
N
13C NMR of compound 8a
14000
13000
12000
11000
10000
9000
8000
7000
6000
5000
4000
3000
2000
1000
0
-1000
80
170
160
150
140
130
120
110
100
90
80
70
60
50
40
30
20
f1 (ppm)

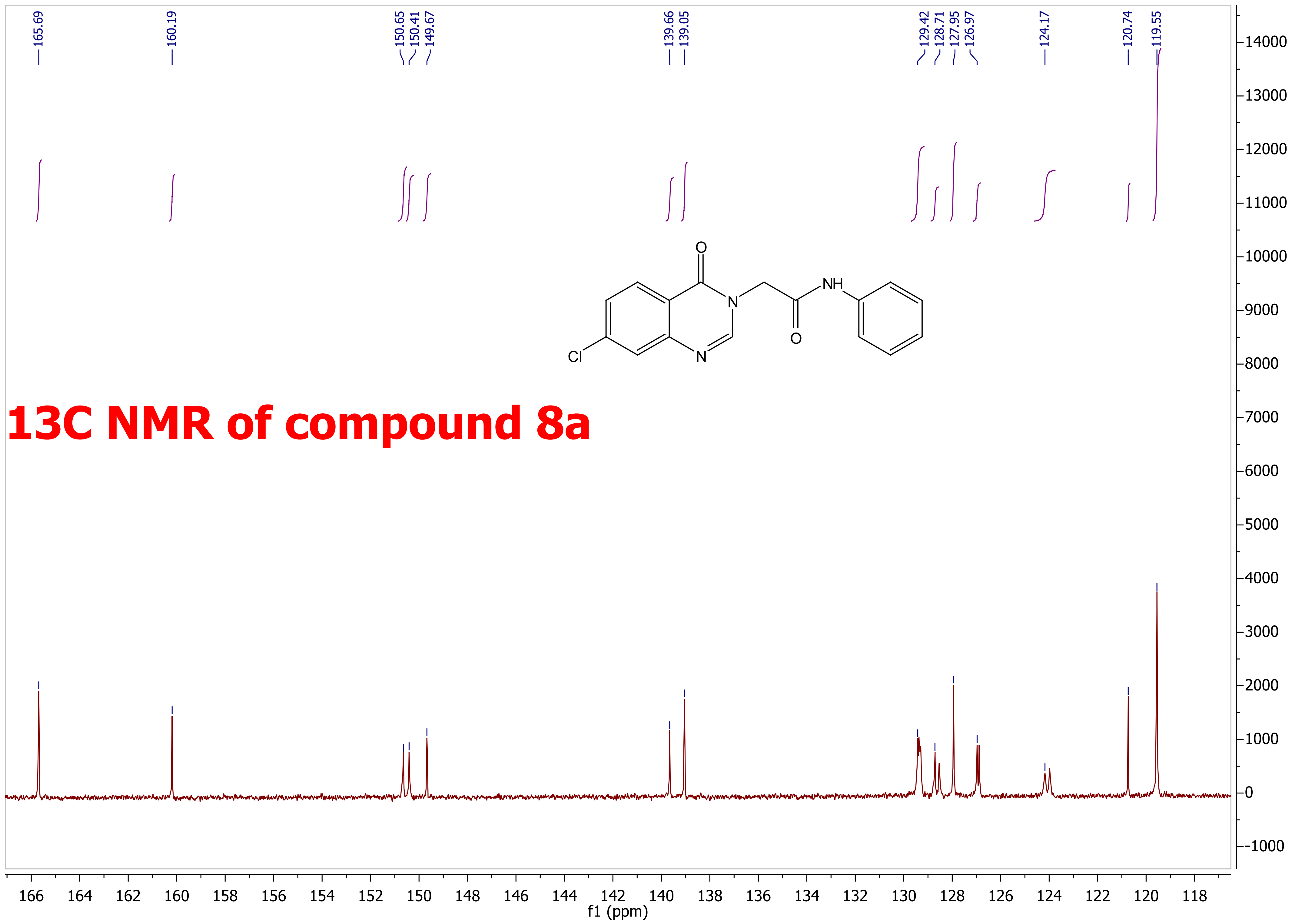
165.69
160.19
150.65
150.41
149.67
139.66
139.05
129.42
128.71
127.95
126.97
124.17
120.74
119.55
O
N
NH
O
Cl
N
13C NMR of compound 8a
14000
13000
12000
11000
10000
9000
8000
7000
6000
5000
4000
3000
2000
1000
0
-1000
166
164
162
160
158
156
154
152
150
148
146
144
142
140
138
136
134
132
130
128
126
124
122
120
118
f1 (ppm)

IR of compound 8b

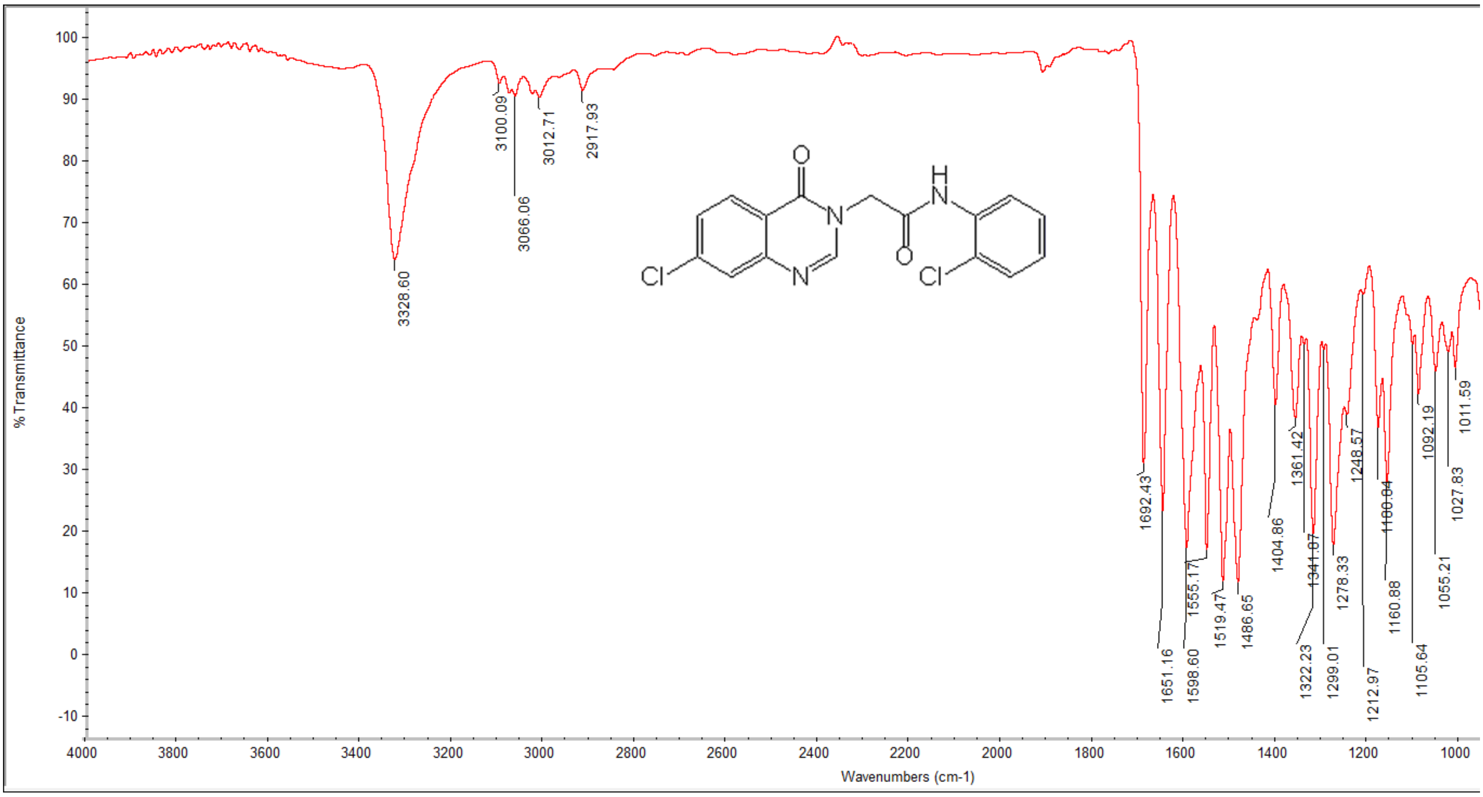

1H NMR of compound 8b

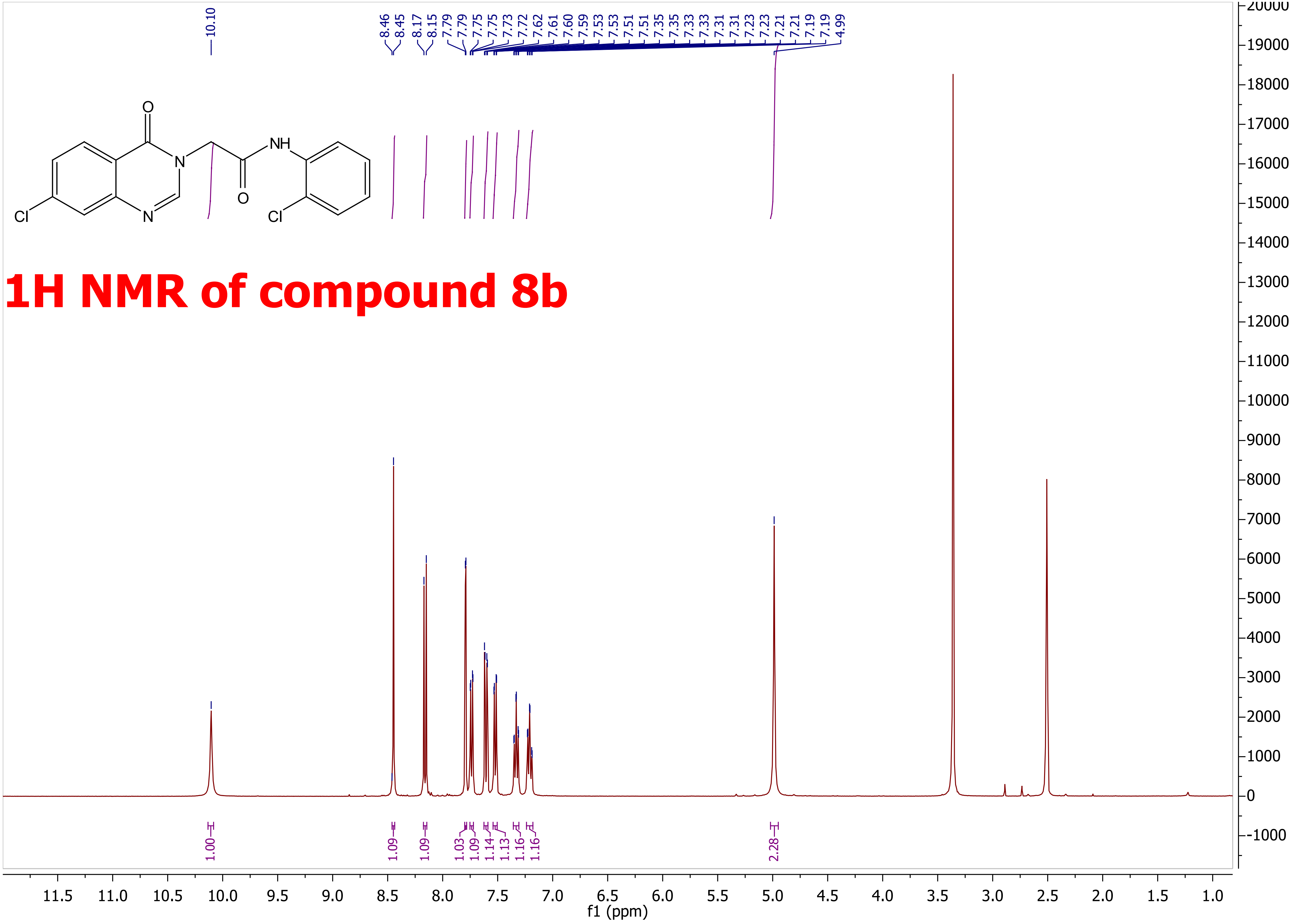

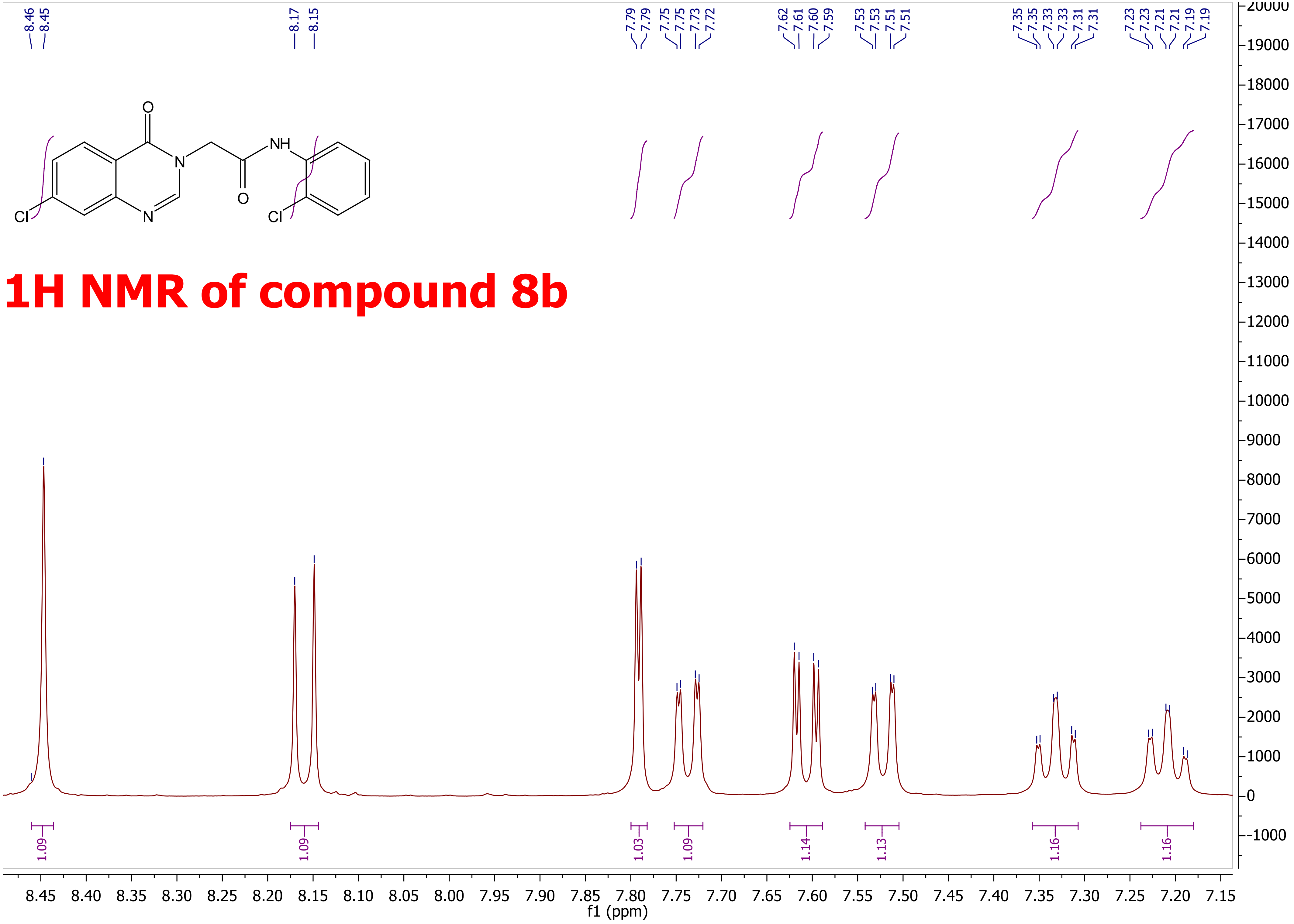
1H NMR of compound 8b
8.46
8.45
8.17
8.15
7.79
7.79
7.75
7.75
7.73
7.72
7.62
7.61
7.60
7.59
7.53
7.53
7.51
7.51
7.35
7.35
7.33
7.33
7.31
7.31
7.23
7.23
7.21
7.21
7.19
7.19
O
N
NH
O
Cl
N
Cl
1.09
1.09
1.03
1.09
1.14
1.13
1.16
1.16
f1 (ppm)

1H NMR of compound 8b

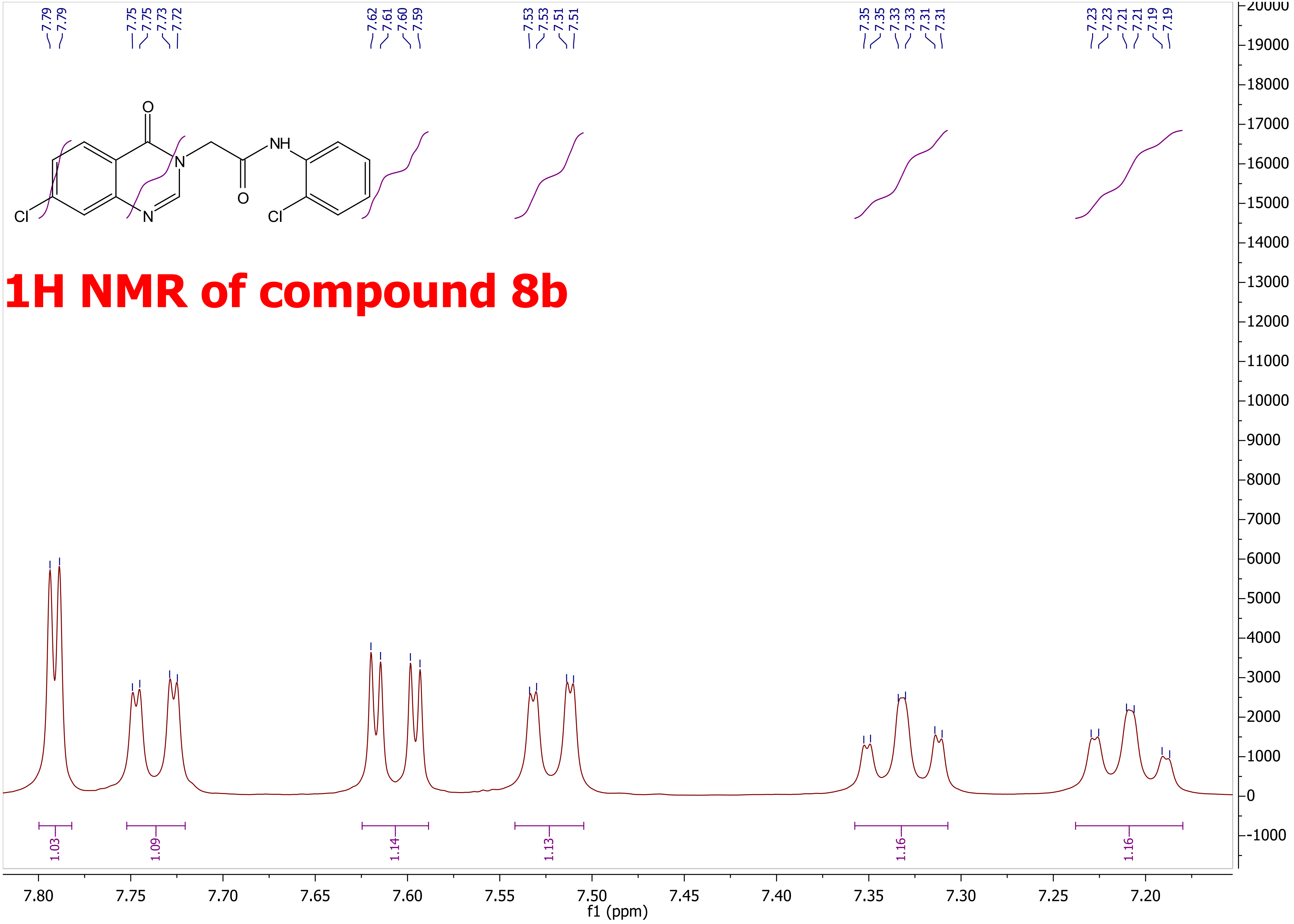

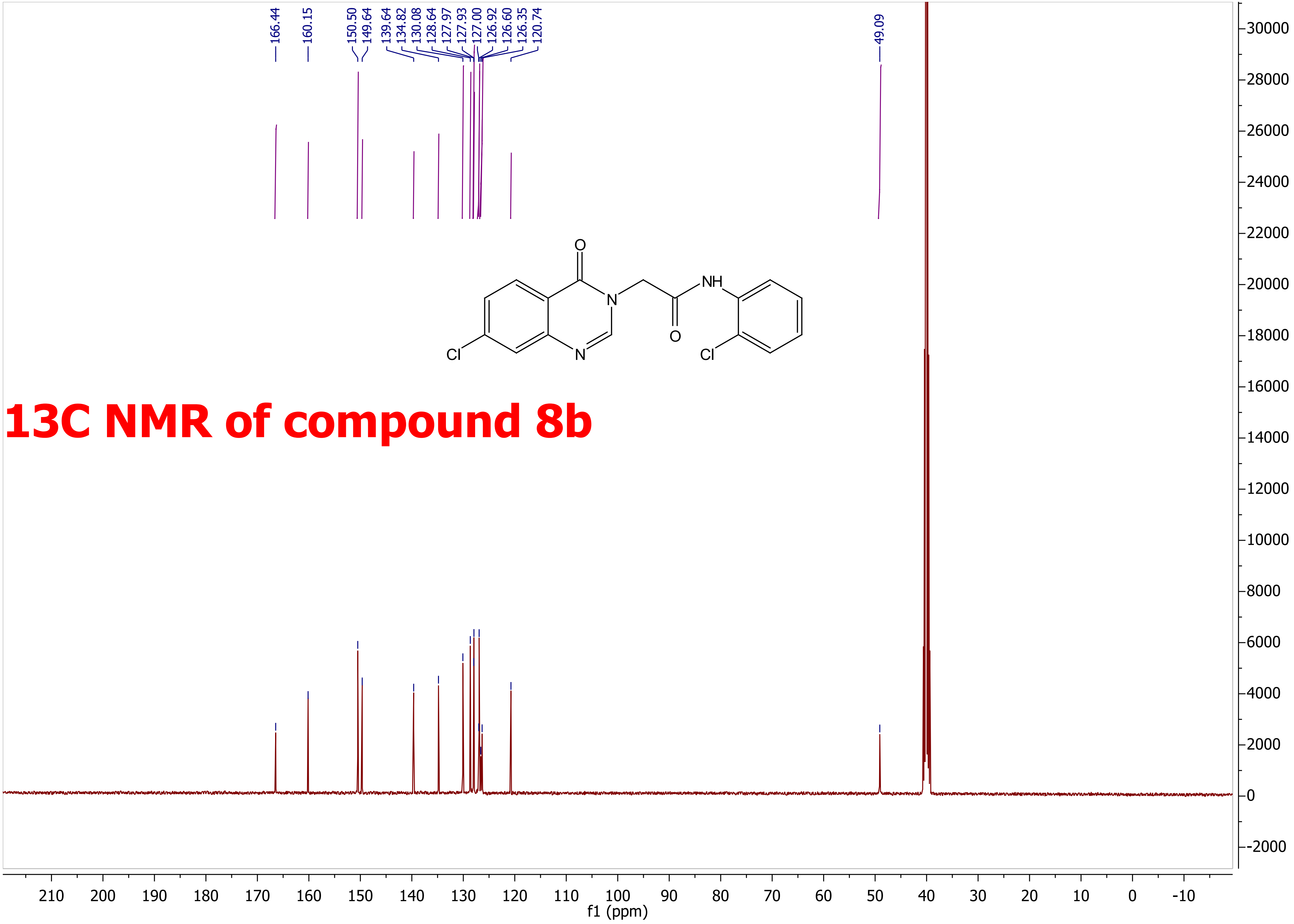

13C NMR of compound 8b
166.44
160.15
150.50
149.64
139.64
134.82
130.08
128.64
127.97
127.93
127.00
126.92
126.60
126.35
120.74
49.09
O
N
NH
O
Cl
N
Cl
f1 (ppm)

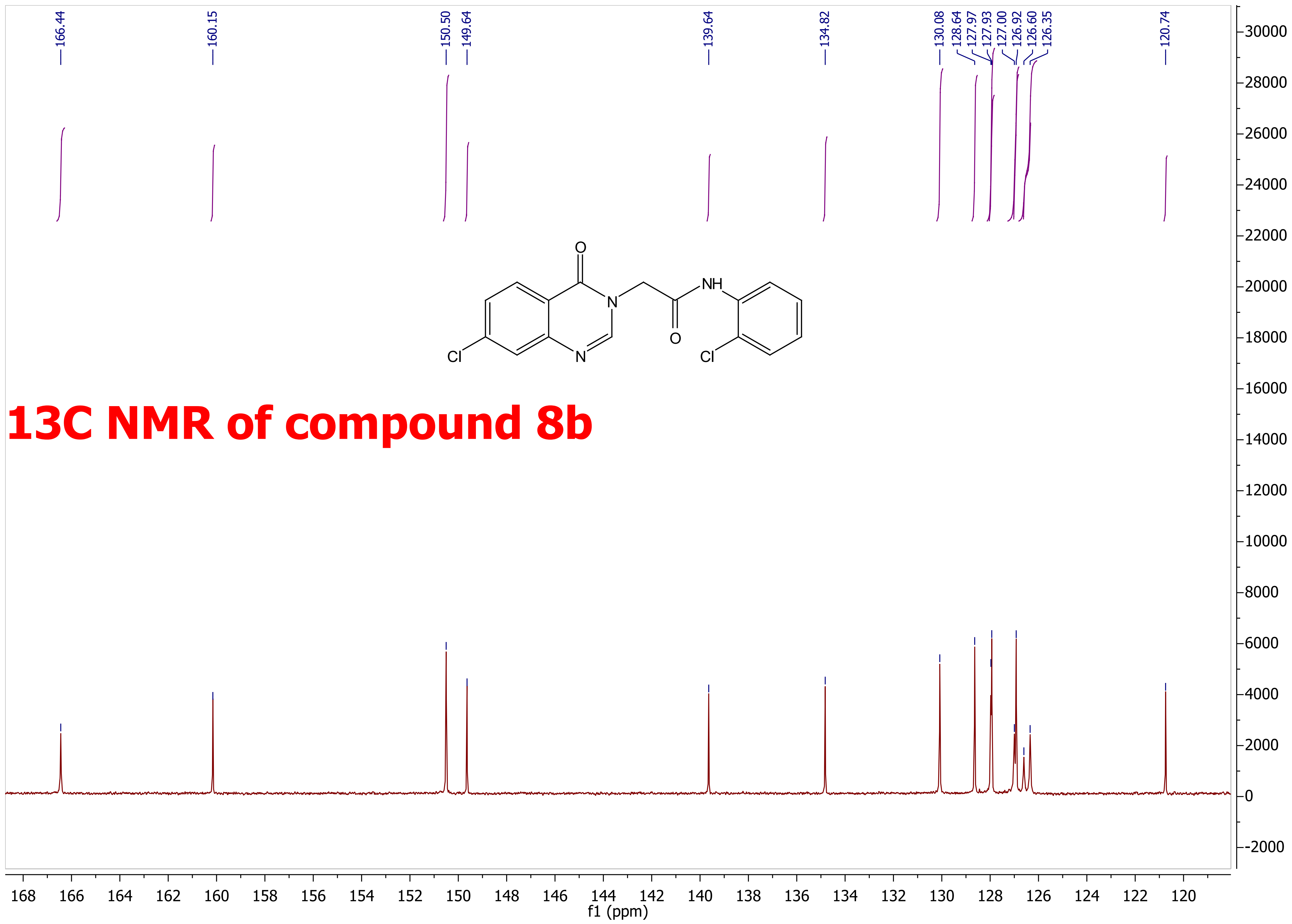

13C NMR of compound 8b

IR of compound 8c

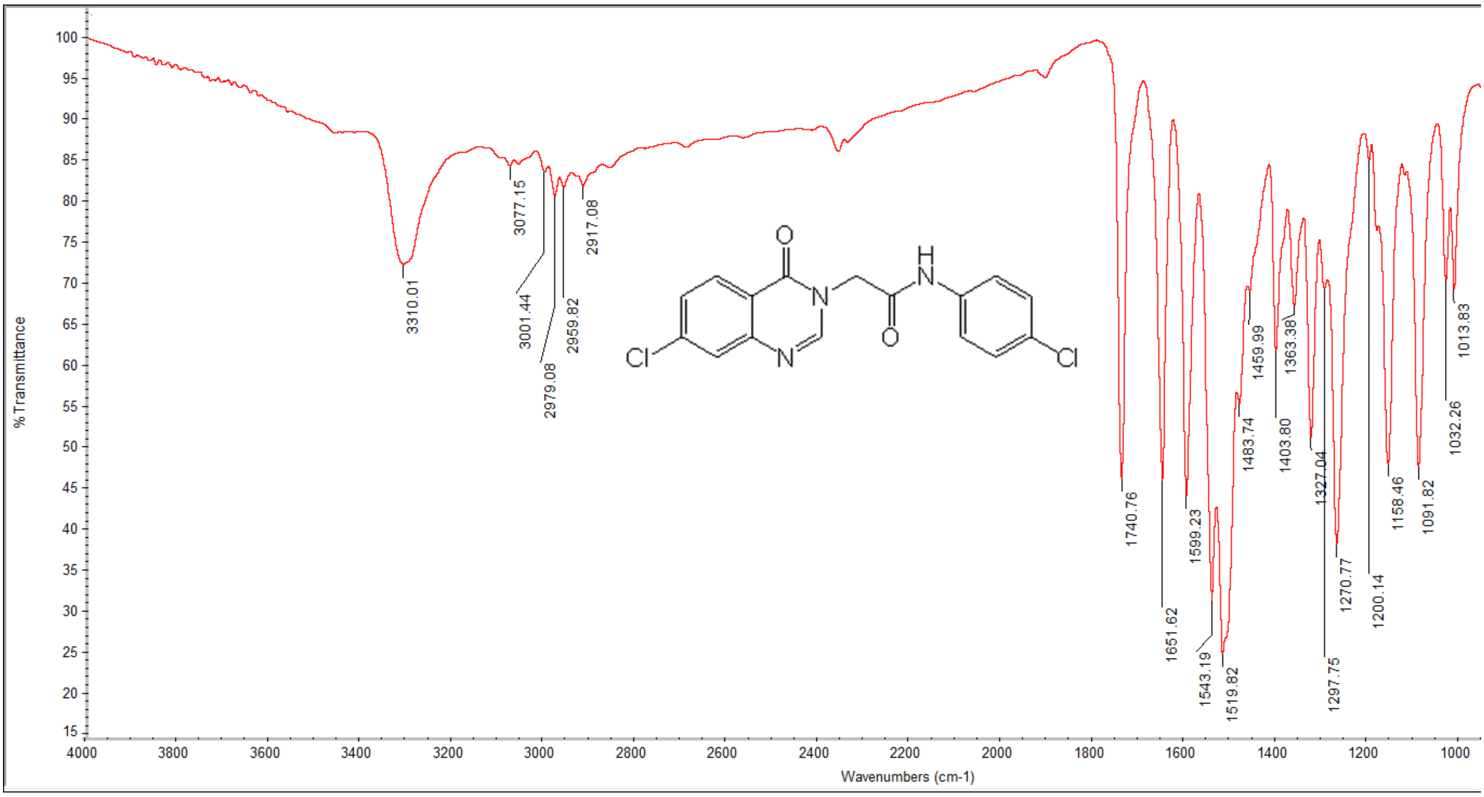

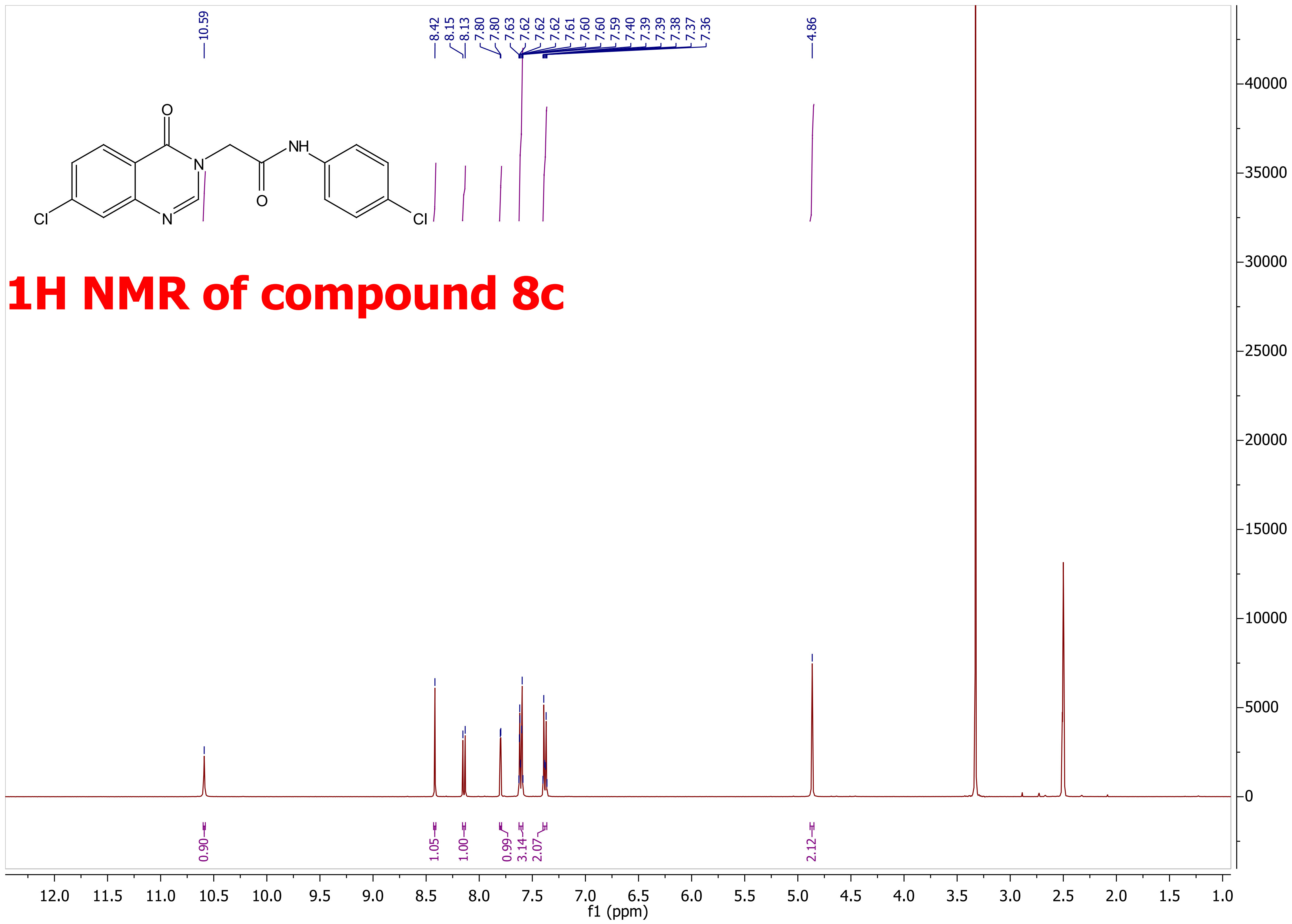
1H NMR of compound 8c
10.59
8.42
8.15
8.13
7.80
7.80
7.63
7.62
7.62
7.62
7.61
7.60
7.60
7.59
7.40
7.39
7.39
7.38
7.37
7.36
4.86
0.90
1.05
1.00
0.99
3.14
2.07
2.12
f1 (ppm)

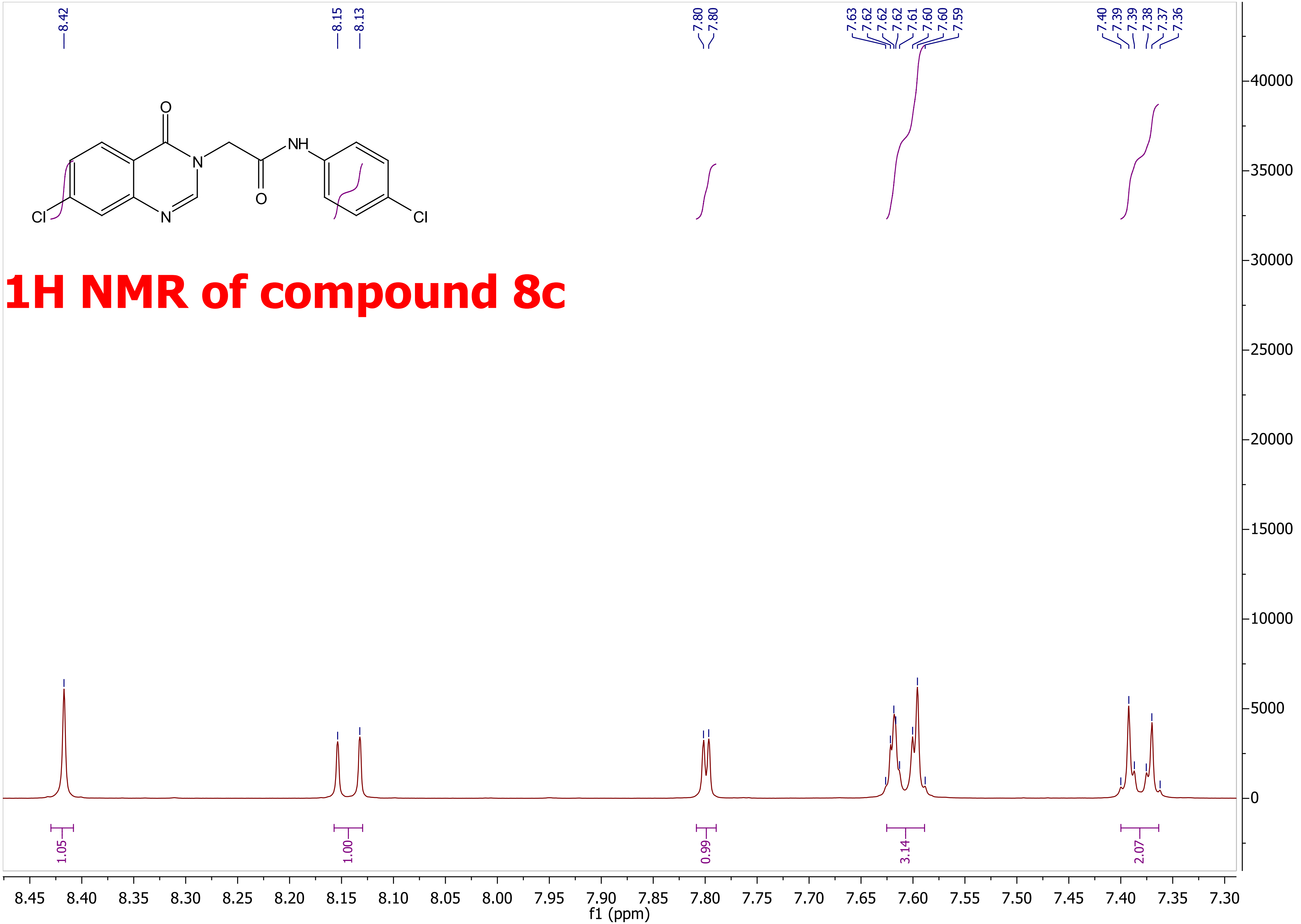
1H NMR of compound 8c
8.42
8.15
8.13
7.80
7.80
7.63
7.62
7.62
7.62
7.61
7.60
7.60
7.59
7.40
7.39
7.39
7.38
7.37
7.36
O
NH
N
N
O
Cl
Cl
1.05
1.00
0.99
3.14
2.07
40000
35000
30000
25000
20000
15000
10000
5000
0
8.45 8.40 8.35 8.30 8.25 8.20 8.15 8.10 8.05 8.00 7.95 7.90 7.85 7.80 7.75 7.70 7.65 7.60 7.55 7.50 7.45 7.40 7.35 7.30
f1 (ppm)

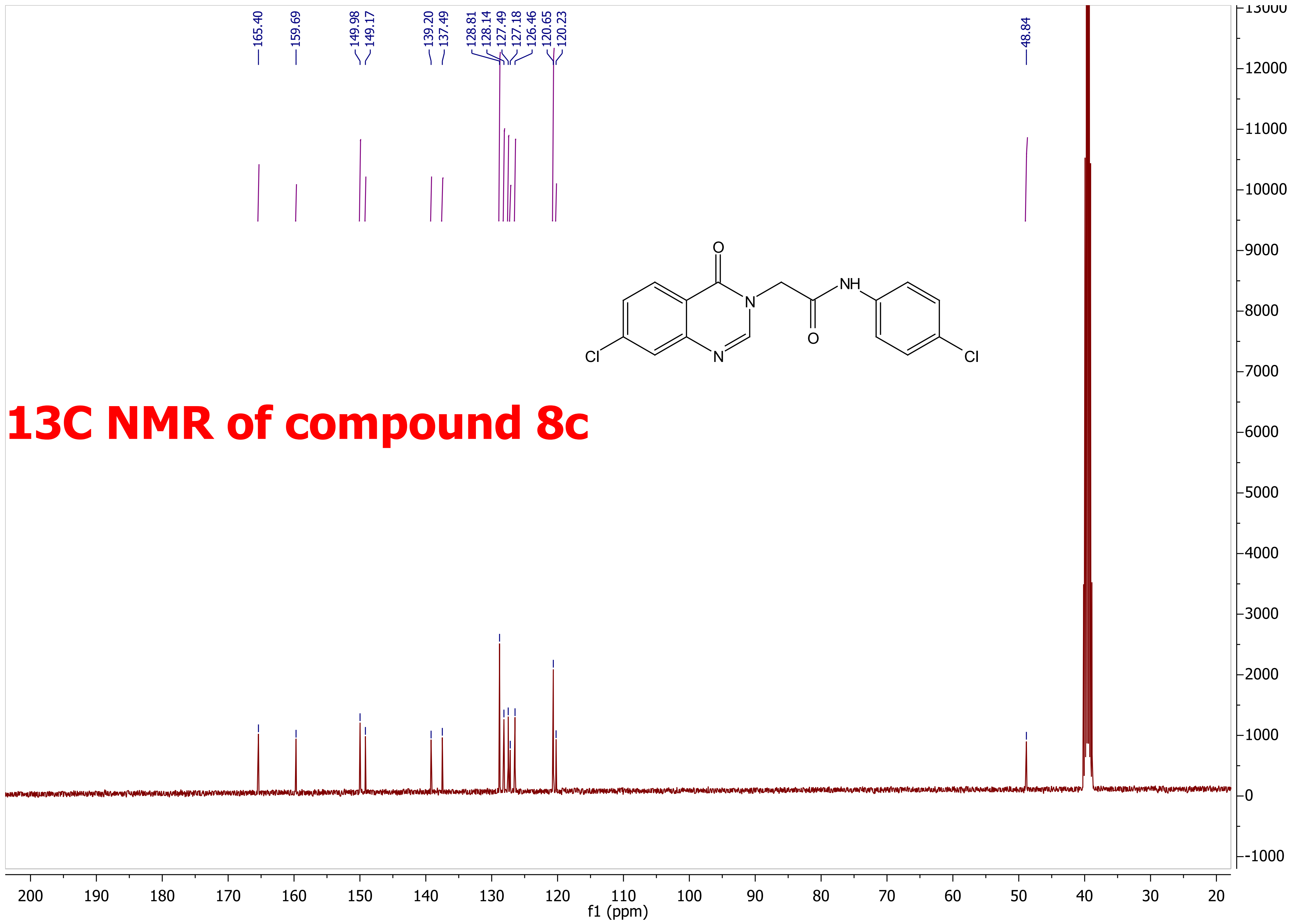
165.40
159.69
149.98
149.17
139.20
137.49
128.81
128.14
127.49
127.18
126.46
120.65
120.23
48.84
O
N
NH
O
Cl
N
Cl
13C NMR of compound 8c
f1 (ppm)

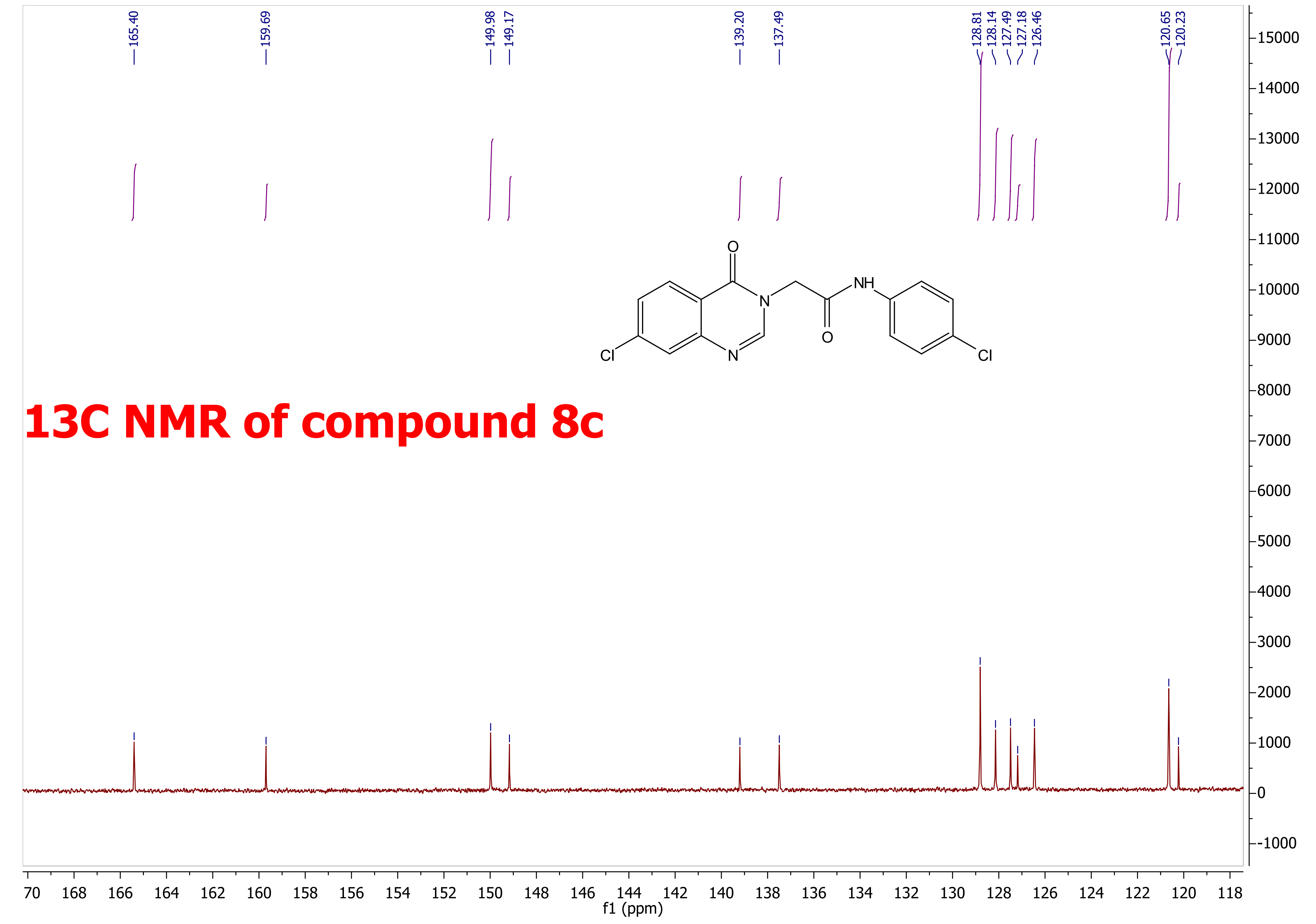

13C NMR of compound 8c

IR of compound 8d

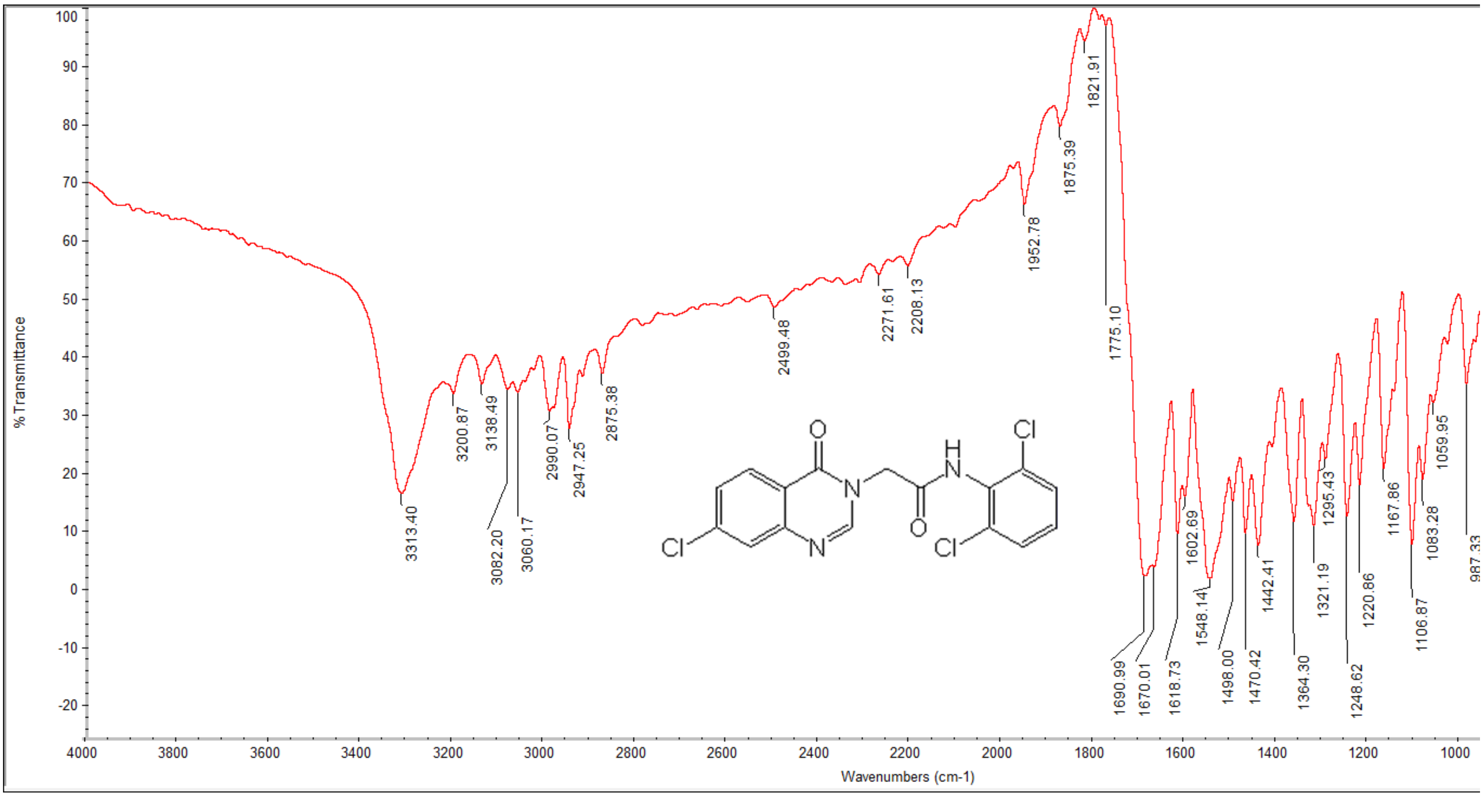

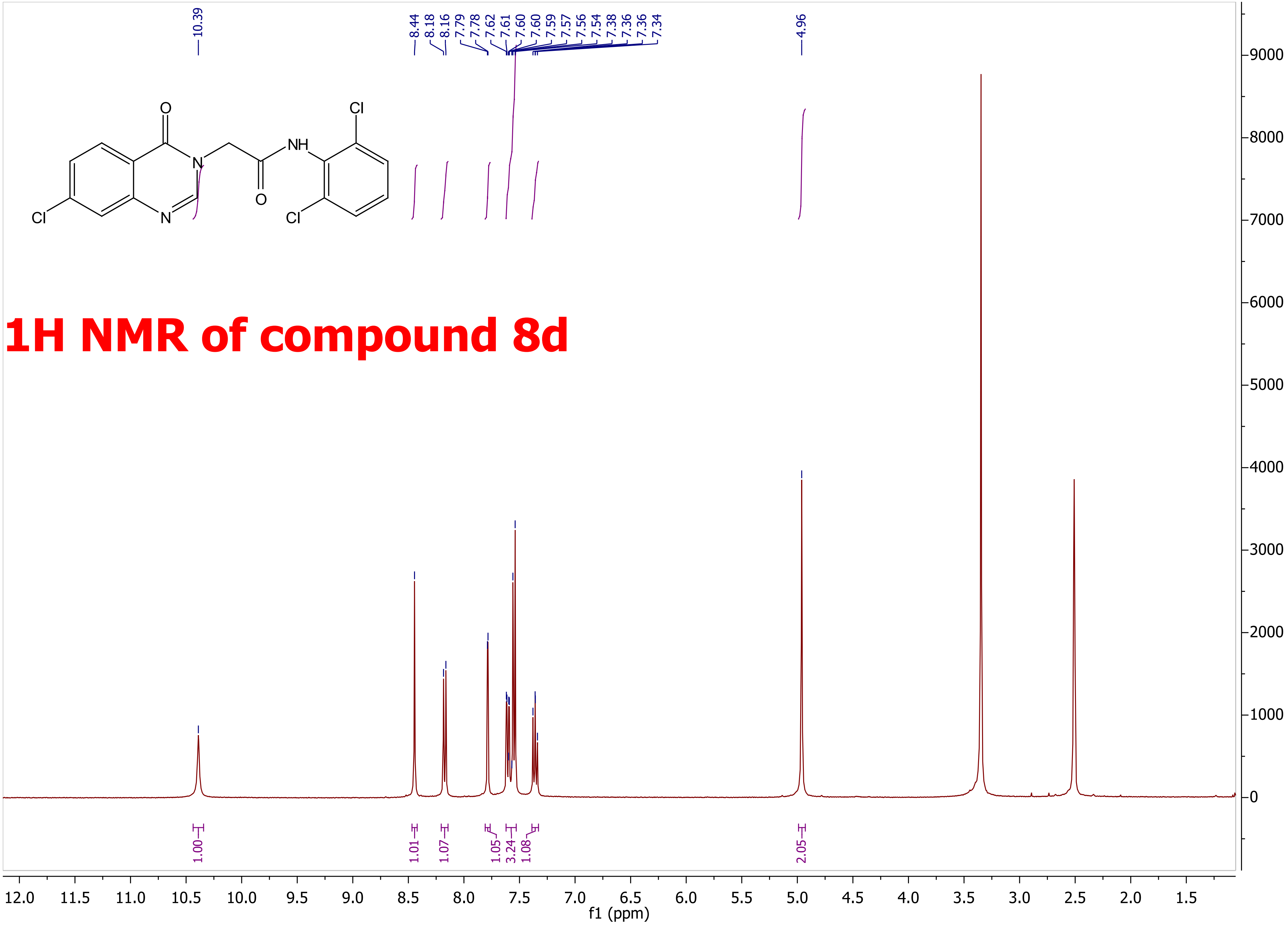
1H NMR of compound 8d
10.39
8.44
8.18
8.16
7.79
7.78
7.62
7.61
7.60
7.60
7.59
7.57
7.56
7.54
7.38
7.36
7.36
7.34
4.96
1.00
1.01
1.07
1.05
3.24
1.08
2.05
Cl
O
N
N
NH
O
Cl
Cl
f1 (ppm)

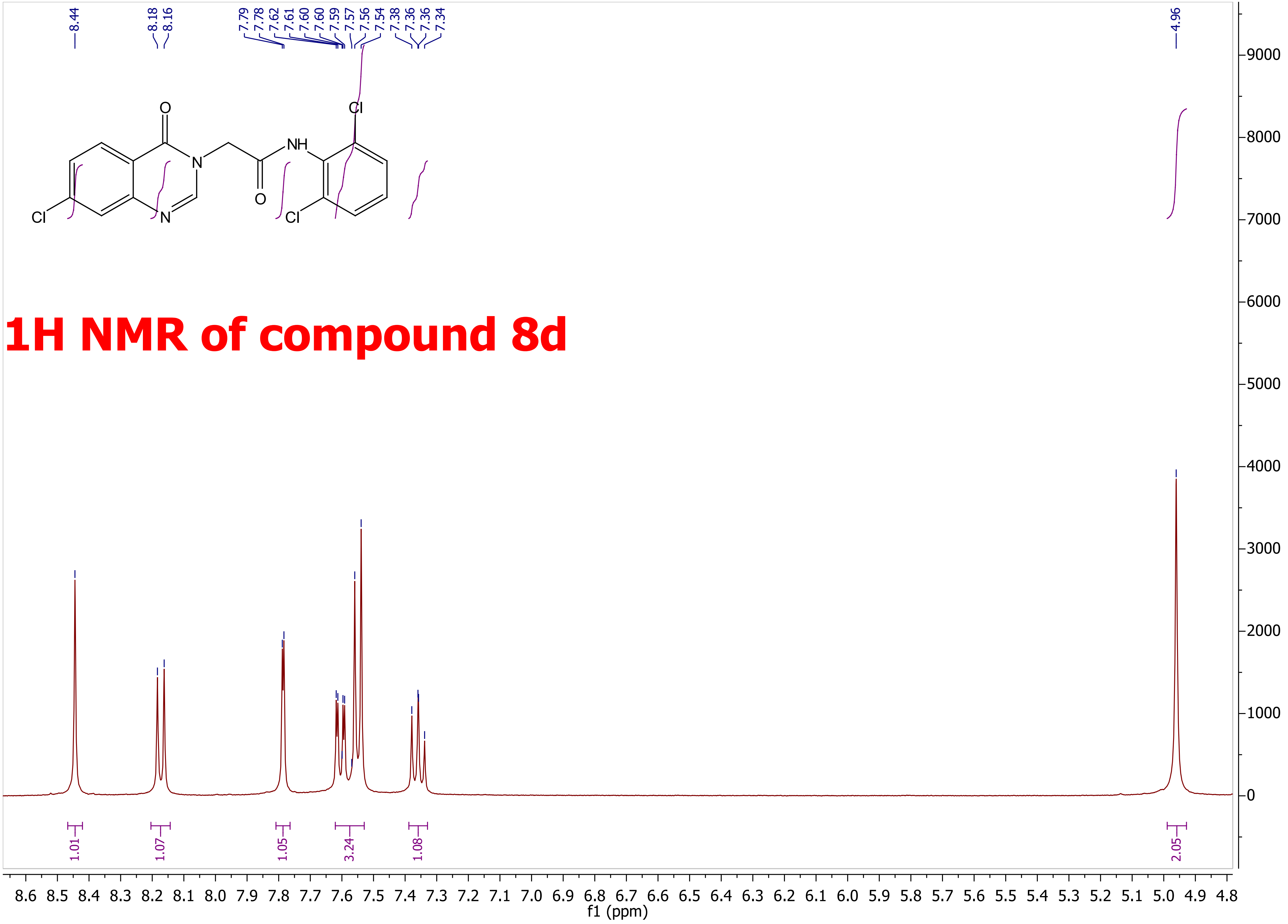
1H NMR of compound 8d
8.44
8.18
8.16
7.79
7.78
7.62
7.61
7.60
7.60
7.59
7.57
7.56
7.54
7.38
7.36
7.36
7.34
4.96
O
N
N
O
NH
Cl
Cl
Cl
1.01
1.07
1.05
3.24
1.08
2.05
9000
8000
7000
6000
5000
4000
3000
2000
1000
0
f1 (ppm)

1H NMR of compound 8d

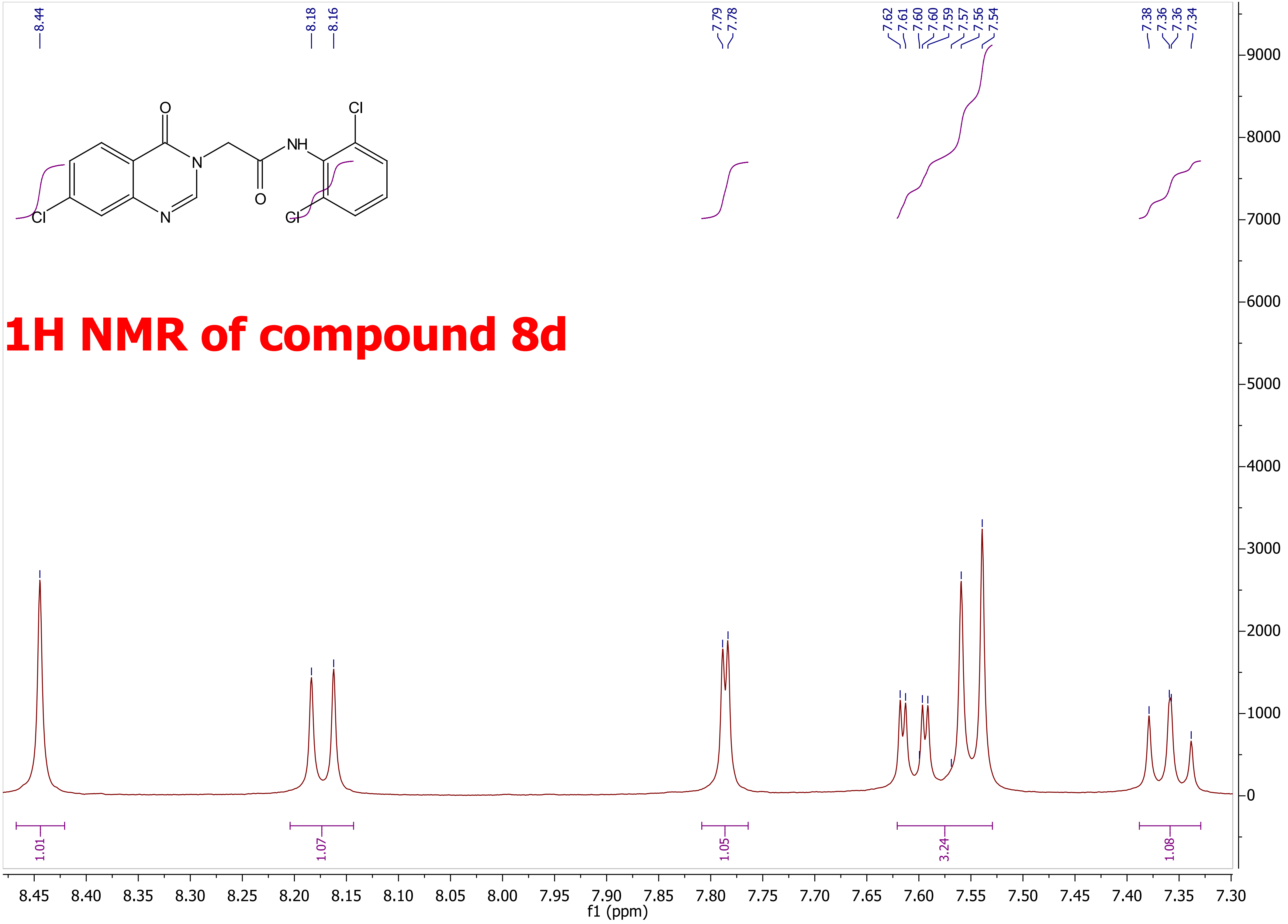

13C NMR of compound 8d

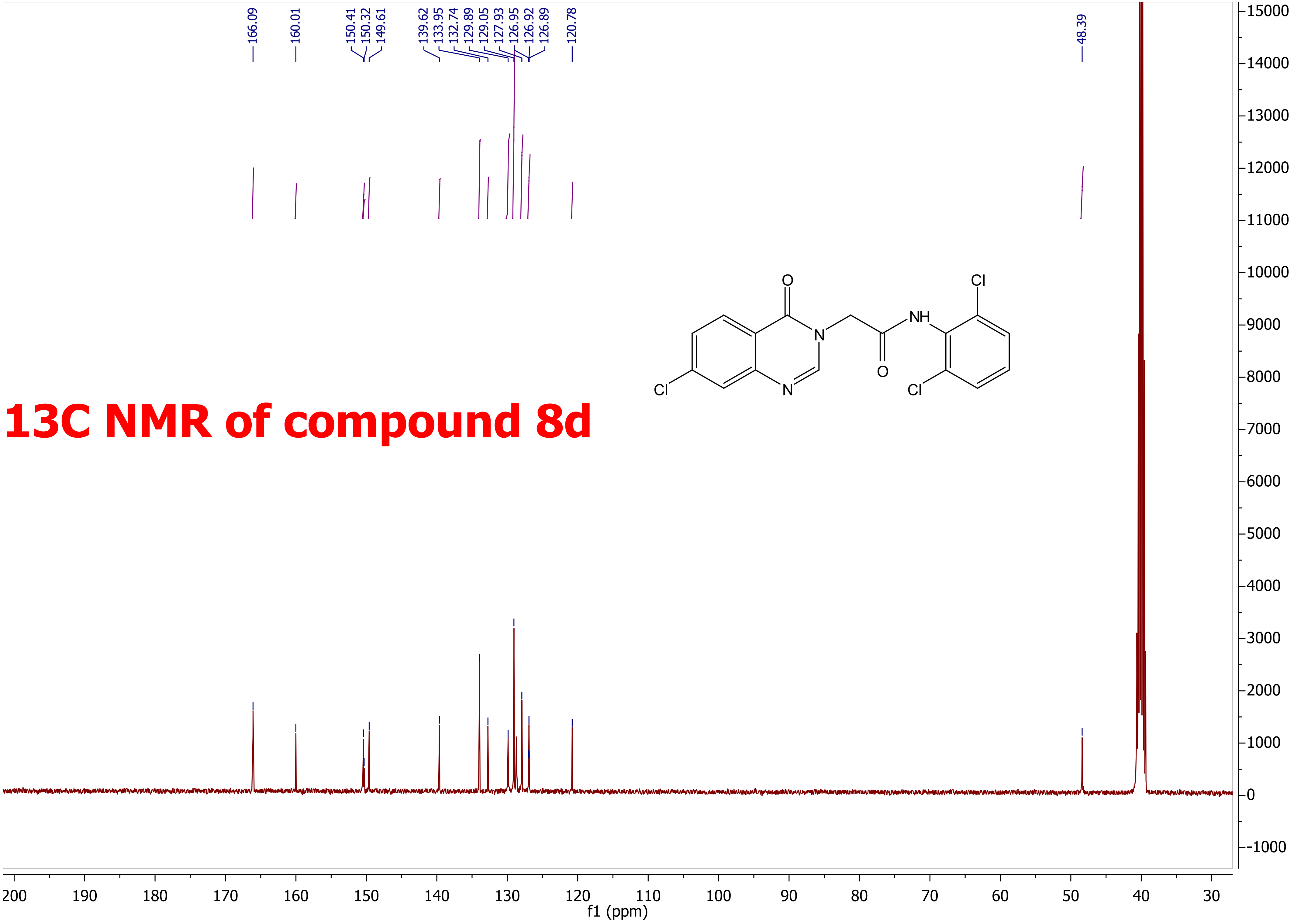

13C NMR of compound 8d

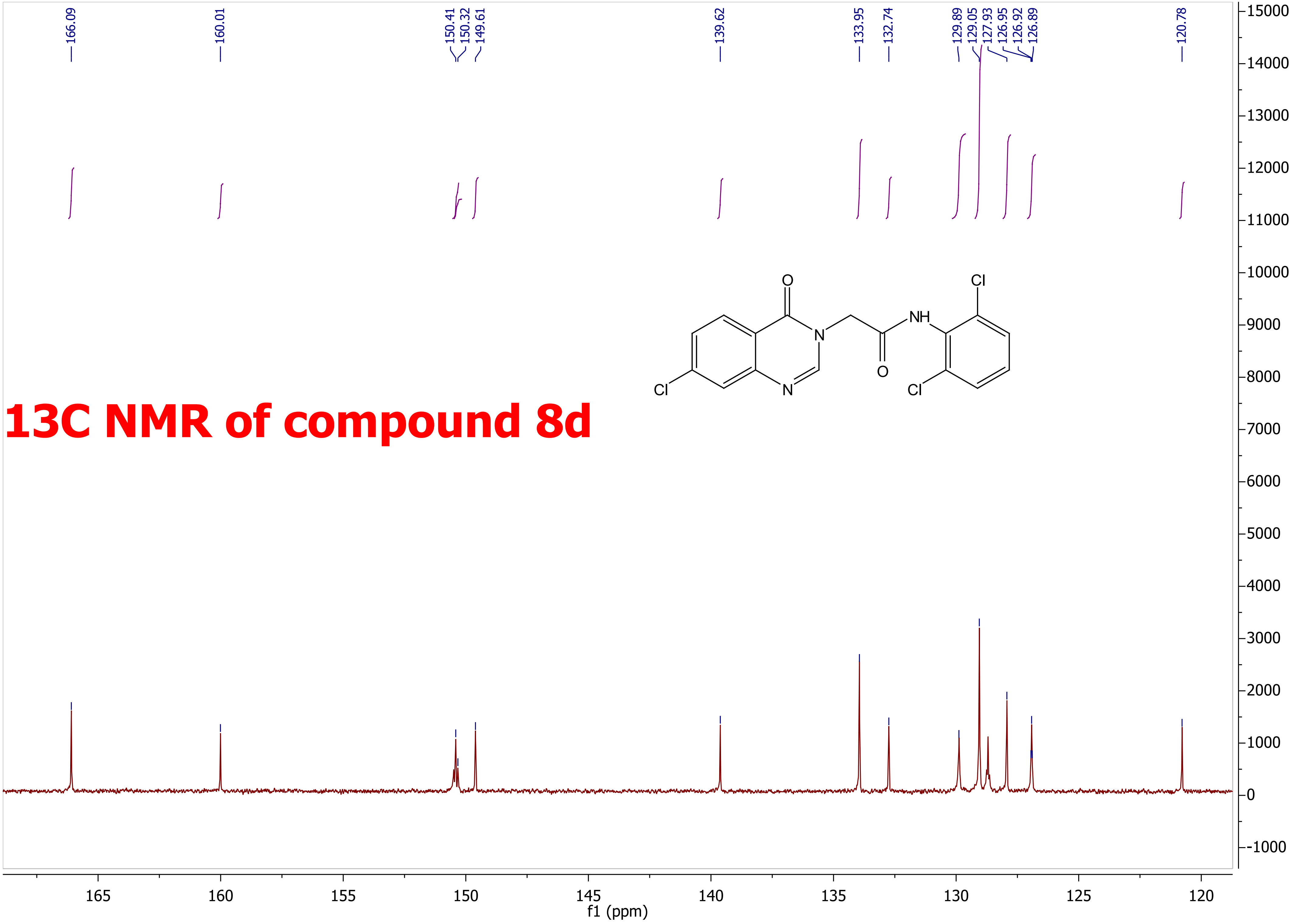

IR of compound 8e

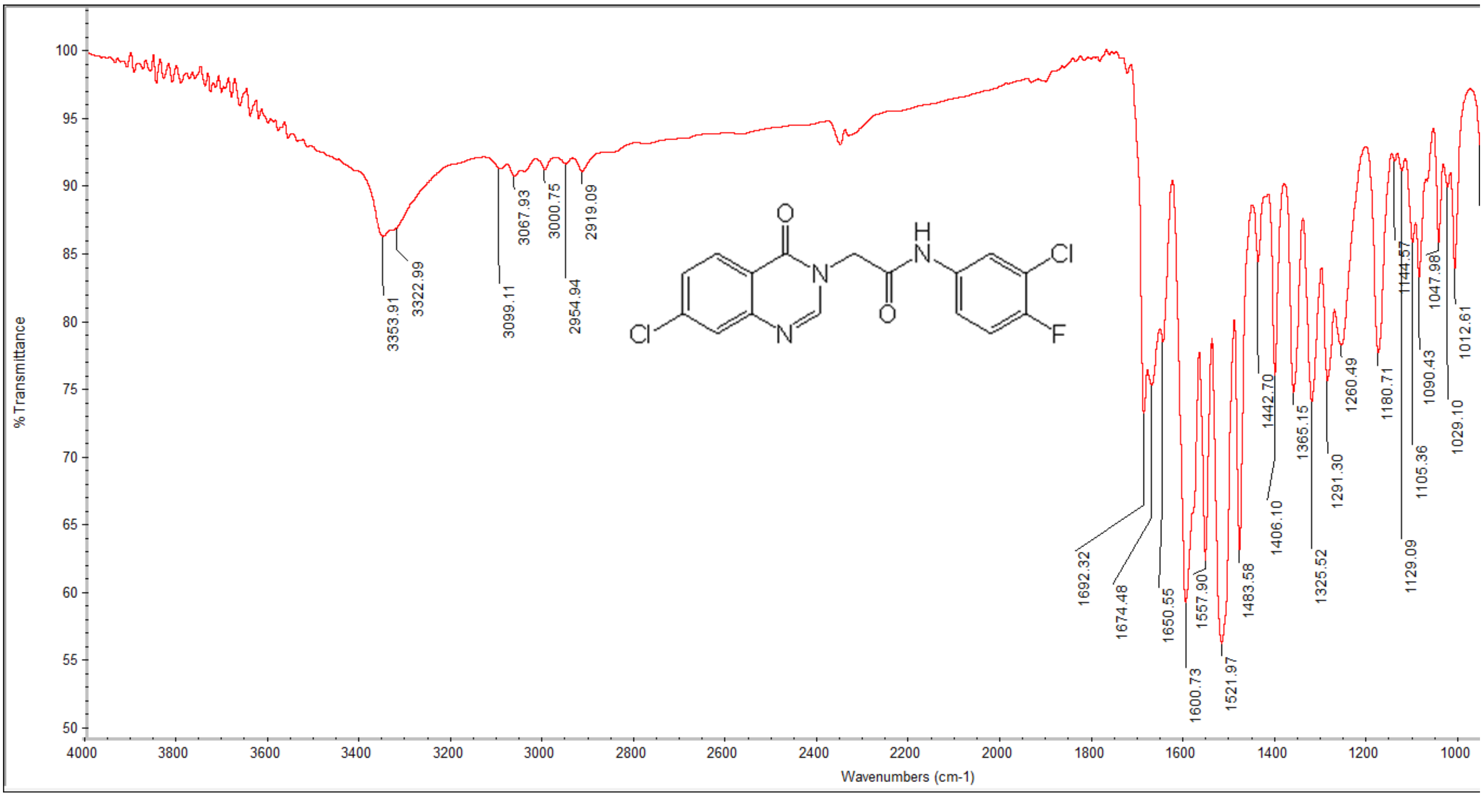

1H NMR of compound 8e

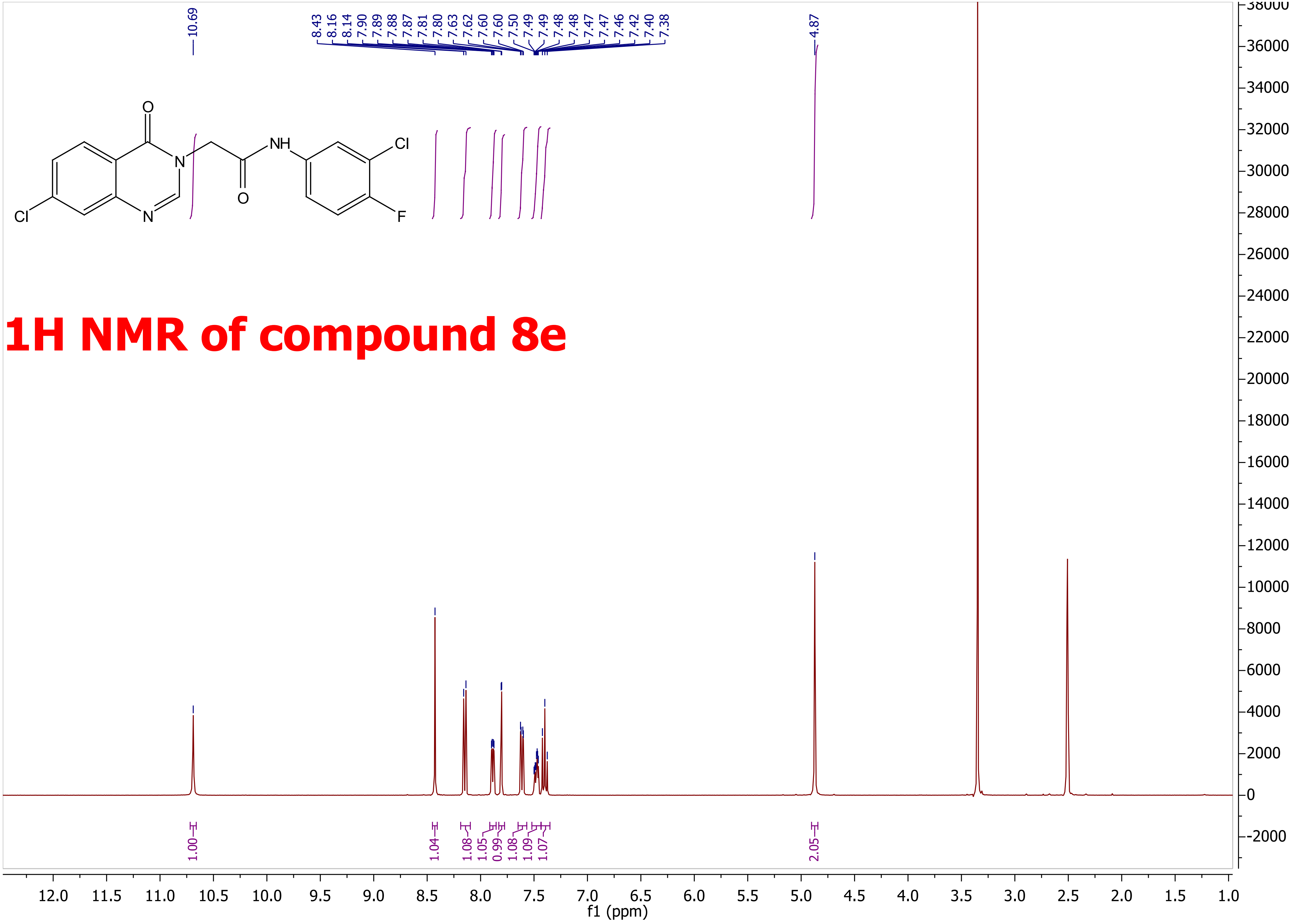

1H NMR of compound 8e

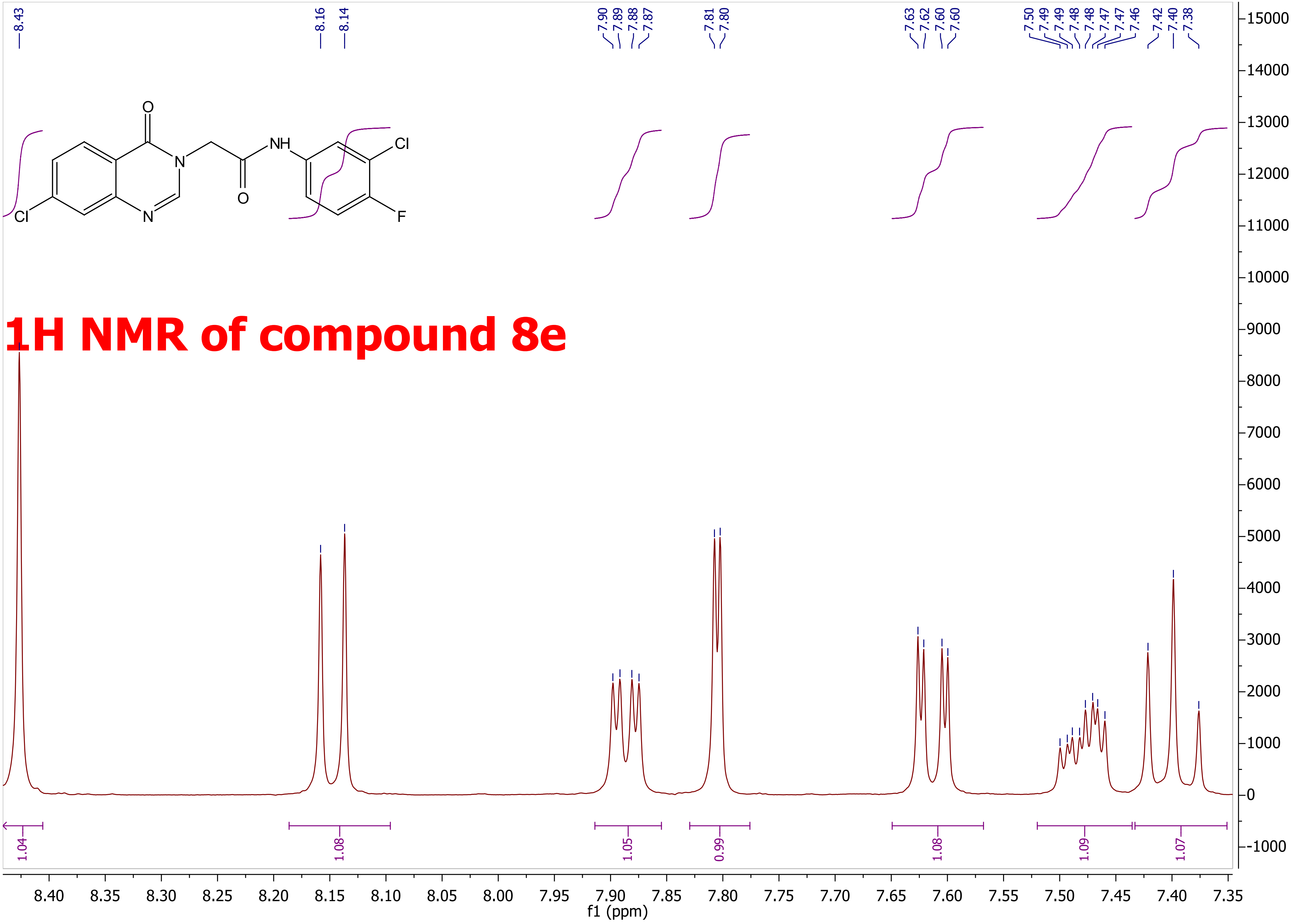

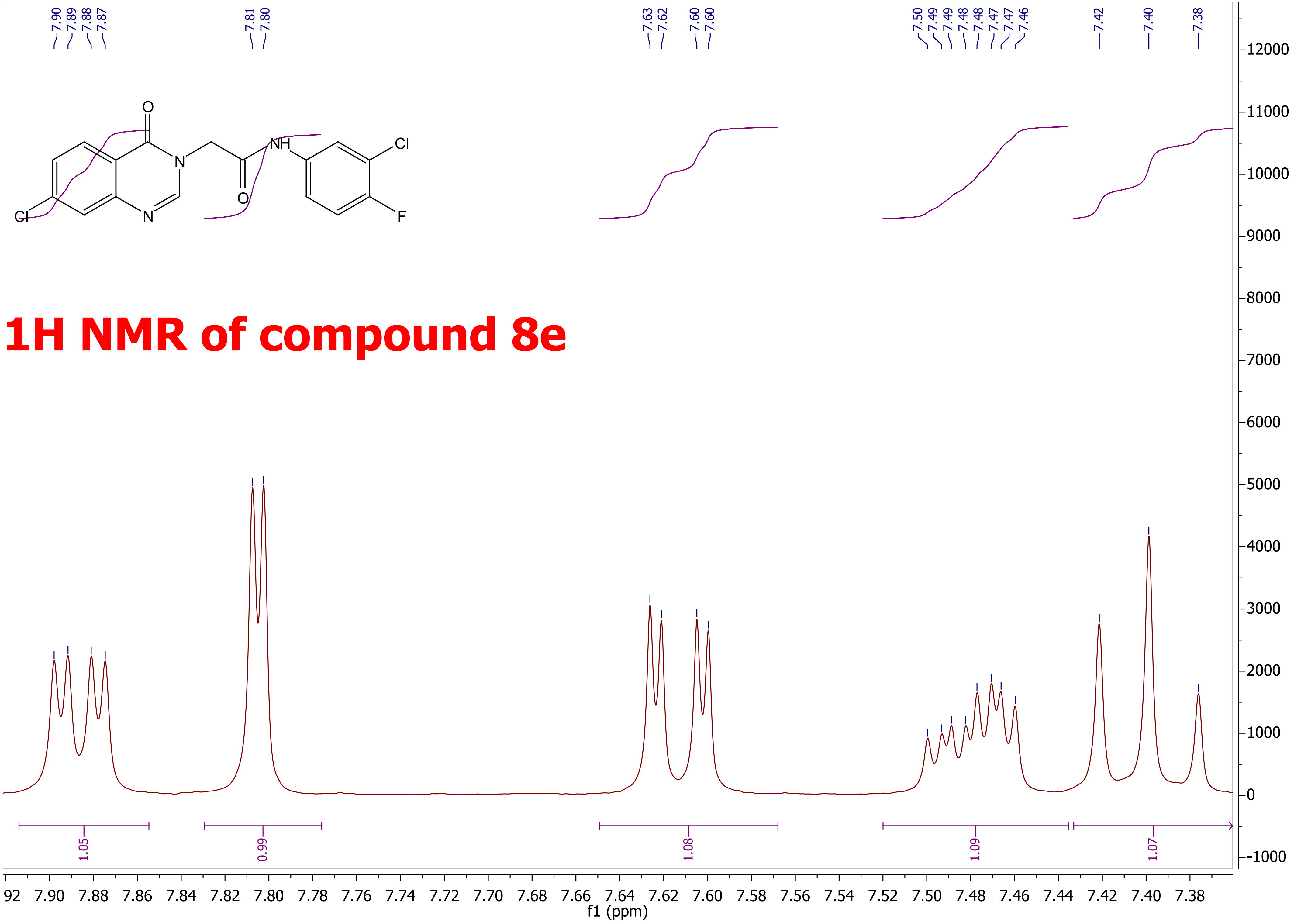
1H NMR of compound 8e
7.90
7.89
7.88
7.87
7.81
7.80
7.63
7.62
7.60
7.60
7.50
7.49
7.49
7.48
7.48
7.47
7.47
7.46
7.42
7.40
7.38
O
N
NH
Cl
O
Cl
N
F
1.05
0.99
1.08
1.09
1.07
f1 (ppm)

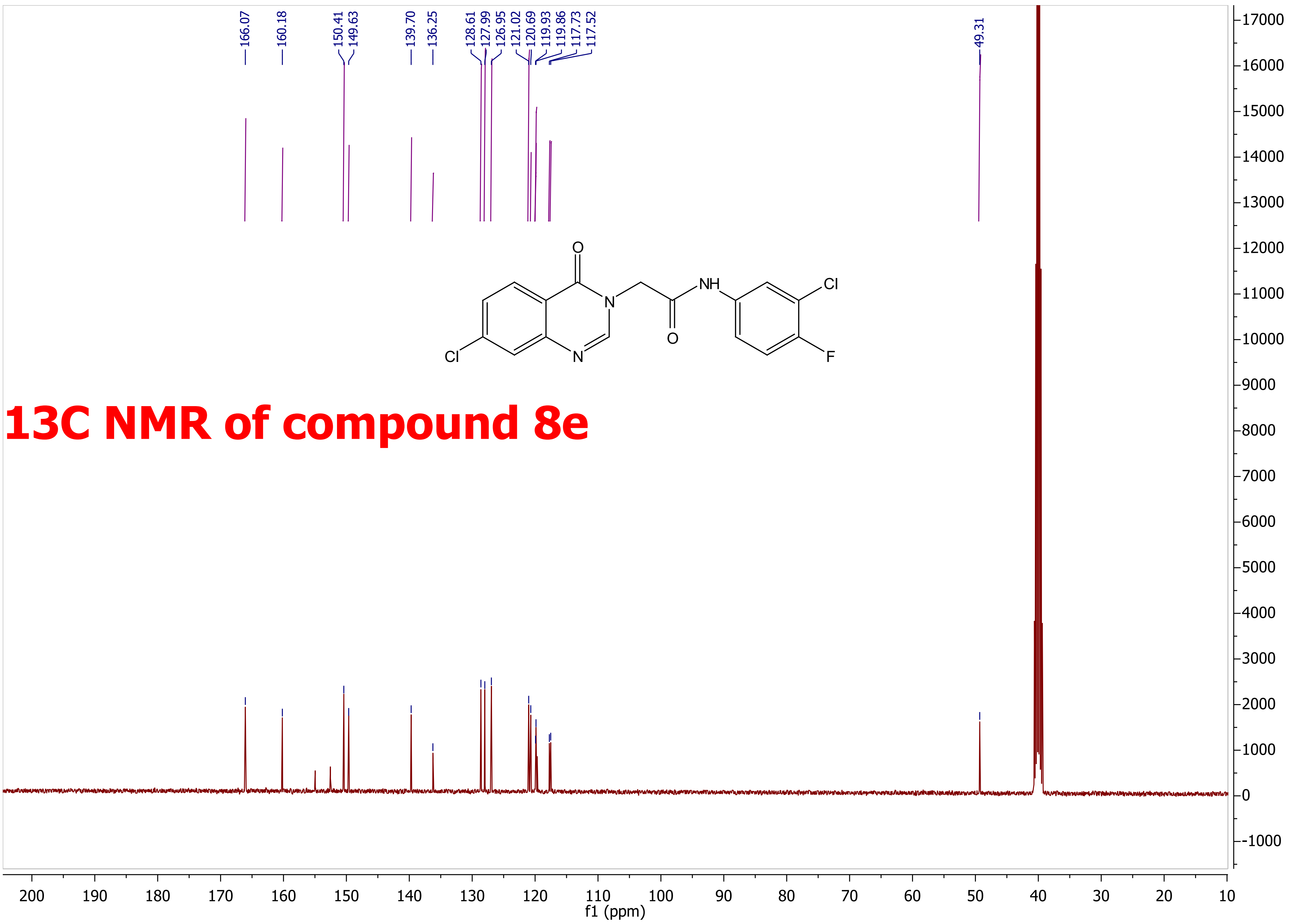
13C NMR of compound 8e
166.07
160.18
150.41
149.63
139.70
136.25
128.61
127.99
126.95
121.02
120.69
119.93
119.86
117.73
117.52
49.31
O
N
NH
Cl
O
Cl
N
F
17000
16000
15000
14000
13000
12000
11000
10000
9000
8000
7000
6000
5000
4000
3000
2000
1000
0
-1000
200
190
180
170
160
150
140
130
120
110
100
90
80
70
60
50
40
30
20
10
f1 (ppm)

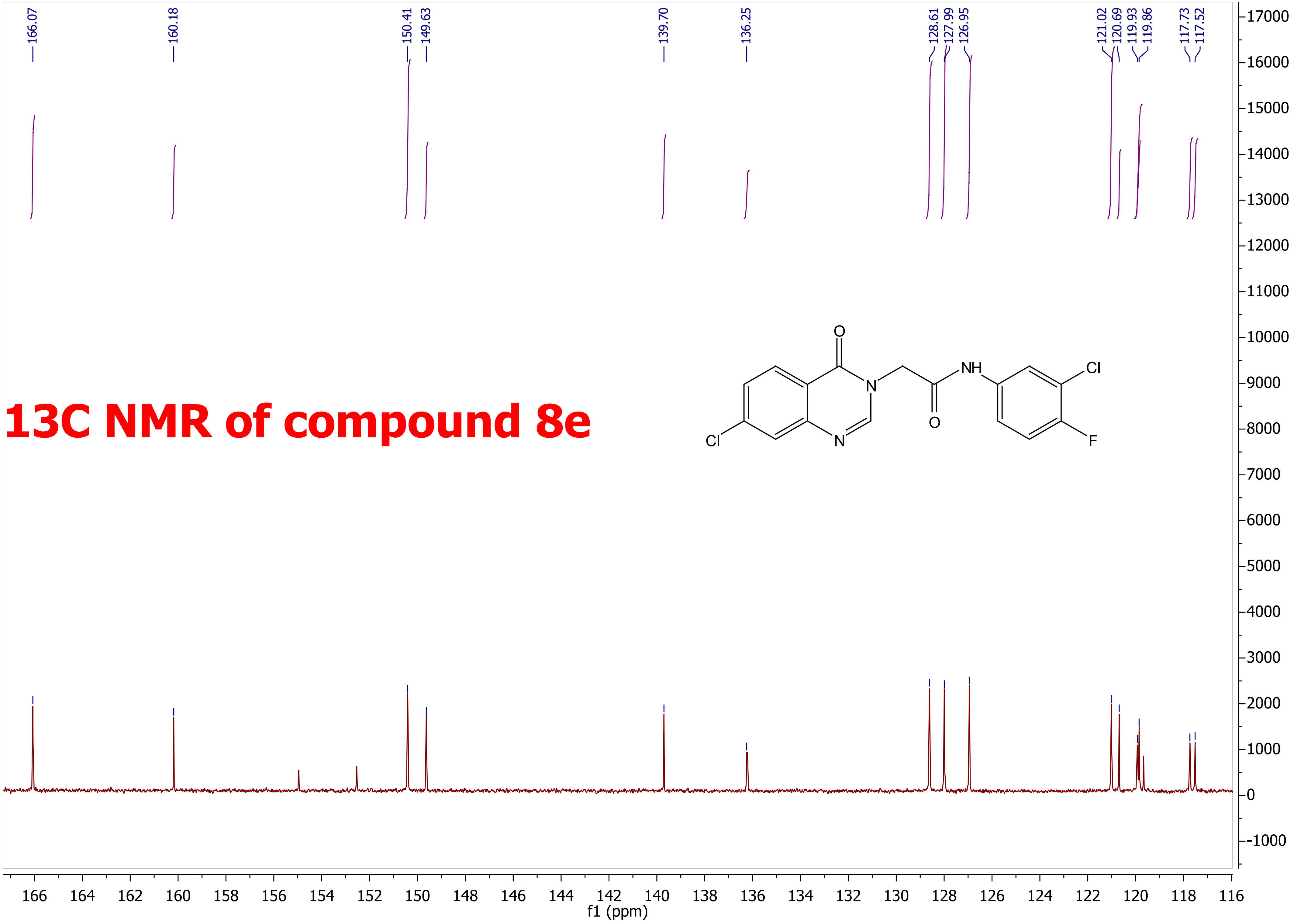
13C NMR of compound 8e
166.07
160.18
150.41
149.63
139.70
136.25
128.61
127.99
126.95
121.02
120.69
119.93
119.86
117.73
117.52
O
N
NH
Cl
Cl
N
O
F
f1 (ppm)
166 164 162 160 158 156 154 152 150 148 146 144 142 140 138 136 134 132 130 128 126 124 122 120 118 116
17000 16000 15000 14000 13000 12000 11000 10000 9000 8000 7000 6000 5000 4000 3000 2000 1000 0 -1000

IR of compound 9a

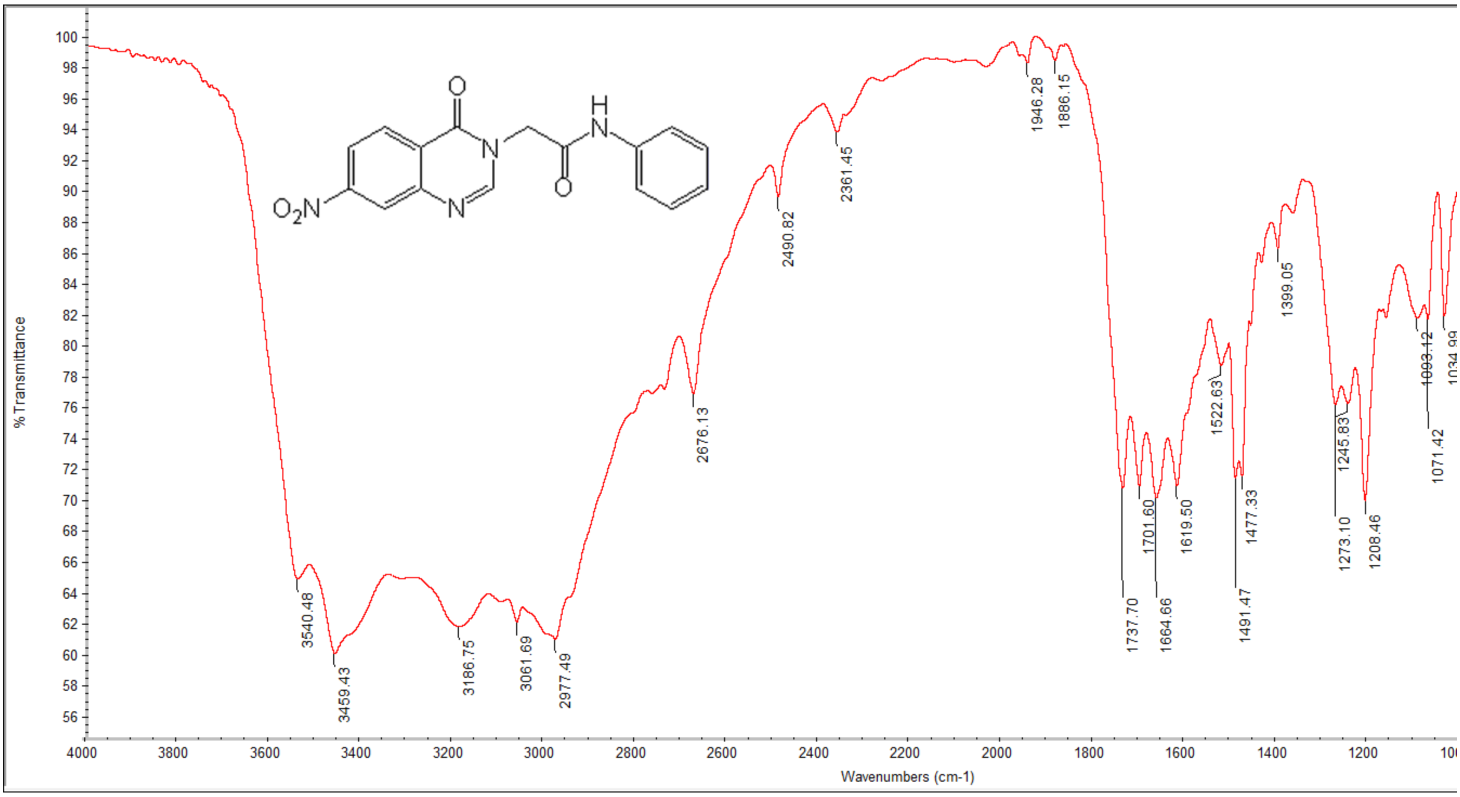

1H NMR of compound 9a

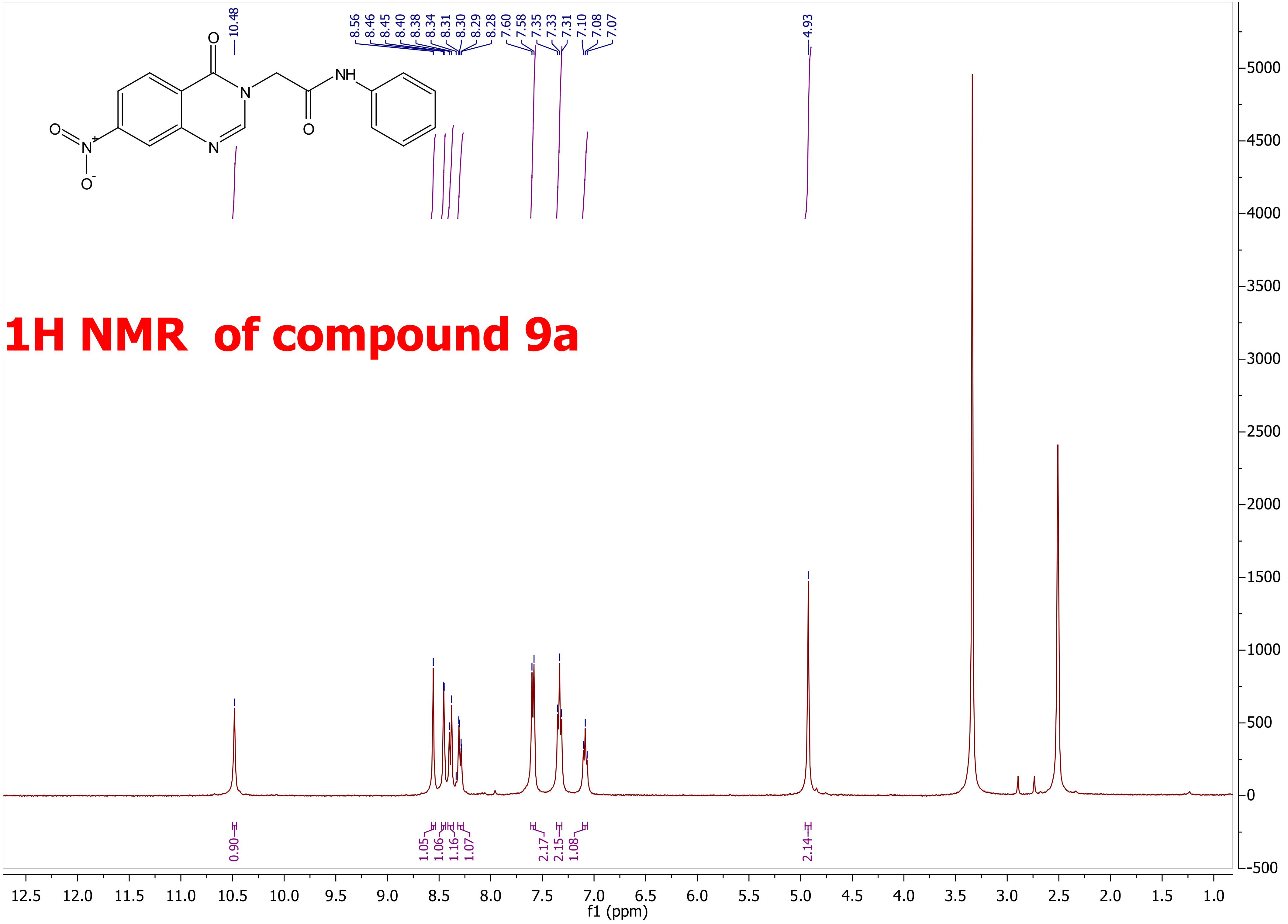

1H NMR of compound 9a

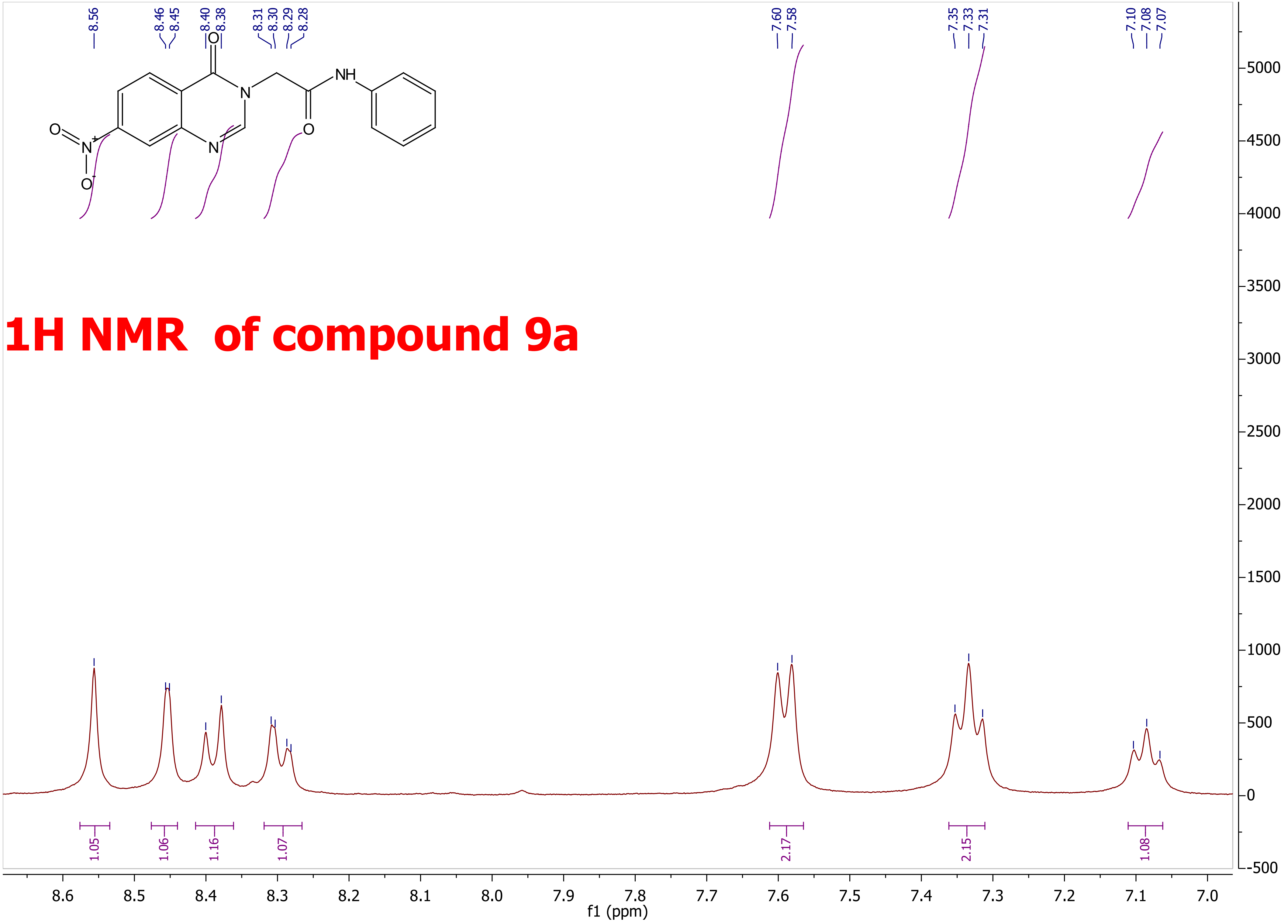

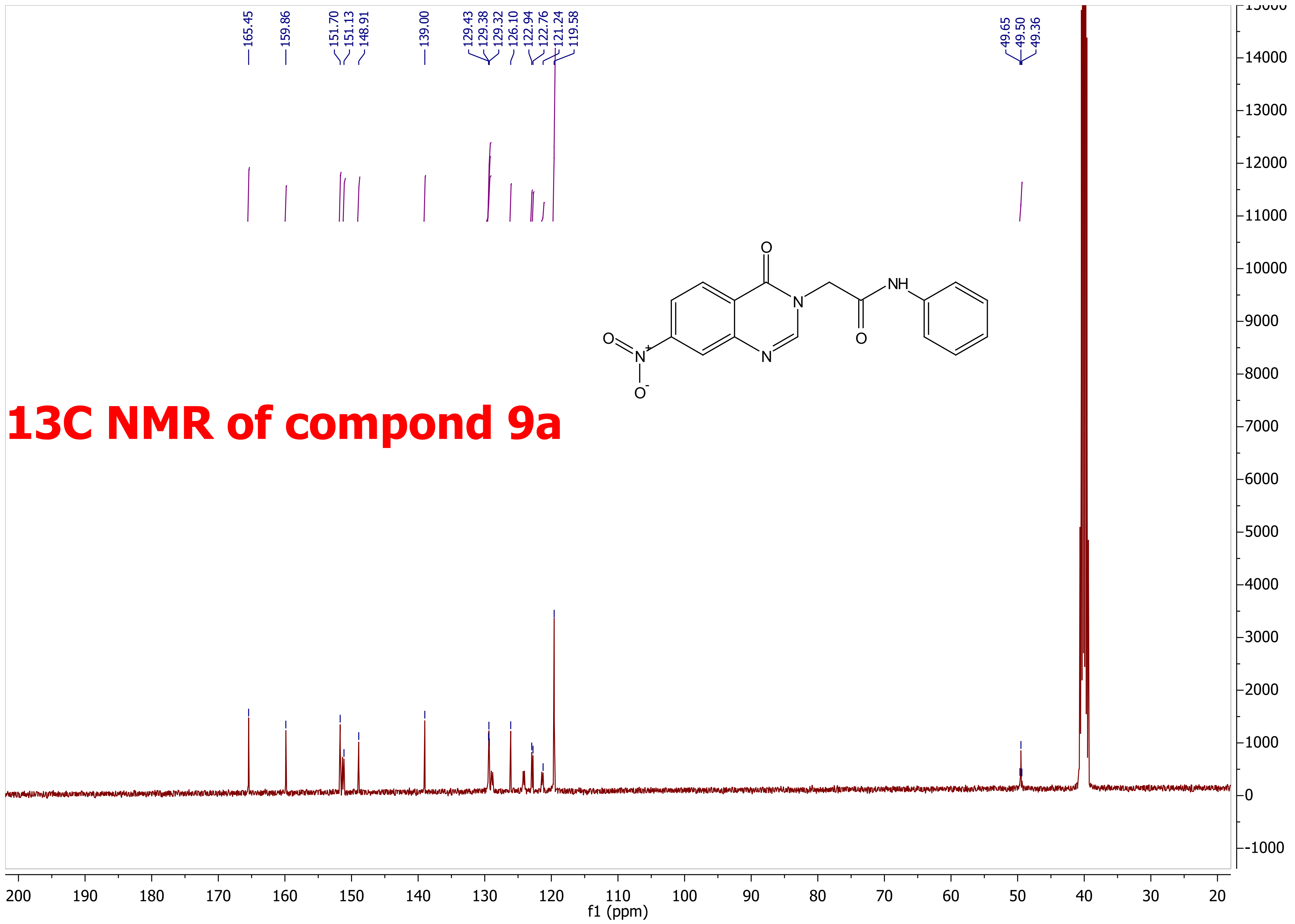
165.45
159.86
151.70
151.13
148.91
139.00
129.43
129.38
129.32
126.10
122.94
122.76
121.24
119.58
49.65
49.50
49.36
13C NMR of compond 9a
f1 (ppm)

13C NMR of compond 9a

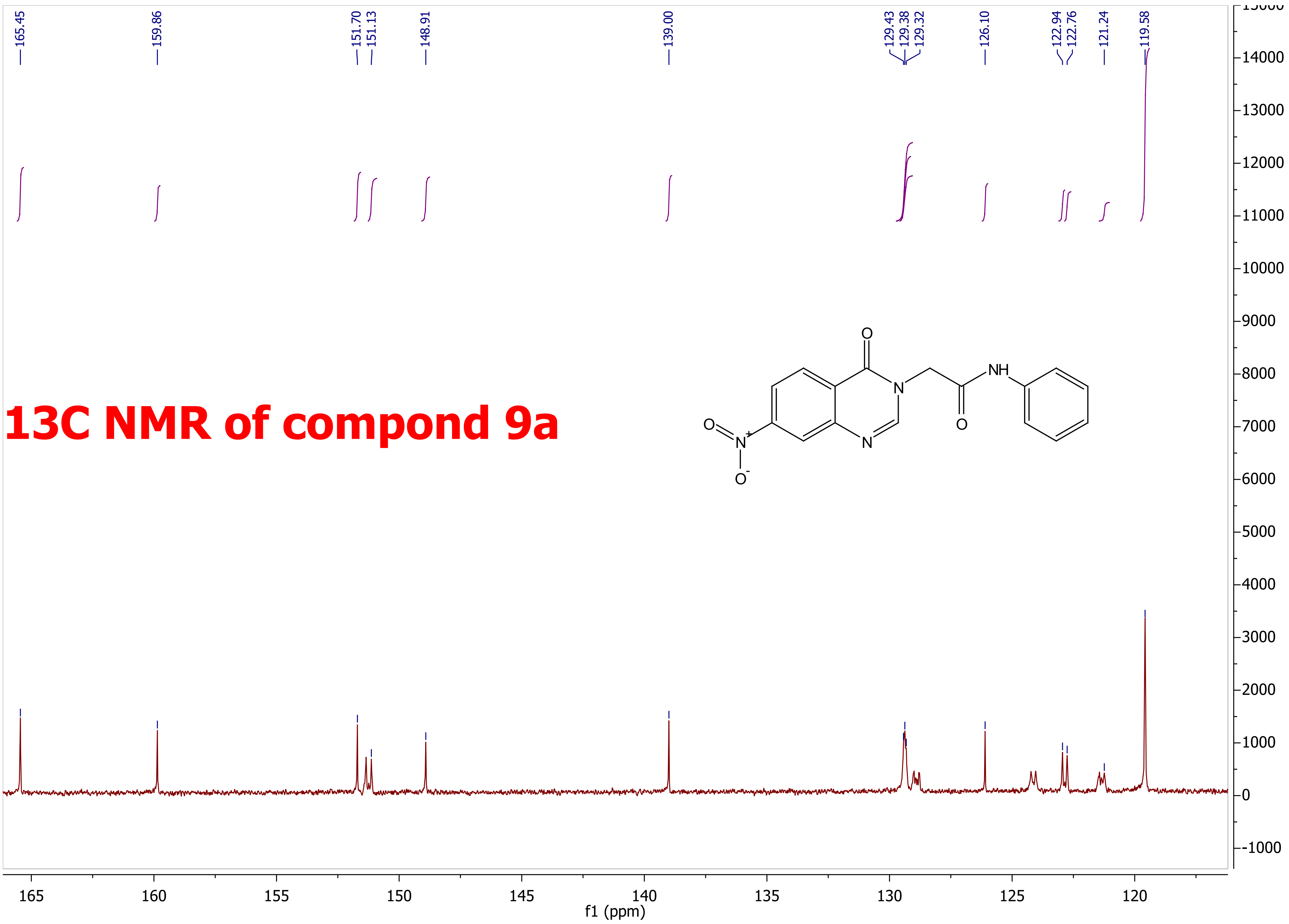

IR of compound 9b

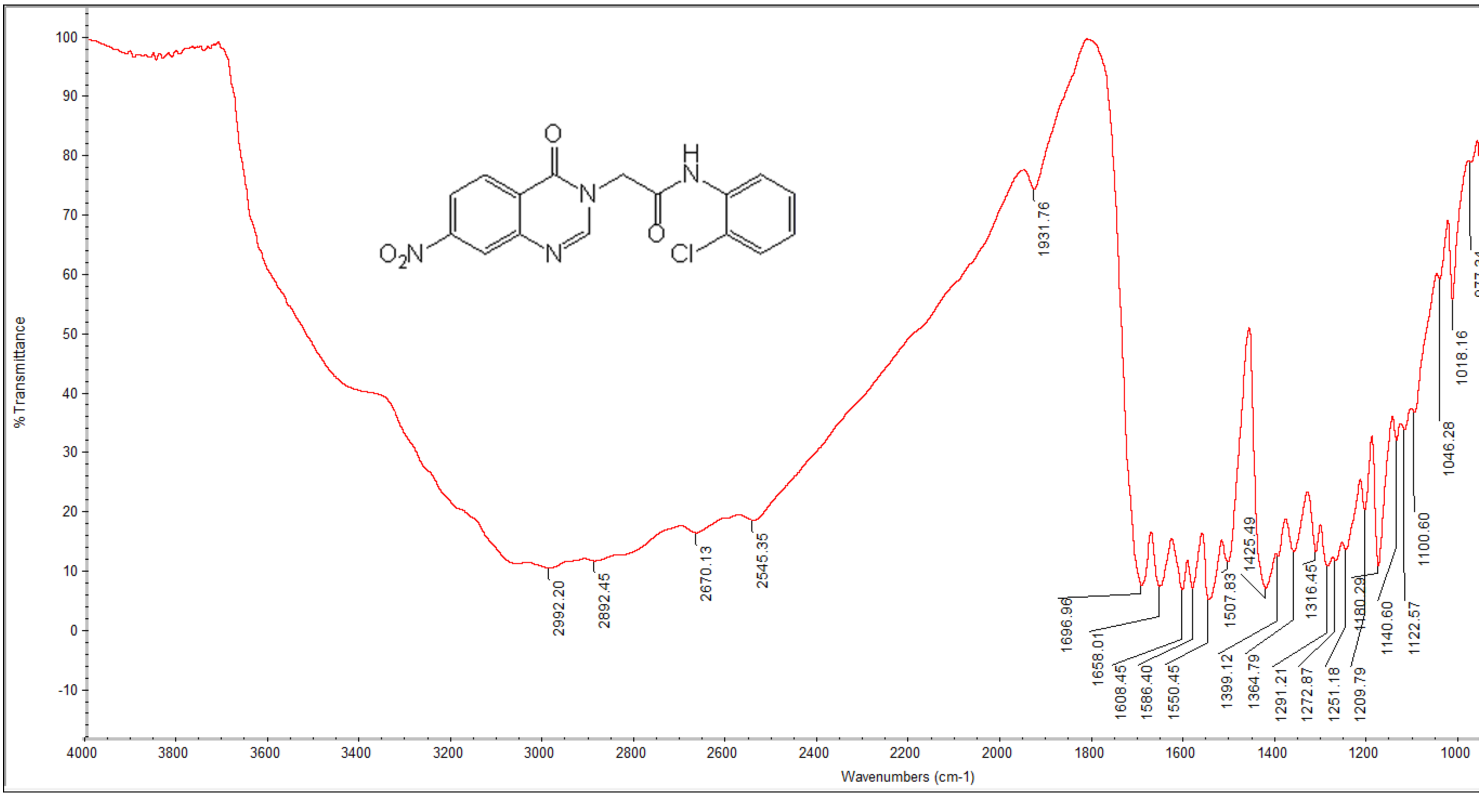

1H NMR of compound 9b

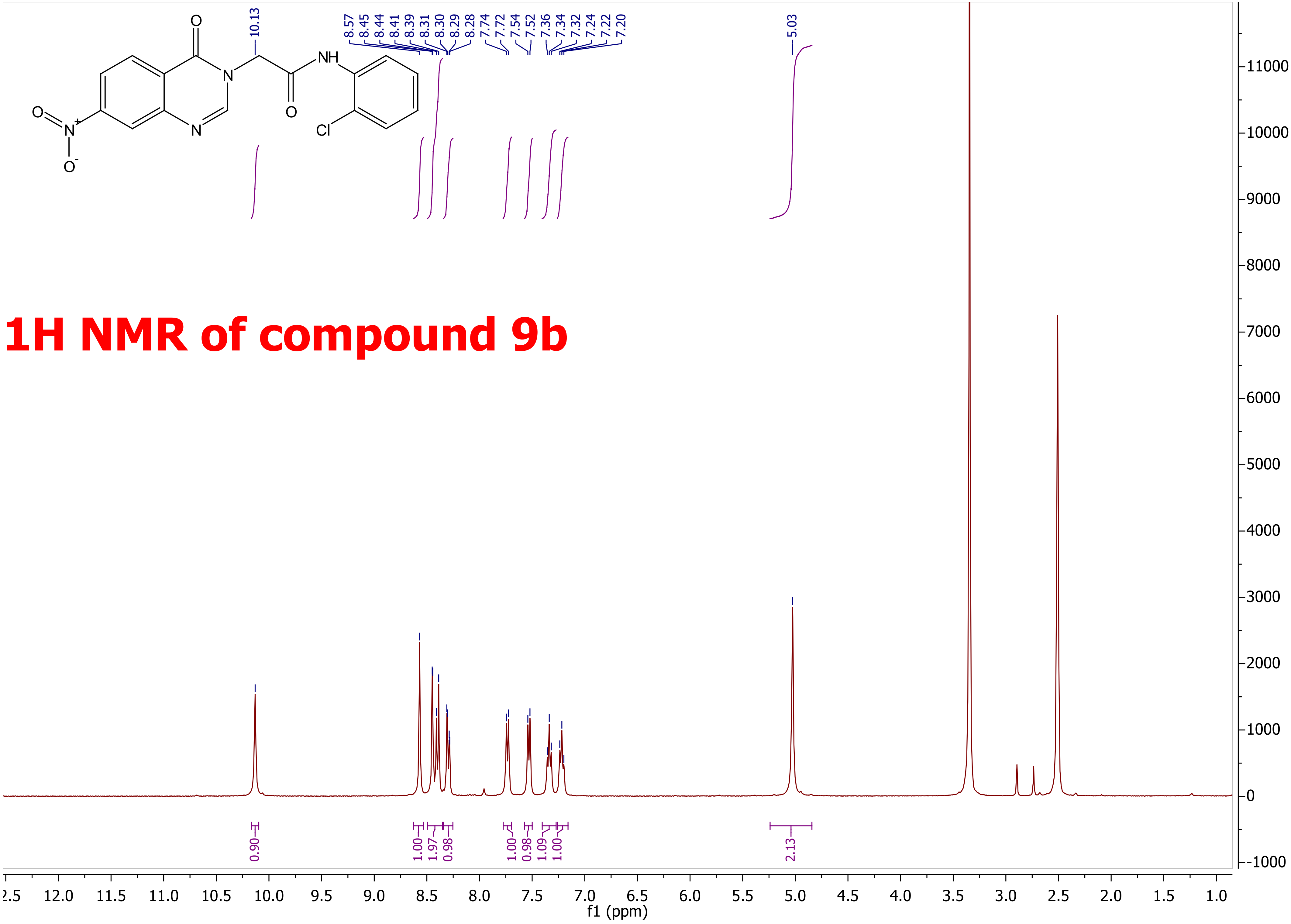

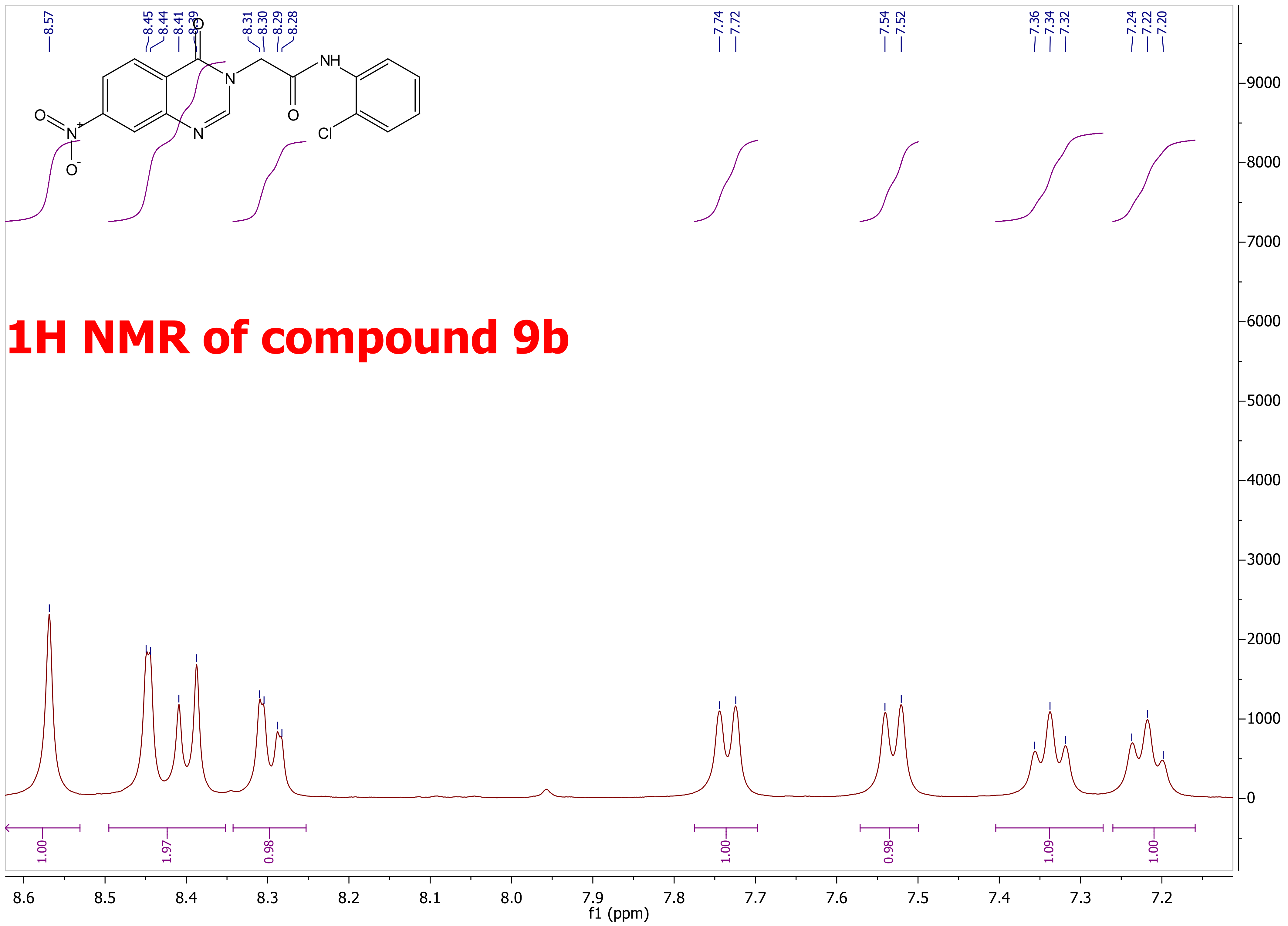
1H NMR of compound 9b
8.57
8.45
8.44
8.41
8.39
8.31
8.30
8.29
8.28
7.74
7.72
7.54
7.52
7.36
7.34
7.32
7.24
7.22
7.20
1.00
1.97
0.98
1.00
0.98
1.09
1.00
f1 (ppm)
O
N
NH
O
N
Cl
O
N+
O-

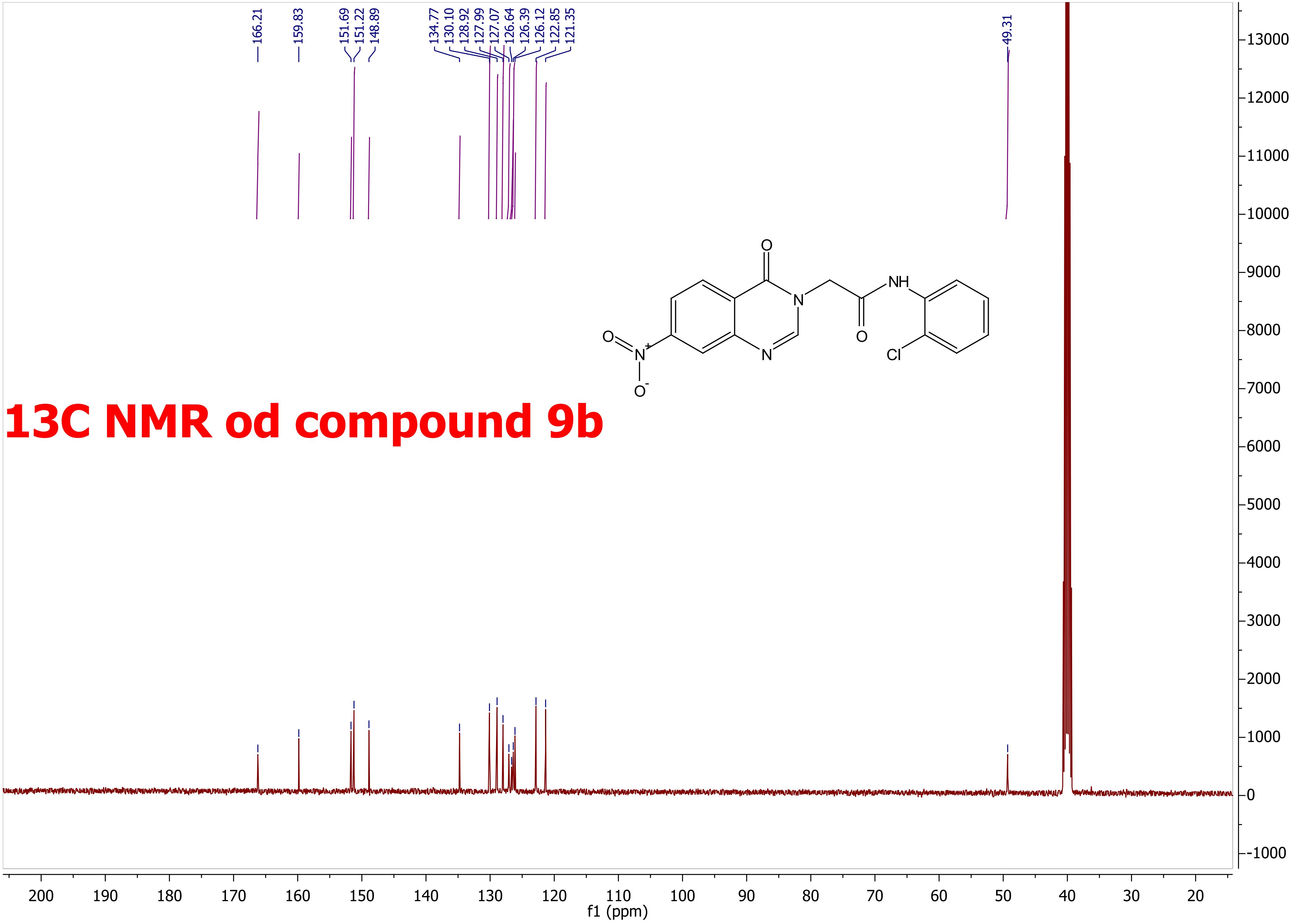
13C NMR od compound 9b
166.21
159.83
151.69
151.22
148.89
134.77
130.10
128.92
127.99
127.07
126.64
126.39
126.12
122.85
121.35
49.31
O
N
NH
O
Cl
N
O
N+
O-
f1 (ppm)
200 190 180 170 160 150 140 130 120 110 100 90 80 70 60 50 40 30 20
13000 12000 11000 10000 9000 8000 7000 6000 5000 4000 3000 2000 1000 0 -1000

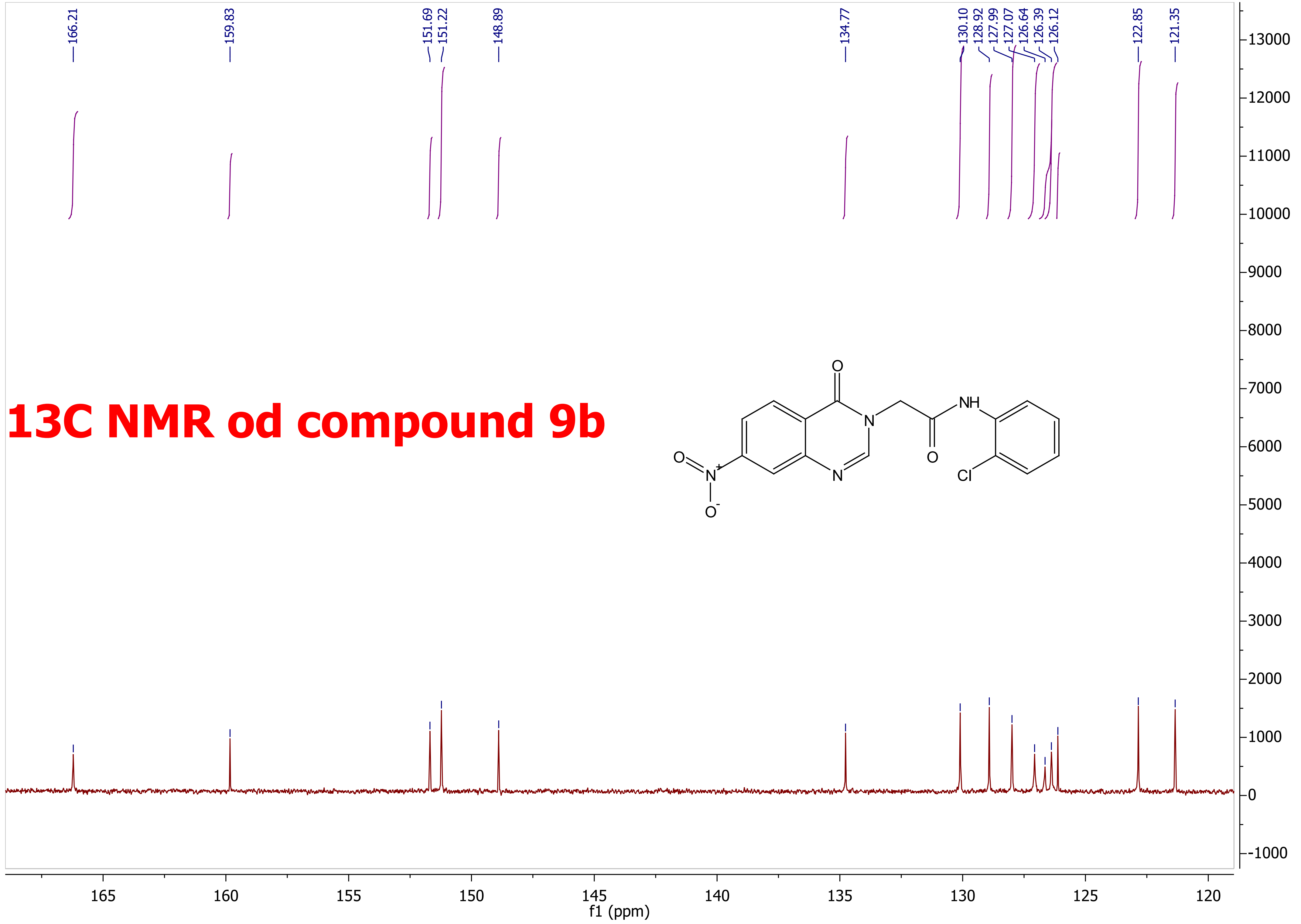
13C NMR od compound 9b
166.21
159.83
151.69
151.22
148.89
134.77
130.10
128.92
127.99
127.07
126.64
126.39
126.12
122.85
121.35
f1 (ppm)
O
N
NH
O
N
O
N+
O-
Cl

IR of compound 9c

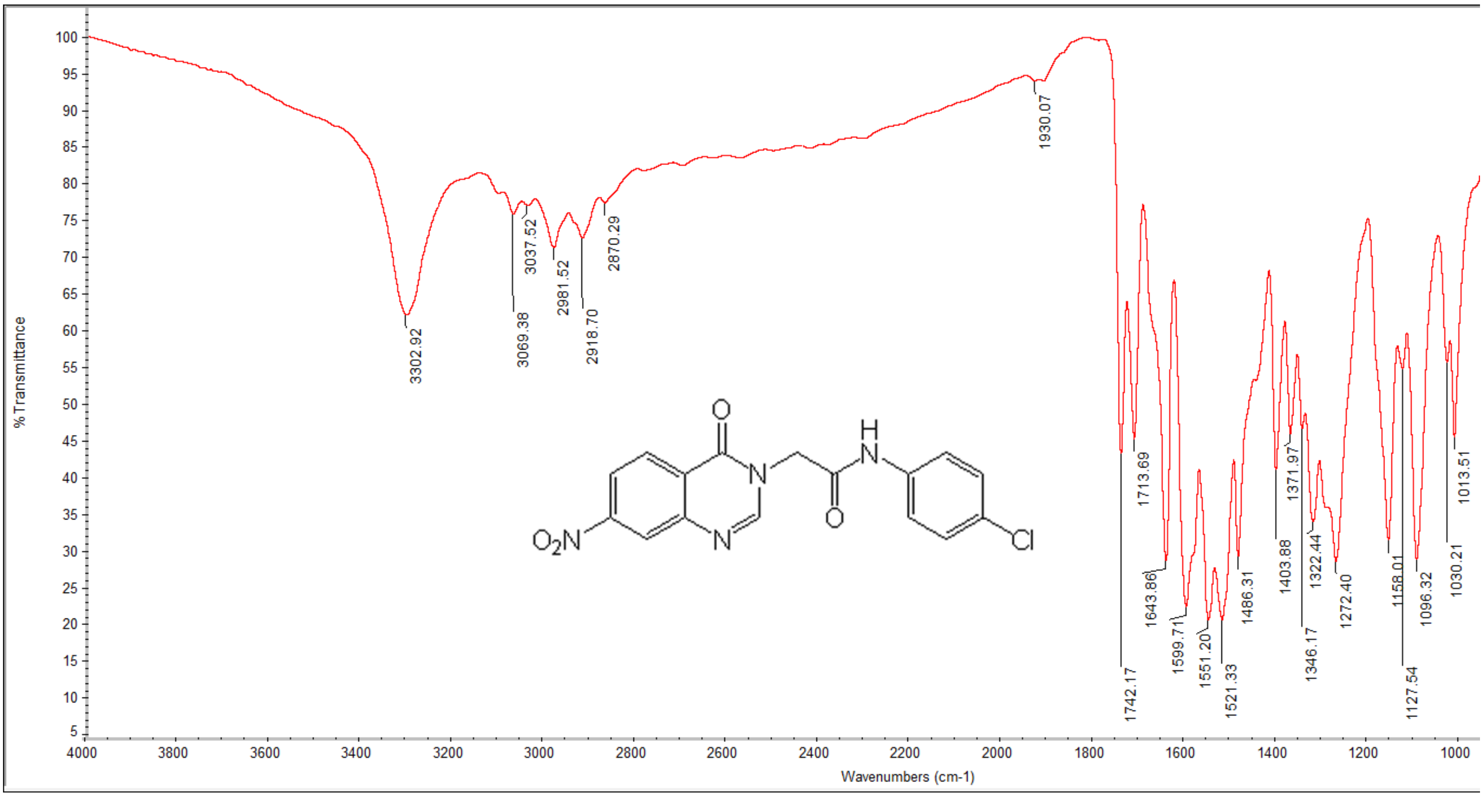

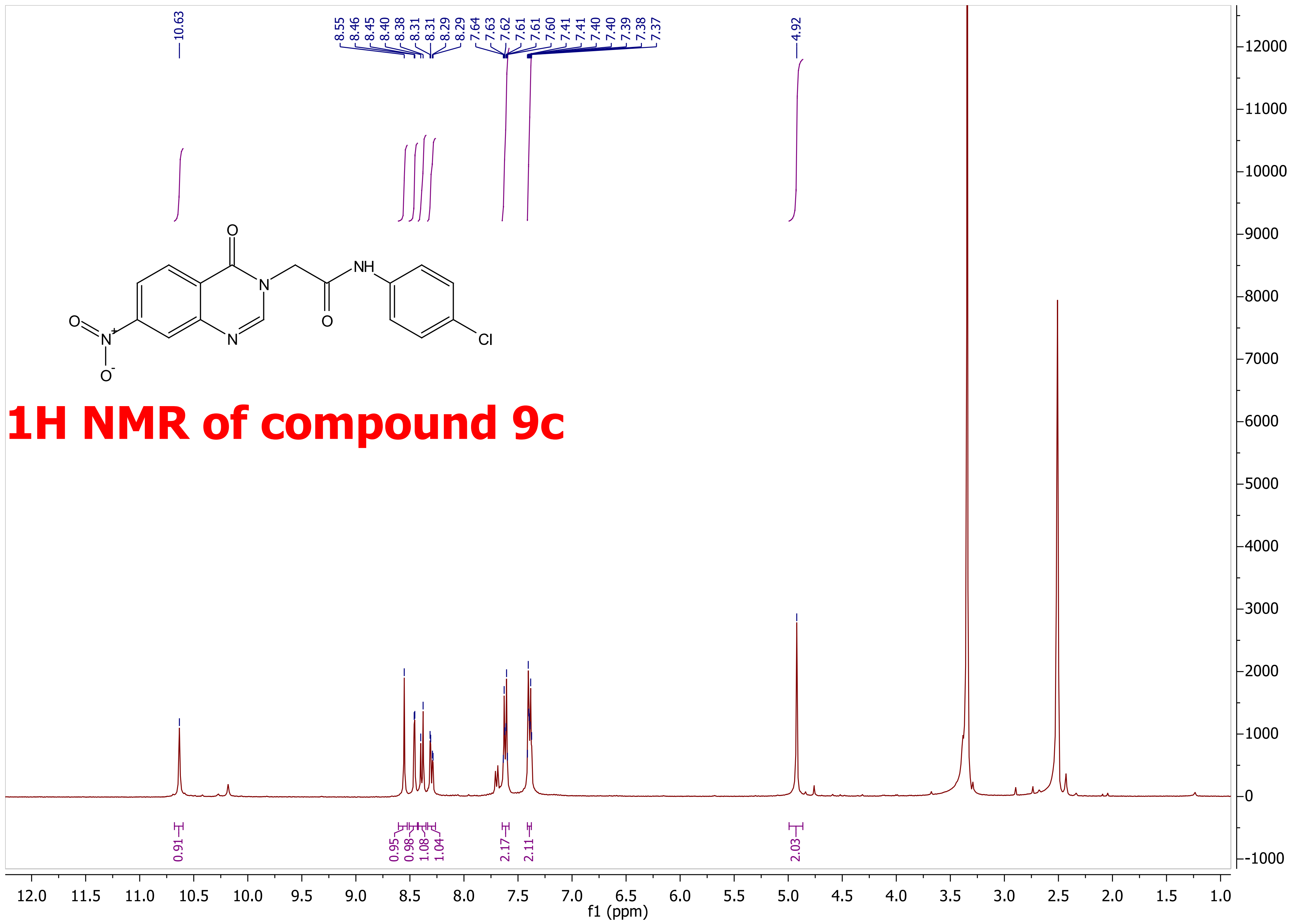
1H NMR of compound 9c
10.63
8.55
8.46
8.45
8.40
8.38
8.31
8.31
8.29
8.29
7.64
7.63
7.62
7.61
7.61
7.60
7.41
7.41
7.40
7.40
7.39
7.38
7.37
4.92
0.91
0.95
0.98
1.08
1.04
2.17
2.11
2.03
f1 (ppm)
NH
Cl
N
O

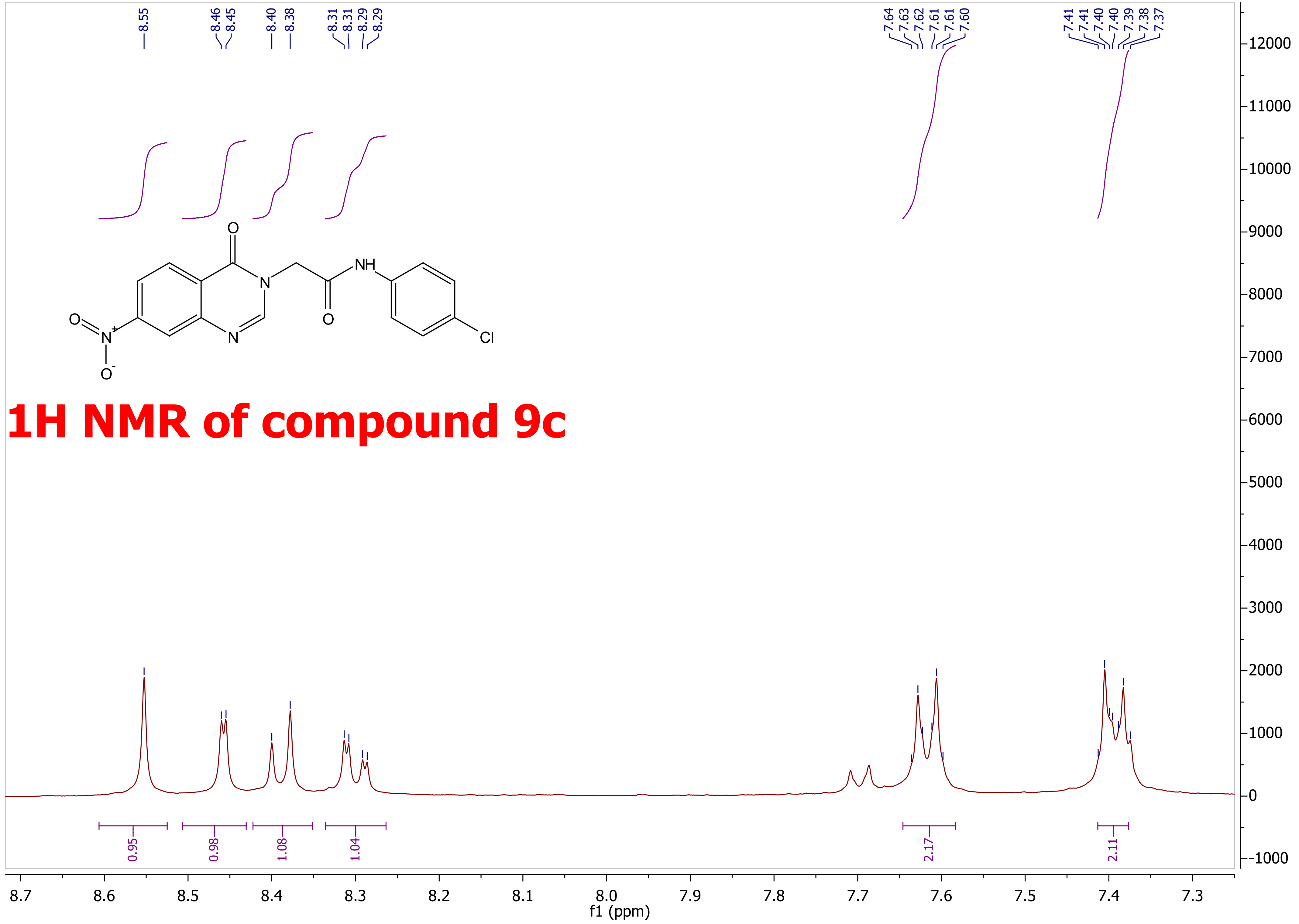
8.55
8.46
8.45
8.40
8.38
8.31
8.31
8.29
8.29
7.64
7.63
7.62
7.61
7.61
7.60
7.41
7.41
7.40
7.40
7.39
7.38
7.37
O
N
NH
O
O
N+
O-
N
Cl
1H NMR of compound 9c
0.95
0.98
1.08
1.04
2.17
2.11
8.7
8.6
8.5
8.4
8.3
8.2
8.1
8.0
7.9
7.8
7.7
7.6
7.5
7.4
7.3
f1 (ppm)
12000
11000
10000
9000
8000
7000
6000
5000
4000
3000
2000
1000
0
-1000

13C NMR of compound 9c

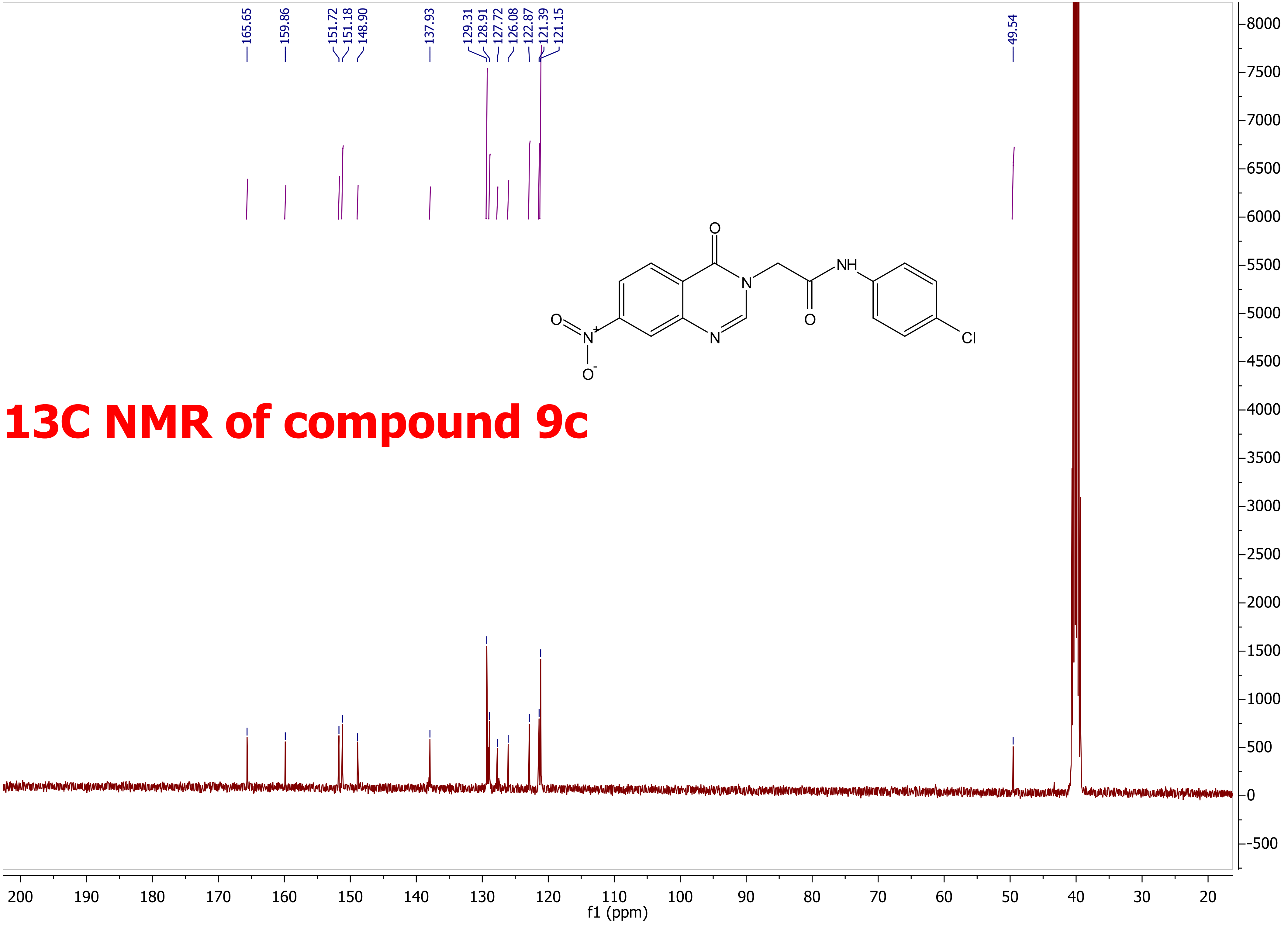

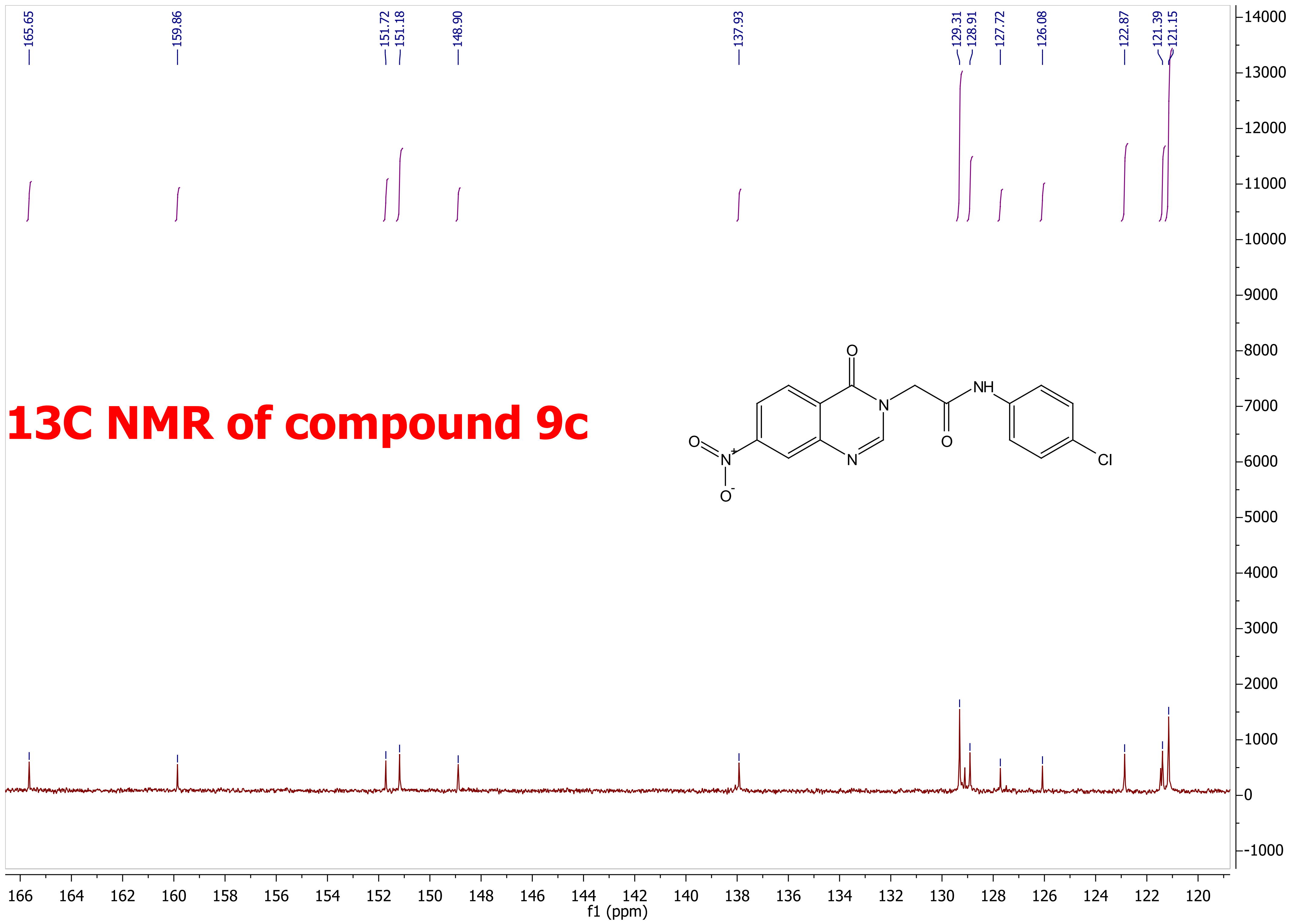
165.65
159.86
151.72
151.18
148.90
137.93
129.31
128.91
127.72
126.08
122.87
121.39
121.15
13C NMR of compound 9c
f1 (ppm)

IR of compound 9d

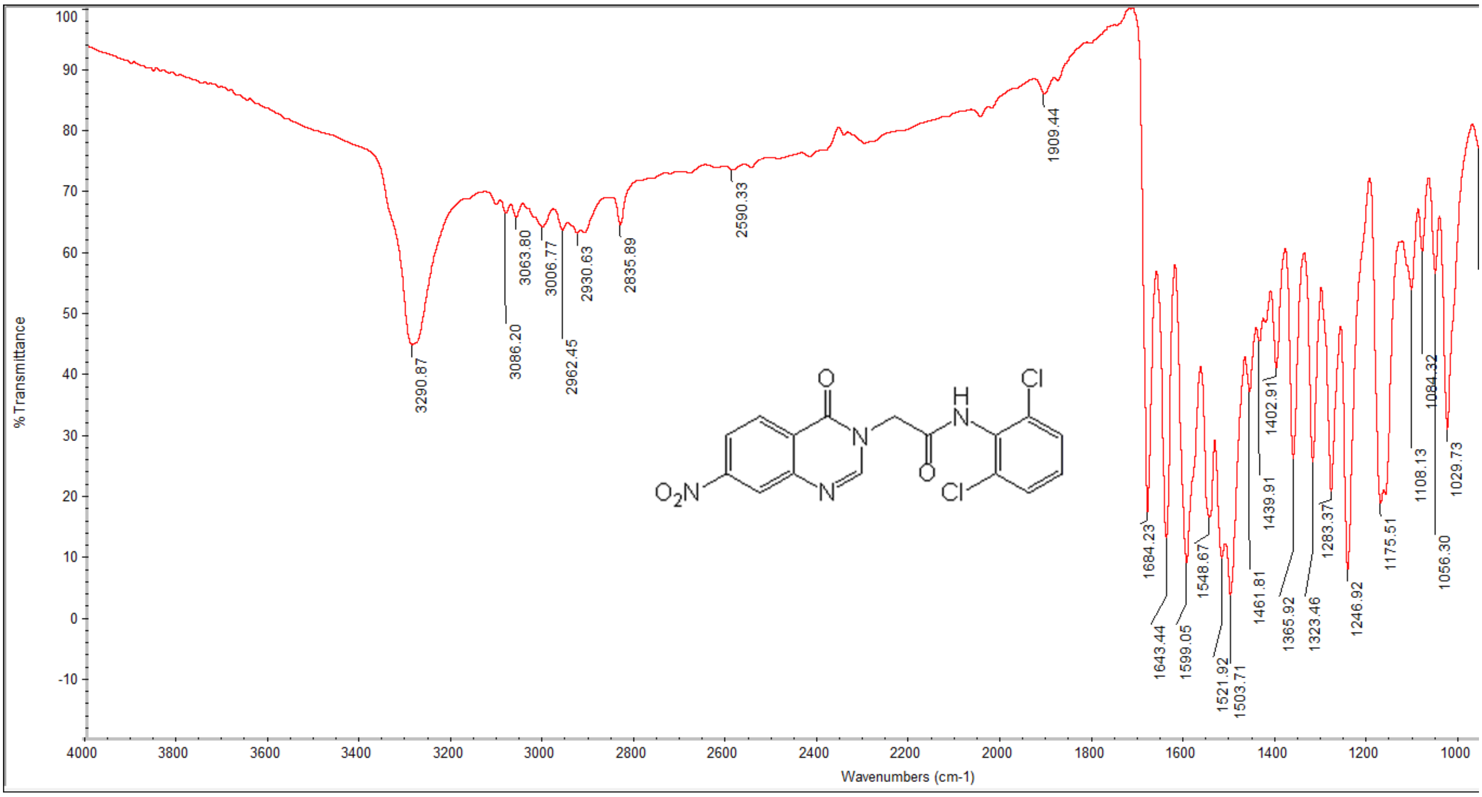

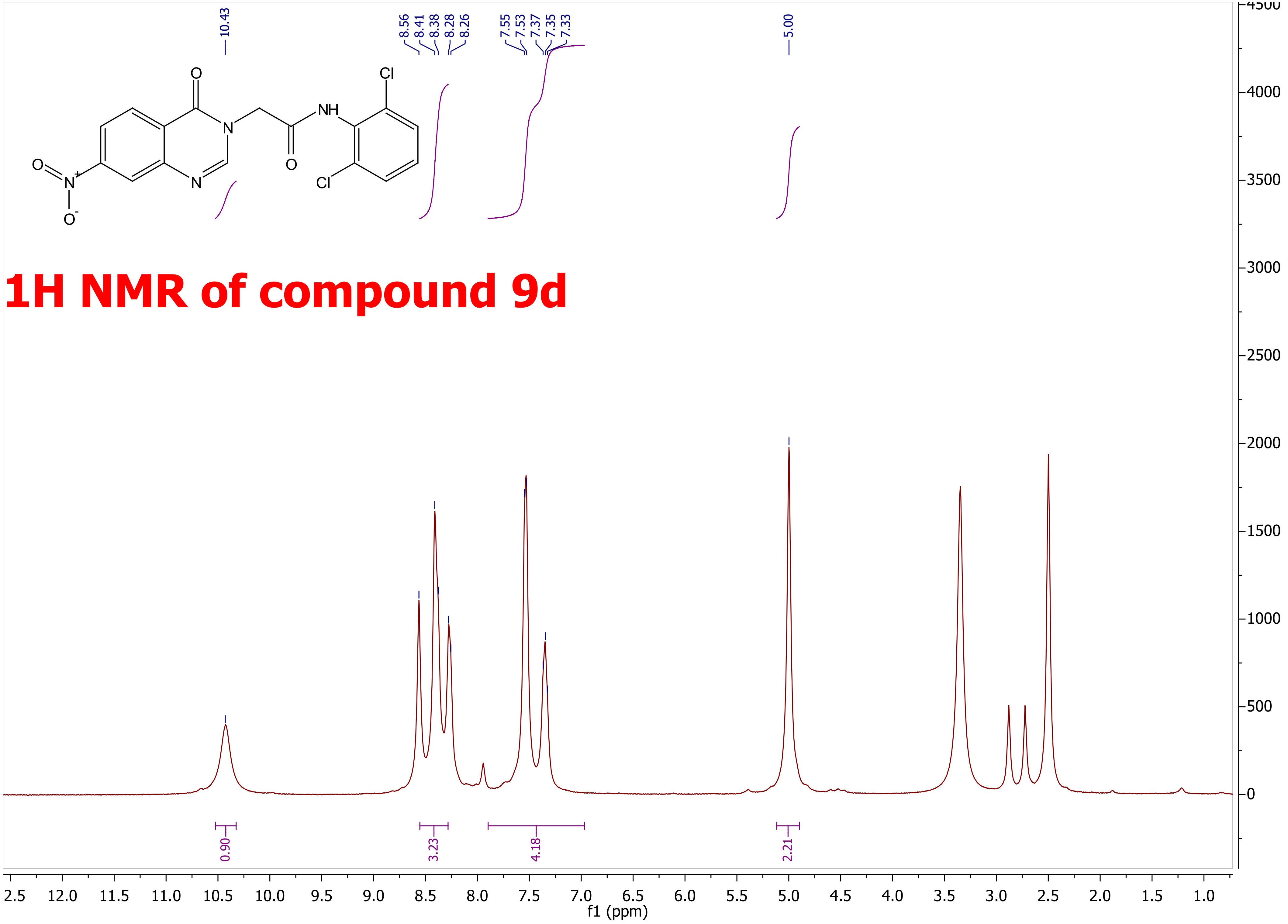

1H NMR of compound 9d

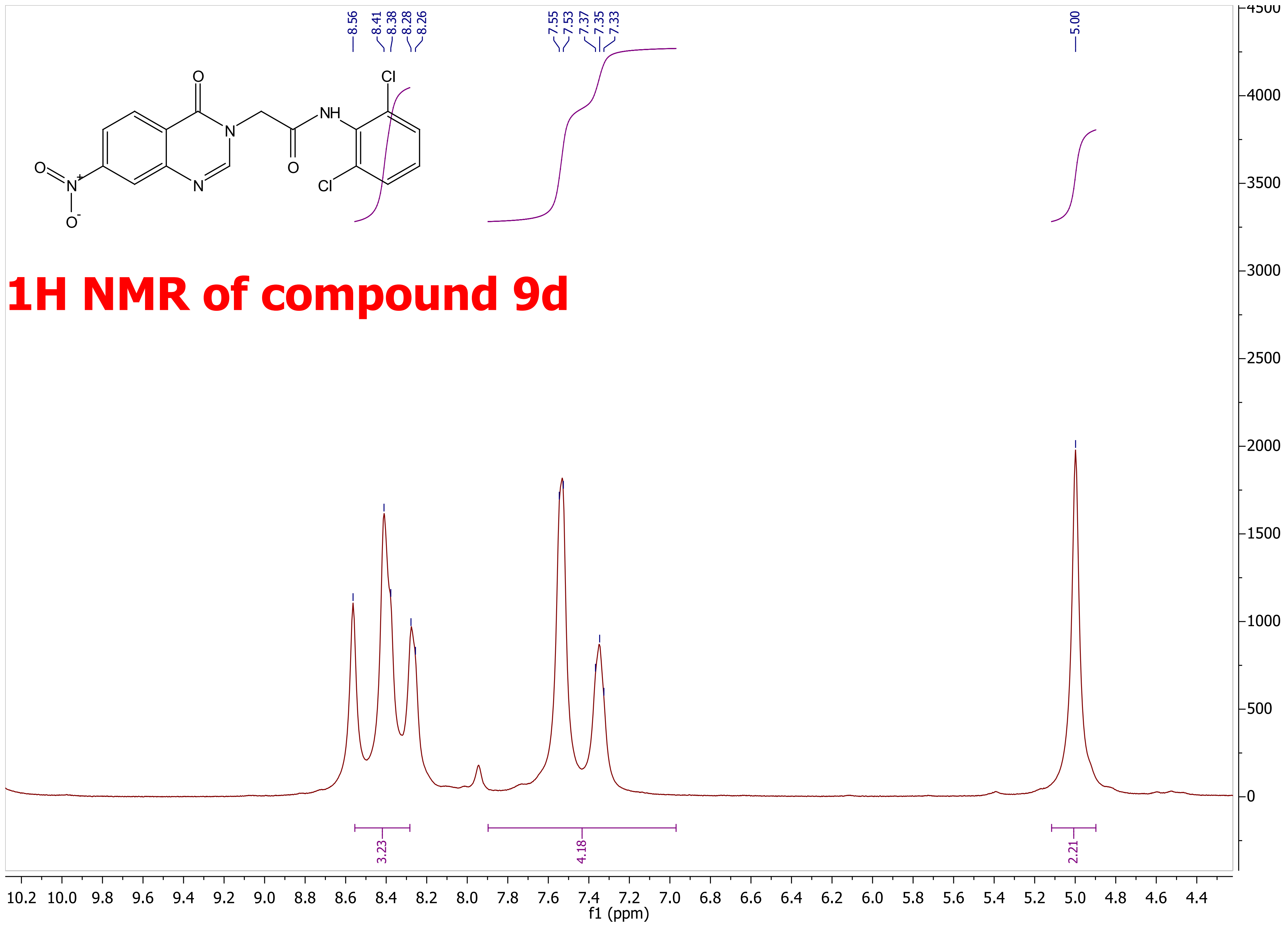
1H NMR of compound 9d
8.56
8.41
8.38
8.28
8.26
7.55
7.53
7.37
7.35
7.33
5.00
3.23
4.18
2.21
f1 (ppm)

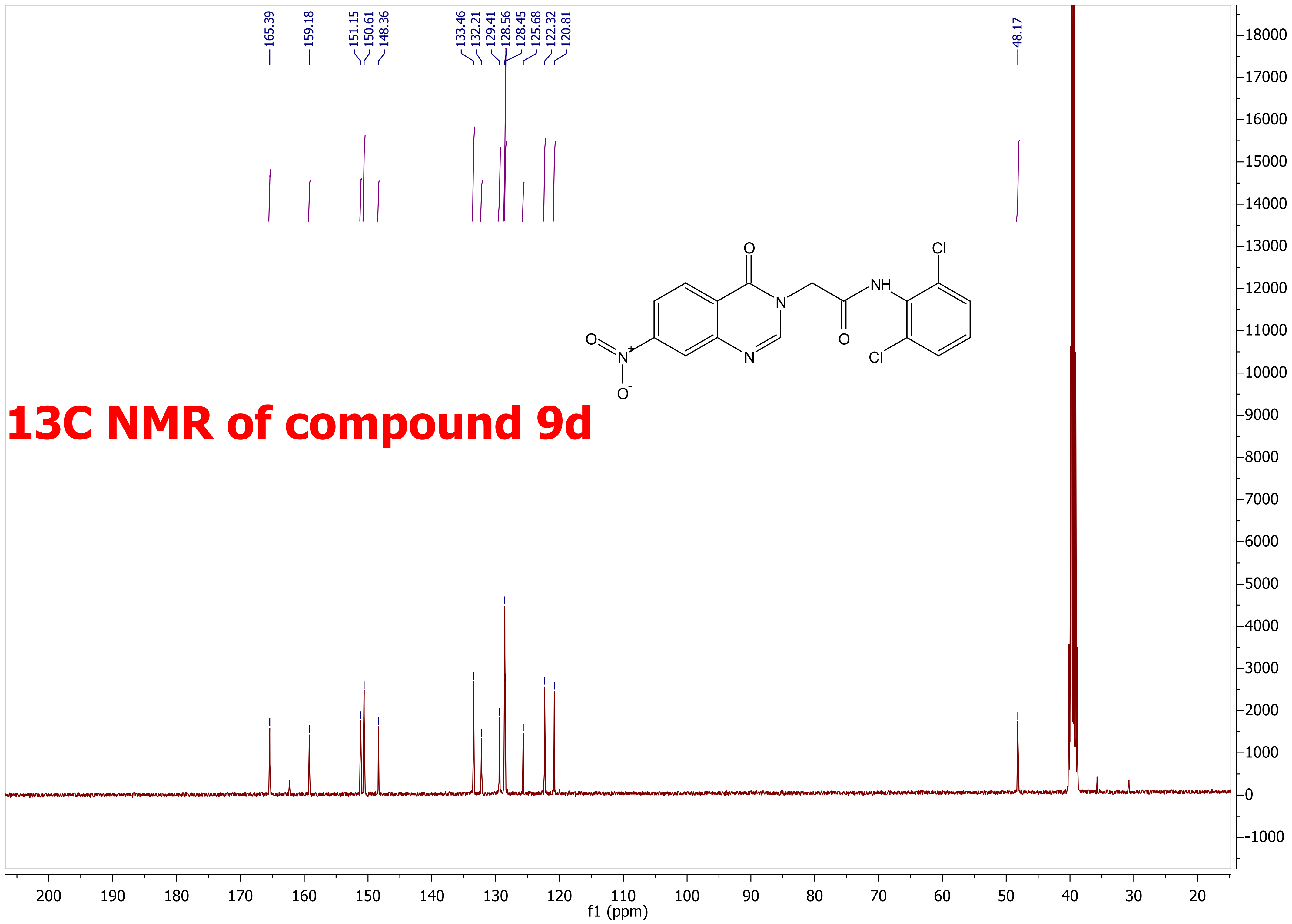
165.39
159.18
151.15
150.61
148.36
133.46
132.21
129.41
128.56
128.45
125.68
122.32
120.81
48.17
13C NMR of compound 9d
f1 (ppm)

13C NMR of compound 9d

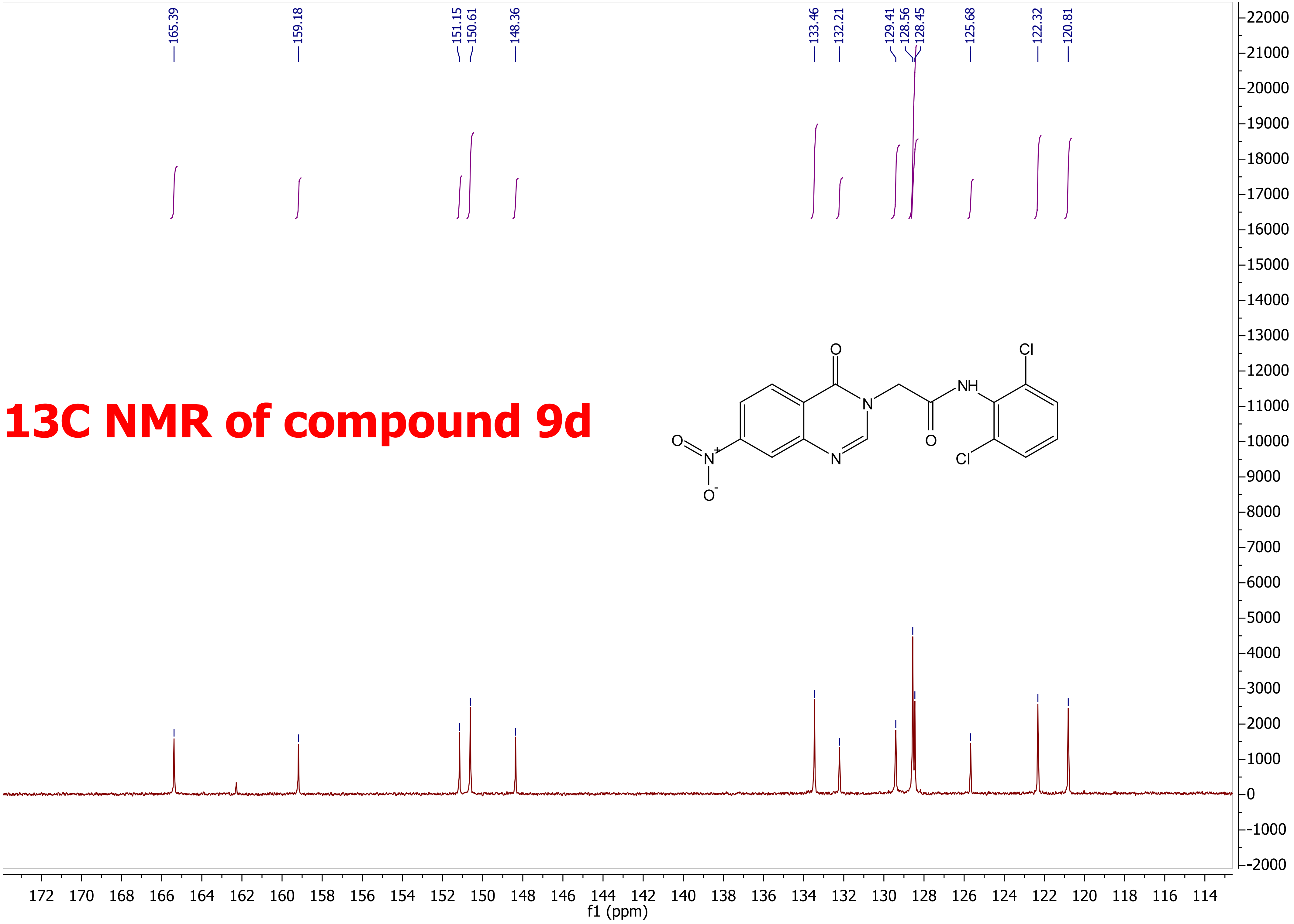

IR of compound 9e

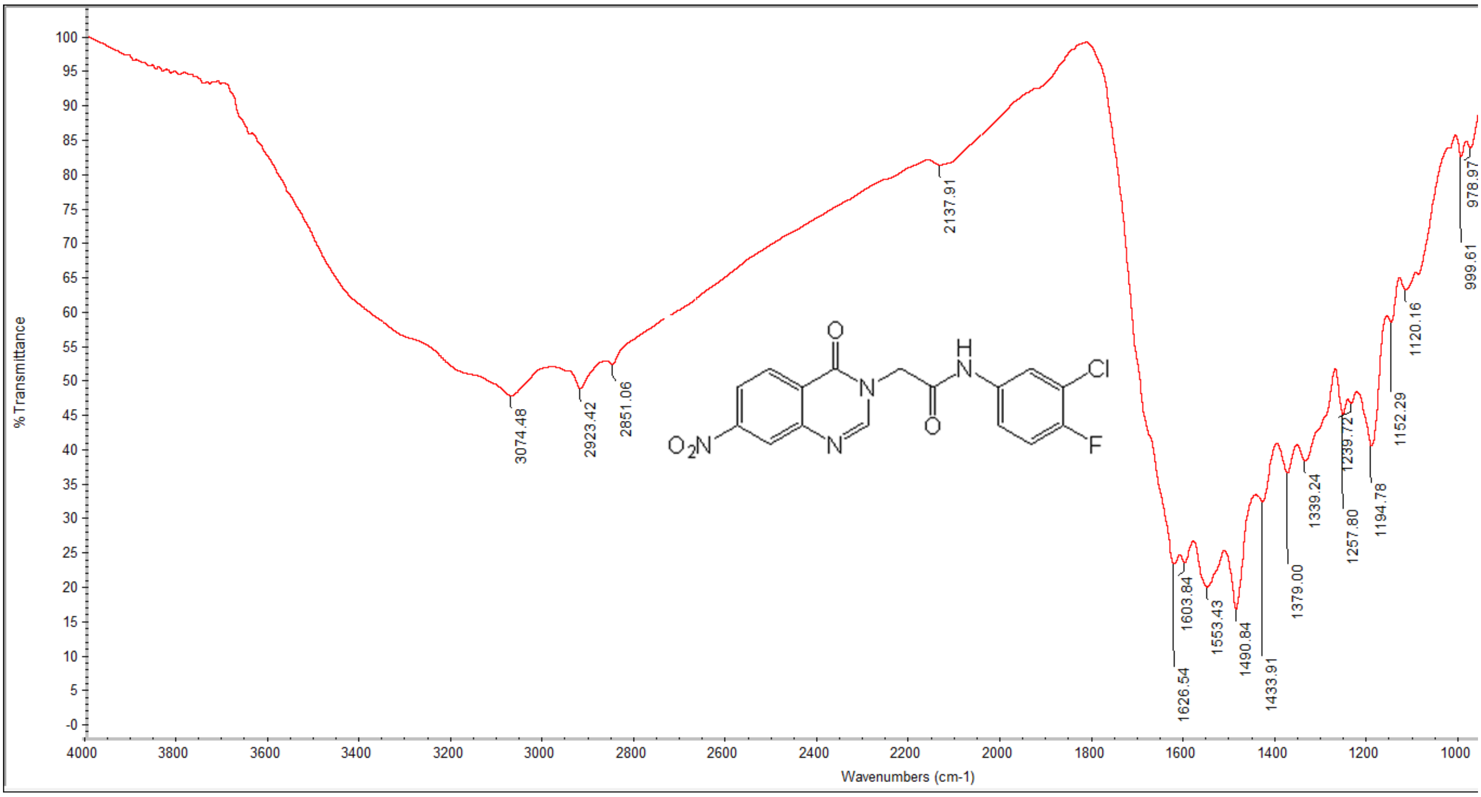

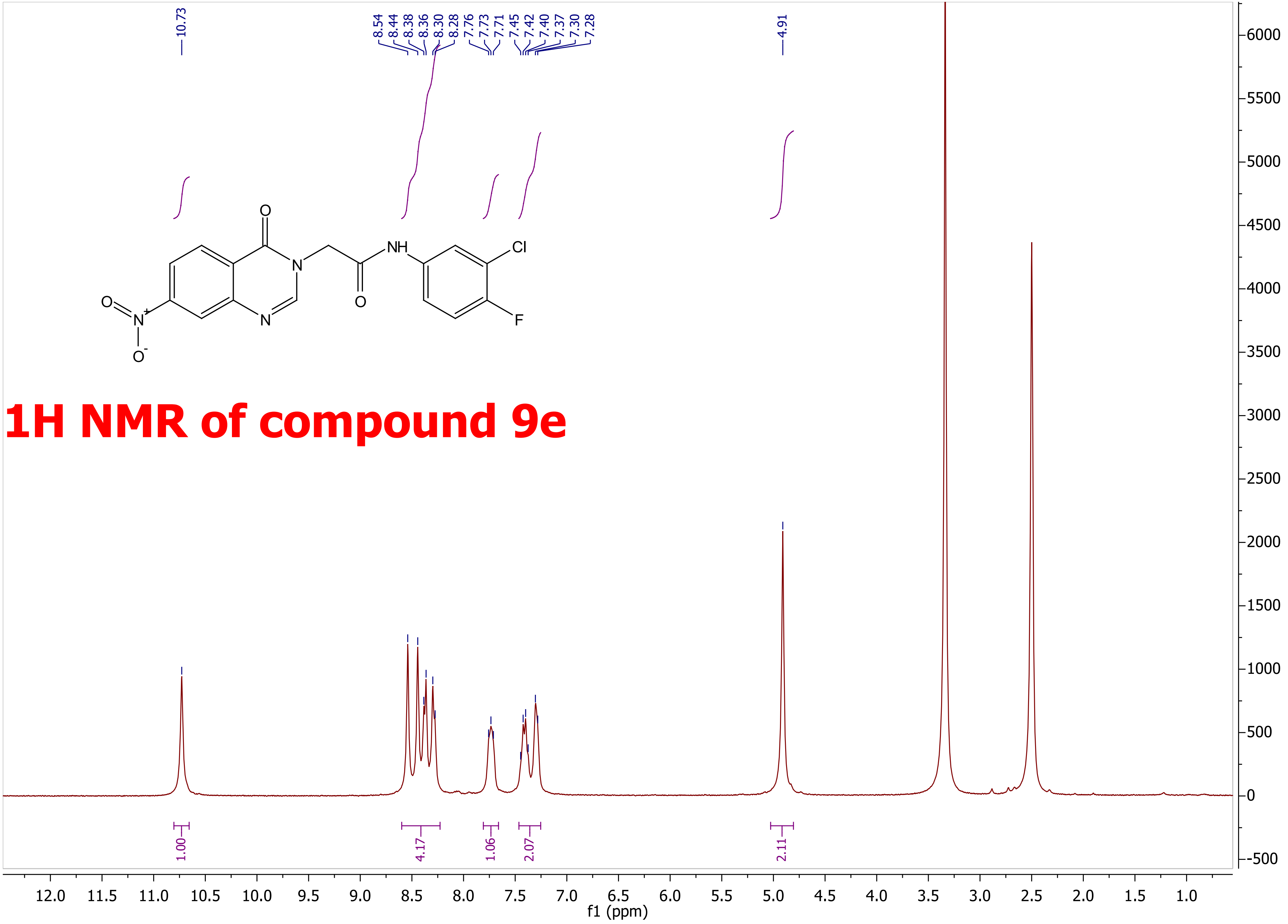
1H NMR of compound 9e
10.73
8.54
8.44
8.38
8.36
8.30
8.28
7.76
7.73
7.71
7.45
7.42
7.40
7.37
7.30
7.28
4.91
1.00
4.17
1.06
2.07
2.11
O
N
NH
O
Cl
F
N
O
N+
O-
f1 (ppm)

1H NMR of compound 9e

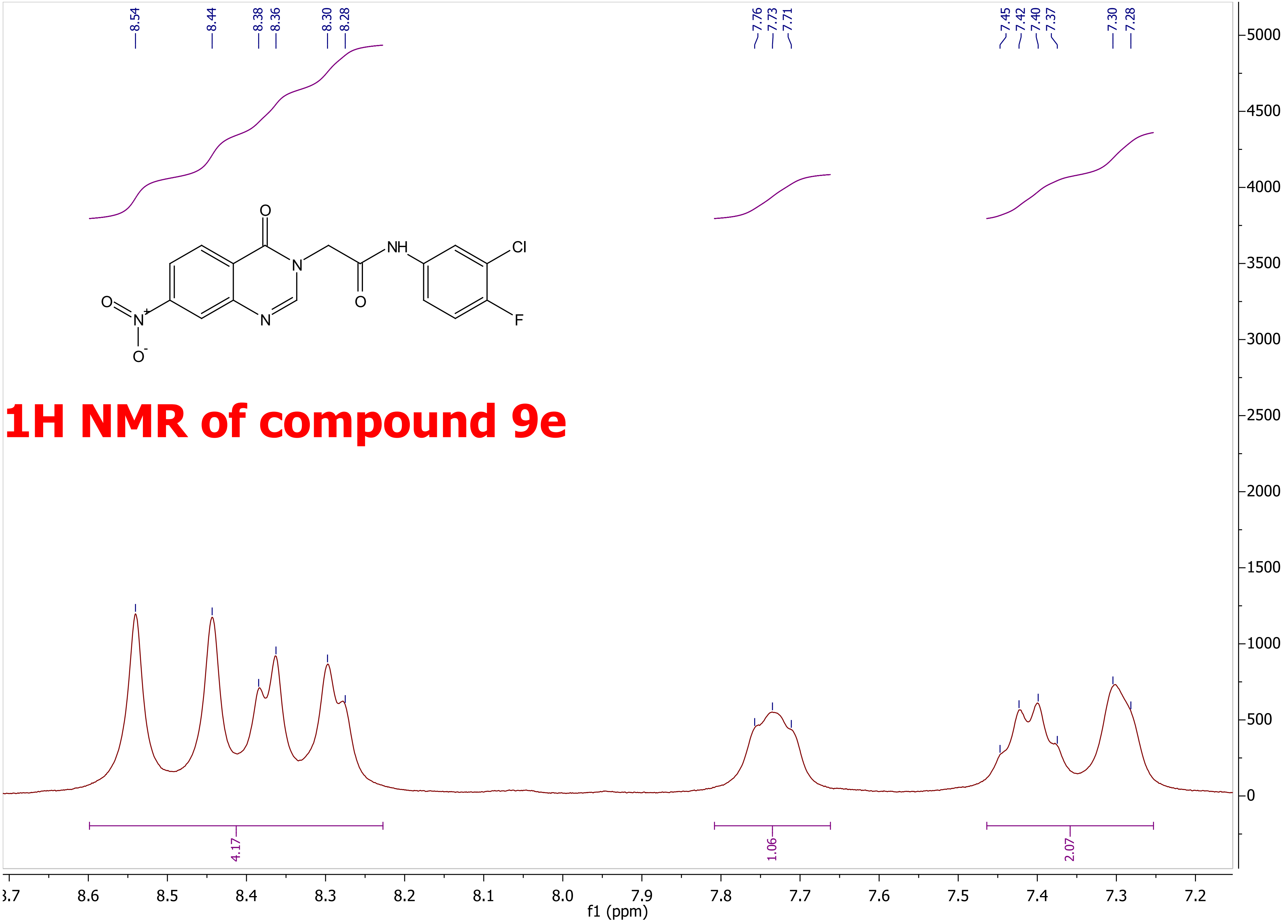

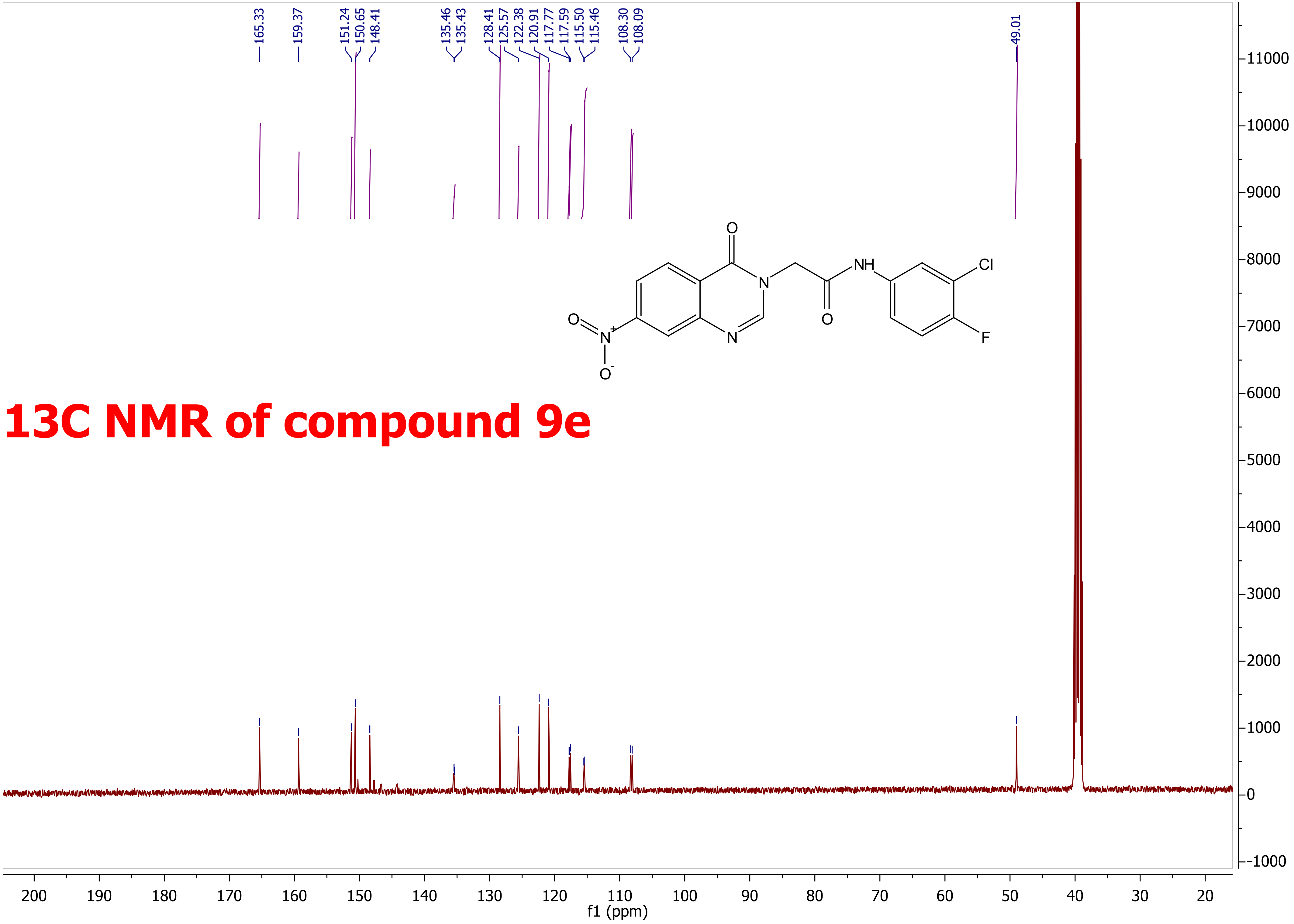

13C NMR of compound 9e

13C NMR of compound 9e

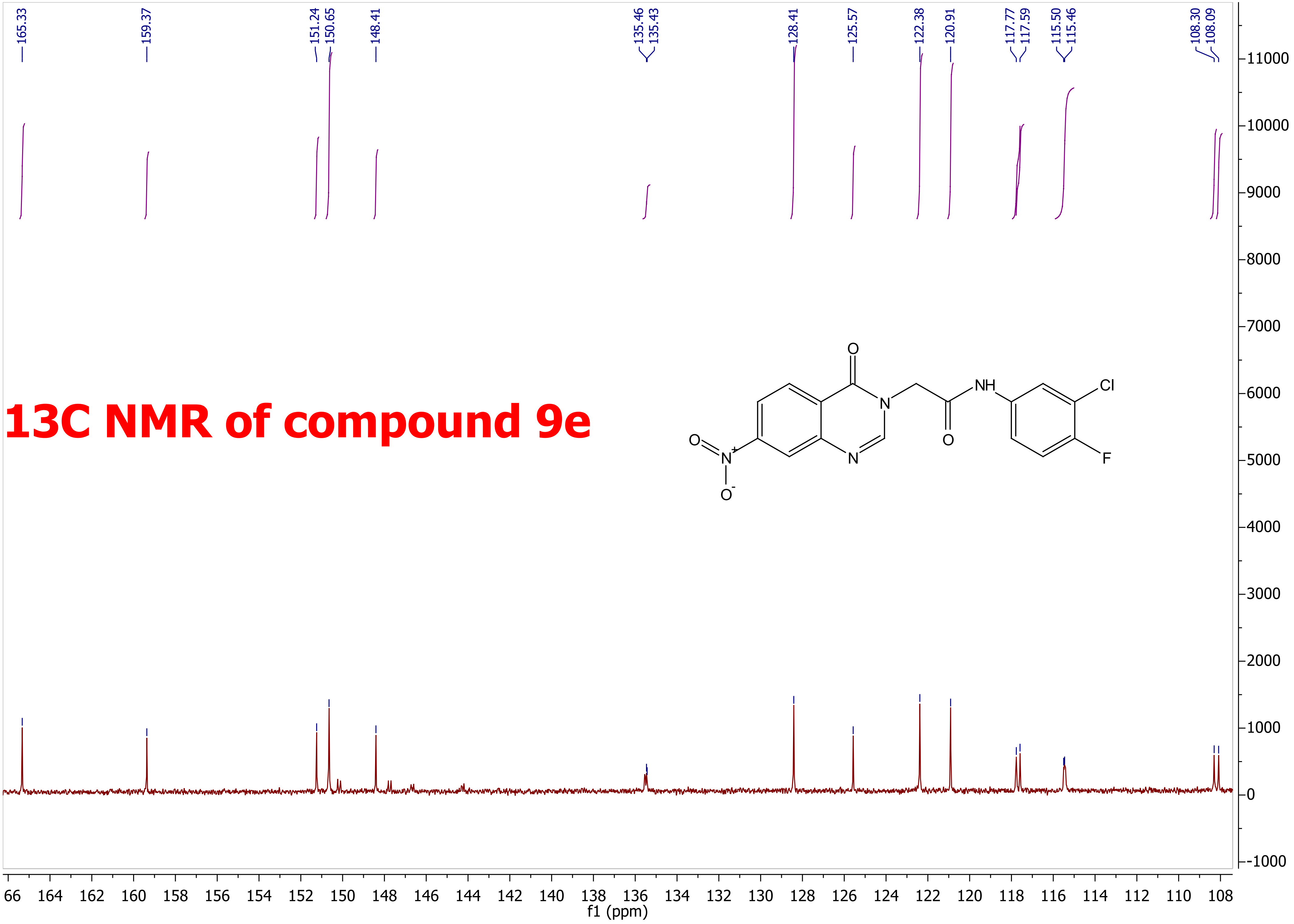

Requester Data:

Name: **Prof.Dr. Mohamed Ayman Alzahaby**
Dr. Reda Rezk

Authority: Faculty of Pharmacy, Al-Azhar University

Sample Data:

Ten samples had been submitted for elemental analysis.

Analysis Report:

Sample Code	C%	H%	N%
N_1	59.37	3.96	17.46
N_2	53.71	3.32	15.89
N_3	49.09	2.70	14.51
N_4	51.27	2.89	15.06
N_5	53.81	3.26	15.84
R_1	55.37	3.40	12.31
R_2	61.13	4.05	13.62
R_3	55.40	3.29	12.25
R_4	50.41	2.89	11.20
R_5	52.67	2.98	11.75

INVESTIGATOR Dr. Mourad

DIRECTOR M. Mansour
10. 11. 2021

جامعة الأزهر
المركز الإقليمى للفطريات وتطبيقاتها

3- Experimental of biological testing

4.2.1. *In vitro* anti-proliferative activity

The *in vitro* antiproliferative activities of the synthesized compounds were evaluated against three human tumor cell lines: (MCF-7, breast cancer, HepG-2, hepatocellular carcinoma, and K-562, myelogenous leukemia) and normal cell line (HEK-293) using MTT assay protocol. The commercially available sorafenib were used in this test as a positive control. The tested cell lines were purchased and dropped on the appropriate growth medium. The growth medium was supplemented with 100 mg/mL of streptomycin, 100 units/mL of penicillin and 10% of heat-inactivated fetal bovine serum in a humidified 5% (v/v) CO2 atmosphere at 37 ◦C. Then the cells from the two cancer cell lines were seeded at the appropriate densities into 96-well microtiter plates. After incubation for 24 h, the growth medium of each cell was treated with graded concentrations (0, 5, 10, 15, 20, 25 and 30 μM) of the test compounds and incubated for three days. The viability of treated cells was determined using 3-[4,5-dimethylthiazole-2-yl]-2,5-diphenyltetrazolium bromide (MTT) technique as cells were stained with 5% MTT solution and allowed to break down the dye into colored-insoluble formazan crystals for 4 h. The formazan crystals were dissolved in acidified isopropanol for 30 min with continuous shaking at room temperature. The colorimetric assay was measured and recorded at absorbance of 570 nm. The cell viability was expressed as percentage of control and the concentration that induces 50% of maximum inhibition of cell proliferation considering control group as 100% viability and group treated with a mixture of toxic compounds as 0% viability. (IC_{50}) were determined for each compound using Graph Pad Prism version 5 software by plotting of log C against % viability.

4.2.2. *In vitro* VEGFR-2 and EGFR kinases assay

The synthesized compounds were estimated for their *in vitro* inhibition on human VEGFR-2 and EGFR in HepG-2 cell line; using ELISA kit. Firstly, a plate was used for the assay had been coated by an antibody specific for human VEGFR-2 and EGFR enzymes, Sorafenib was nominated as a standard VEGFR-2 inhibitor and Erlotnib was used as standard EGFR inhibitor. Both standards and samples were added to the wells and incubated overnight at 4 ◦C, then washed. The biotinylated

antibody was supplemented and further incubated for 1 h at room temperature. The unreacted, liberated antibody was then washed; followed by addition of HRP-conjugated streptavidin and incubated for 45 min at room temperature. Wells were washed and a TMB substrate solution was added and kept at room temperature for 30 min. Finally, the stop solution was added, and the intensity of the color produced was measured at 450 nm. Concentration-inhibition response curve was established by GraphPad Prism 5.0. The IC_{50} value was calculated as the concentration at which 50% of the cells could survive in comparison to sorafenib.

4.2.3. Flow cytometry analysis for cell cycle

Cell cycle analysis for the most potent compounds **8a** and **9a** was carried out through Flow cytometric analysis. In this test HepG-2 cells were supplemented with the test compound at its cytotoxic concentration, seeded and subjected for incubation for 24 h at 37 ◦C and 5% CO2. Cells were washed twice with phosphate buffer saline, then the centrifugation of cell pellets had been completed, followed by preservation with ice-cold 70% ethanol for 15 min. Pellets were collected again and incubated with propidium iodide (PI) staining solution. After incubation for one hour at room temperature, it was analyzed by flowcytometry on an FC500 cytometer (Beckman Coulter) and the cell cycle distributions were calculated.

4.2.4. Quantitative Real Time Reverse-Transcriptase PCR technique

The quantity of Caspase-3, Caspase-9, BAX, Bcl-2, TNF-a, and IL-6R and mRNA in control and the synthesized compounds (at their IC50 concentrations)-treated HepG2 cells was assessed by qRT-PCR. Total RNA from vehicle-treated control (0.01% DMSO) and 10k-treated HepG2 cells were extracted as-per the manufacturer instructions (RNeasy mini kit, Qiagen, Germany). After RNA extraction, cDNA was prepared using the Revert Aid First Strand cDNA Synthesis kit (Thermo Scientific, USA). Amplification of target cDNA for apoptosis markers and GAPDH [as a normalization (housekeeping) gene] was done using one-step RT-PCR SYBR® Green kit Master Mix (Bio-Rad Laboratories, USA) on Rotor-Gene Q real-time PCR thermal cycler instrument. cDNA (2 µl aliquots) was mixed with 1 µl of forward primer, 1 µl reverse primer, 10 µl master mixture, and the reaction volume was completed to 20 µl with nuclease-free water. All experiments were performed in triplicates.

4- Raw data of biological testing; cytotoxicity, VEGFR-2 & EGFR inhibition assay, cell cycle, and expression levels of apoptotic markers

Key codes for the synthesized compounds

Laboratory code	Manuscript code	Structure
**R1**	**8c**	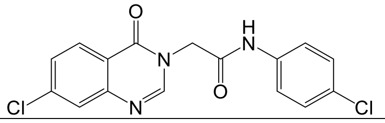
**R2**	**8a**	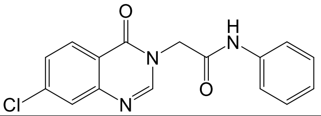
**R3**	**8b**	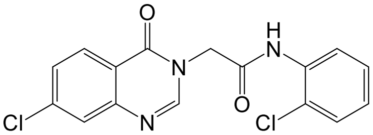
**R4**	**8d**	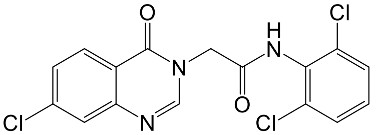
**R5**	**8e**	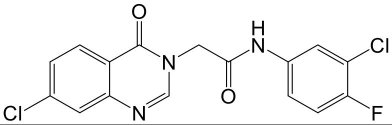
**N1**	**9a**	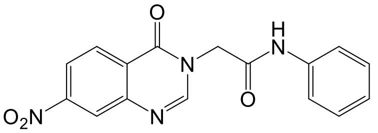
**N2**	**9c**	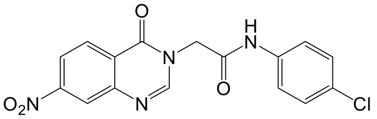
**N3**	**9d**	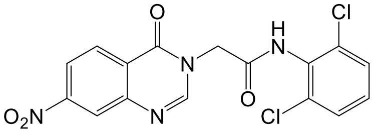
**N4**	**9e**	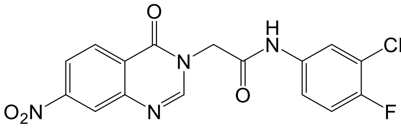
**N5**	**9b**	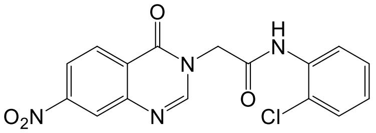

Cytotoxicity against MCF-7 cell line

Viability assay

Institute / Researcher: **Prof. Dr. Alaa Elwan**

Experiment: **functional assay (MTT)**

(Viability/cytotoxicity)

Samples number: **10**

Experiment design: viability against MCF-7 cells

Laboratory comments:

Cytotoxicity against MCF-7 cell line

ID	Conc. umol	O.D			Mean O.D	ST.E	Viability %	Toxicity %	IC50
**MCF7**	dilution	0.767	0.748	0.759	0.758	0.005508	100	0	
N-1	25	0.051	0.049	0.046	0.048667	0.001453	6.420404573	93.579595427	**0.2824**
	12.5	0.053	0.048	0.058	0.053	0.002887	6.992084433	93.007915567	
	6.25	0.063	0.073	0.061	0.065667	0.003712	8.663148637	91.336851363	
	3.125	0.077	0.065	0.084	0.075333	0.005548	9.938434477	90.061565523	
	1.563	0.102	0.107	0.099	0.102667	0.002333	13.544415128	86.455584872	
	0.781	0.21	0.204	0.214	0.209333	0.002906	27.616534741	72.383465259	
	0.391	0.312	0.318	0.321	0.317	0.002646	41.820580475	58.179419525	
	0.195	0.432	0.429	0.427	0.429333	0.001453	56.640281442	43.359718558	
N-2	25	0.042	0.055	0.058	0.051667	0.00491	6.816182938	93.183817062	**0.6724**
	12.5	0.068	0.061	0.063	0.064	0.002082	8.443271768	91.556728232	
	6.25	0.079	0.084	0.092	0.085	0.003786	11.213720317	88.786279683	
	3.125	0.112	0.118	0.115	0.115	0.001732	15.171503958	84.828496042	
	1.563	0.211	0.214	0.205	0.210	0.002646	27.704485488	72.295514512	
	0.781	0.327	0.329	0.321	0.325667	0.002404	42.963940193	57.036059807	
	0.391	0.515	0.511	0.522	0.516	0.003215	68.073878628	31.926121372	
	0.195	0.687	0.698	0.692	0.692333	0.00318	91.336851363	8.663148637	
N-3	25	0.049	0.058	0.055	0.054	0.002646	7.124010554	92.875989446	**0.2042**
	12.5	0.059	0.045	0.042	0.048667	0.005239	6.420404573	93.579595427	
	6.25	0.068	0.051	0.062	0.060333	0.004978	7.959542656	92.040457344	
	3.125	0.068	0.065	0.071	0.068	0.001732	8.970976253	91.029023747	
	1.563	0.086	0.61	0.083	0.259667	0.175169	34.256816183	65.743183817	
	0.781	0.196	0.202	0.192	0.196667	0.002906	25.945470536	74.054529464	
	0.391	0.304	0.283	0.288	0.291667	0.006333	38.478452067	61.521547933	
	0.195	0.385	0.387	0.378	0.383333	0.002728	50.571679859	49.428320141	
N-4	25	0.053	0.045	0.050	0.049333	0.002333	6.508355321	93.491644679	**0.2314**
	12.5	0.062	0.058	0.067	0.062333	0.002603	8.223394899	91.776605101	
	6.25	0.071	0.075	0.064	0.070	0.003215	9.234828496	90.765171504	
	3.125	0.078	0.064	0.083	0.075	0.005686	9.894459103	90.105540897	
	1.563	0.099	0.104	0.108	0.103667	0.002603	13.676341249	86.323658751	
	0.781	0.158	0.163	0.154	0.158333	0.002603	20.888302551	79.111697449	
	0.391	0.224	0.218	0.22	0.220667	0.001764	29.111697449	70.888302551	
	0.195	0.421	0.414	0.411	0.415333	0.002963	54.793315743	45.206684257	
N-5	25	0.058	0.051	0.045	0.051333	0.003756	6.772207564	93.227792436	**0.2090**
	12.5	0.055	0.053	0.057	0.055	0.001155	7.255936675	92.744063325	
	6.25	0.061	0.067	0.054	0.060667	0.003756	8.003518030	91.996481970	
	3.125	0.066	0.072	0.077	0.071667	0.00318	9.454705365	90.545294635	
	1.563	0.095	0.084	0.091	0.090	0.003215	11.873350923	88.126649077	
	0.781	0.119	0.112	0.123	0.118	0.003215	15.567282322	84.432717678	
	0.391	0.174	0.181	0.162	0.172333	0.005548	22.735268250	77.264731750	
	0.195	0.401	0.387	0.397	0.395	0.004163	52.110817942	47.889182058	

Cytotoxicity against MCF-7 cell line

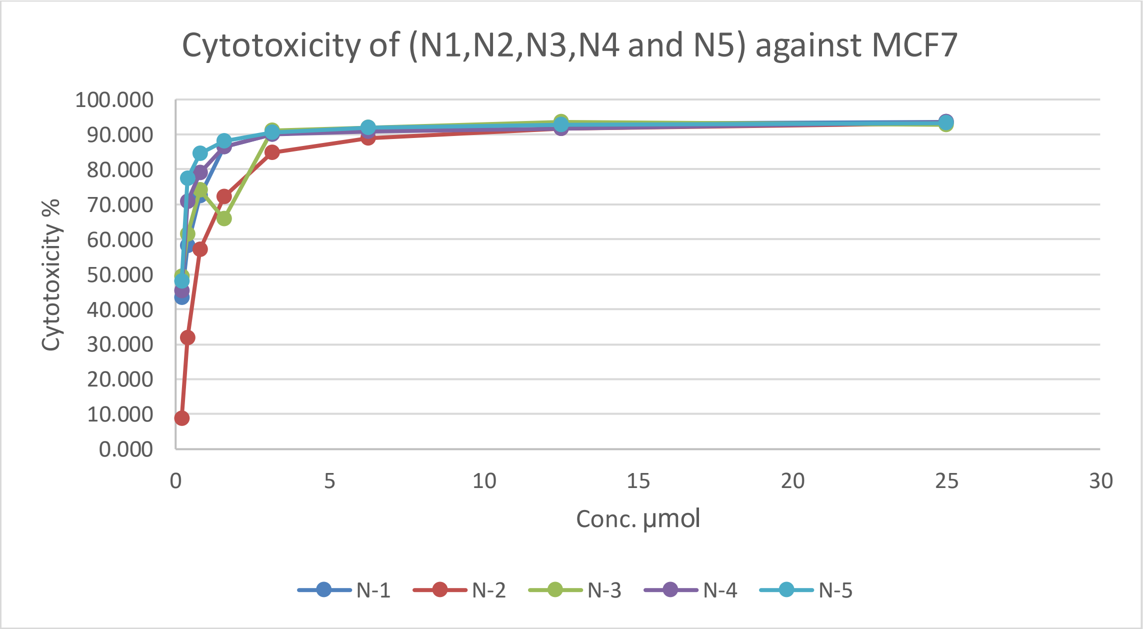

Cytotoxicity against MCF-7 cell line

ID	Conc. umol	O.D			Mean O.D	ST.E	Viability %	Toxicity %	IC50
**MCF7**	dilution	0.767	0.748	0.759	0.758	0.005508	100	0	
R-1	25	0.045	0.053	0.053	0.050333	0.002667	6.640281442	93.359718558	**0.1875**
	12.5	0.055	0.066	0.062	0.061	0.003215	8.047493404	91.952506596	
	6.25	0.067	0.056	0.069	0.064	0.004041	8.443271768	91.556728232	
	3.125	0.072	0.076	0.084	0.077333	0.003528	10.202286719	89.797713281	
	1.563	0.098	0.092	0.107	0.099	0.004359	13.060686016	86.939313984	
	0.781	0.161	0.154	0.146	0.153667	0.004333	20.272647318	79.727352682	
	0.391	0.248	0.246	0.255	0.249667	0.002728	32.937554969	67.062445031	
	0.195	0.358	0.342	0.349	0.349667	0.004631	46.130167106	53.869832894	
R-2	25	0.052	0.065	0.048	0.055	0.005132	7.255936675	92.744063325	**0.6955**
	12.5	0.066	0.062	0.061	0.063	0.001528	8.311345646	91.688654354	
	6.25	0.078	0.094	0.087	0.086333	0.004631	11.389621812	88.610378188	
	3.125	0.143	0.137	0.125	0.135	0.005292	17.810026385	82.189973615	
	1.563	0.220	0.215	0.213	0.216	0.002082	28.496042216	71.503957784	
	0.781	0.339	0.346	0.342	0.342333	0.002028	45.162708883	54.837291117	
	0.391	0.508	0.514	0.502	0.508	0.003464	67.018469657	32.981530343	
	0.195	0.711	0.709	0.706	0.708667	0.001453	93.491644679	6.508355321	
R-3	25	0.044	0.050	0.058	0.050667	0.004055	6.684256816	93.315743184	**0.1908**
	12.5	0.059	0.042	0.055	0.052	0.005132	6.860158311	93.139841689	
	6.25	0.057	0.064	0.067	0.062667	0.002963	8.267370273	91.732629727	
	3.125	0.061	0.084	0.078	0.074333	0.006888	9.806508355	90.193491645	
	1.563	0.086	0.092	0.094	0.090667	0.002404	11.961301671	88.038698329	
	0.781	0.108	0.102	0.111	0.107	0.002646	14.116094987	85.883905013	
	0.391	0.211	0.222	0.218	0.217	0.003215	28.627968338	71.372031662	
	0.195	0.366	0.358	0.364	0.362667	0.002404	47.845206684	52.154793316	
R-4	25	0.055	0.064	0.067	0.062	0.003606	8.179419525	91.820580475	**0.7462**
	12.5	0.077	0.072	0.062	0.070333	0.00441	9.278803870	90.721196130	
	6.25	0.084	0.092	0.081	0.085667	0.003283	11.301671064	88.698328936	
	3.125	0.141	0.135	0.124	0.133333	0.004978	17.590149516	82.409850484	
	1.563	0.219	0.224	0.215	0.219333	0.002603	28.935795954	71.064204046	
	0.781	0.364	0.371	0.368	0.367667	0.002028	48.504837291	51.495162709	
	0.391	0.49	0.493	0.491	0.491333	0.000882	64.819700967	35.180299033	
	0.195	0.667	0.678	0.674	0.673	0.003215	88.786279683	11.213720317	
R-5	25	0.049	0.052	0.046	0.049	0.001732	6.464379947	93.535620053	**0.5523**
	12.5	0.055	0.057	0.062	0.058	0.002082	7.651715040	92.348284960	
	6.25	0.068	0.064	0.073	0.068333	0.002603	9.014951627	90.985048373	
	3.125	0.096	0.105	0.089	0.096667	0.004631	12.752858399	87.247141601	
	1.563	0.148	0.141	0.144	0.144333	0.002028	19.041336851	80.958663149	
	0.781	0.254	0.248	0.242	0.248	0.003464	32.717678100	67.282321900	
	0.391	0.472	0.478	0.463	0.471	0.004359	62.137203166	37.862796834	
	0.195	0.612	0.627	0.615	0.618	0.004583	81.530343008	18.469656992	

Cytotoxicity against MCF-7 cell line

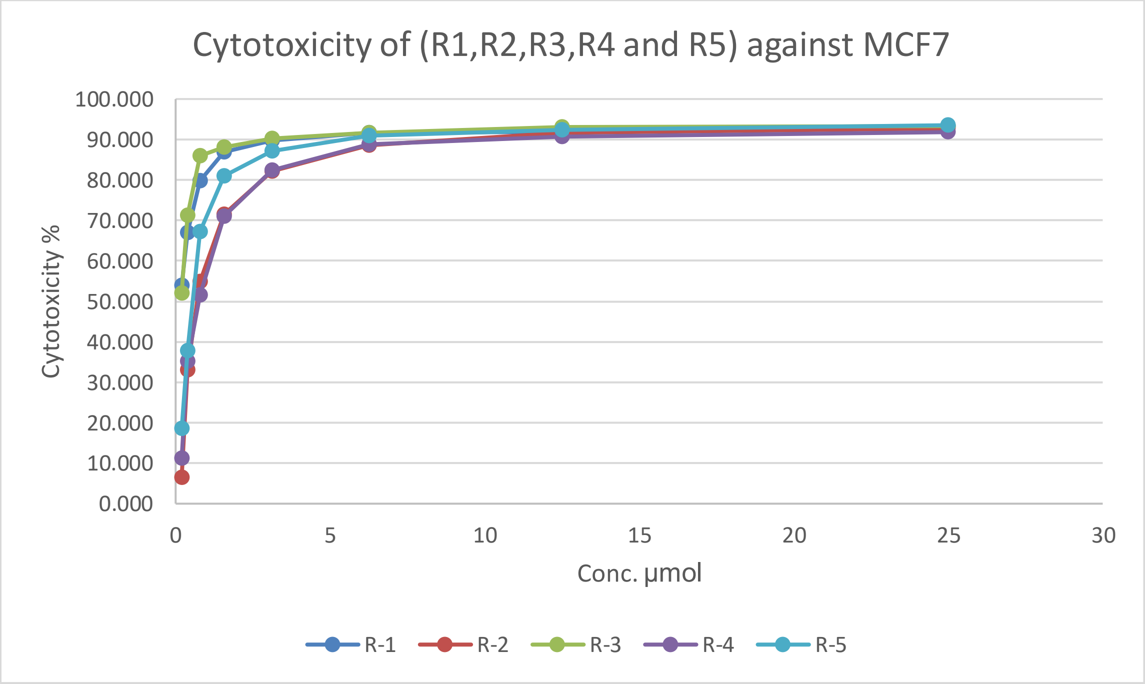

Cytotoxicity against HepG-2 cell line

Viability assay

**Institute / Researcher:**	**Dr. Alaa Elwan**
**Experiment:**	**functional assay (MTT)**<br>**(Viability / Cytotoxicity)**
**Samples number:**	**10**

Experiment design: viability against HepG2 cells

Laboratory comments:

Cytotoxicity against HepG-2 cell line

ID	Conc. ug/ml	O.D			Mean O.D	ST.E	Viability %	Toxicity %	IC50
HepG2	dilution	0.902	0.916	0.912	0.910	0.004163	100	0	
N1	25	0.055	0.052	0.049	0.052	0.001732	5.714285714	94.285714286	**0.1871**
	12.5	0.048	0.061	0.056	0.055	0.003786	6.043956044	93.956043956	
	6.25	0.064	0.074	0.052	0.063333	0.00636	6.959706960	93.040293040	
	3.125	0.091	0.082	0.088	0.087	0.002646	9.560439560	90.439560440	
	1.563	0.12	0.116	0.112	0.116	0.002309	12.747252747	87.252747253	
	0.781	0.19	0.195	0.189	0.191333	0.001856	21.025641026	78.974358974	
	0.391	0.274	0.27	0.267	0.270333	0.002028	29.706959707	70.293040293	
	0.195	0.424	0.417	0.413	0.418	0.003215	45.934065934	54.065934066	
N2	25	0.055	0.049	0.066	0.056667	0.004978	6.227106227	93.772893773	**1.0001**
	12.5	0.071	0.066	0.064	0.067	0.002082	7.362637363	92.637362637	
	6.25	0.106	0.104	0.110	0.106667	0.001764	11.721611722	88.278388278	
	3.125	0.161	0.155	0.143	0.153	0.005292	16.813186813	83.186813187	
	1.563	0.304	0.321	0.314	0.313	0.004933	34.395604396	65.604395604	
	0.781	0.518	0.509	0.504	0.510333	0.004096	56.080586081	43.919413919	
	0.391	0.637	0.64	0.641	0.639333	0.001202	70.256410256	29.743589744	
	0.195	0.755	0.759	0.763	0.759	0.002309	83.406593407	16.593406593	
N3	25	0.062	0.058	0.052	0.057333	0.002906	6.300366300	93.699633700	**0.3227**
	12.5	0.063	0.055	0.068	0.062	0.003786	6.813186813	93.186813187	
	6.25	0.081	0.069	0.072	0.074	0.003606	8.131868132	91.868131868	
	3.125	0.092	0.098	0.103	0.097667	0.00318	10.732600733	89.267399267	
	1.563	0.135	0.141	0.133	0.136333	0.002404	14.981684982	85.018315018	
	0.781	0.272	0.267	0.263	0.267333	0.002603	29.377289377	70.622710623	
	0.391	0.407	0.405	0.398	0.403333	0.002728	44.322344322	55.677655678	
	0.195	0.548	0.554	0.557	0.553	0.002646	60.769230769	39.230769231	
N4	25	0.059	0.062	0.066	0.062333	0.002028	6.849816850	93.150183150	**1.5103**
	12.5	0.075	0.083	0.071	0.076333	0.003528	8.388278388	91.611721612	
	6.25	0.148	0.142	0.154	0.148	0.003464	16.263736264	83.736263736	
	3.125	0.236	0.24	0.231	0.235667	0.002603	25.897435897	74.102564103	
	1.563	0.456	0.448	0.451	0.451667	0.002333	49.633699634	50.366300366	
	0.781	0.502	0.500	0.504	0.502	0.001155	55.164835165	44.835164835	
	0.391	0.572	0.584	0.589	0.581667	0.005044	63.919413919	36.080586081	
	0.195	0.797	0.802	0.791	0.796667	0.00318	87.545787546	12.454212454	
N5	25	0.065	0.055	0.042	0.054	0.006658	5.934065934	94.065934066	**0.1944**
	12.5	0.054	0.062	0.067	0.061	0.003786	6.703296703	93.296703297	
	6.25	0.071	0.069	0.073	0.071	0.001155	7.802197802	92.197802198	
	3.125	0.091	0.094	0.086	0.090333	0.002333	9.926739927	90.073260073	
	1.563	0.122	0.113	0.119	0.118	0.002646	12.967032967	87.032967033	
	0.781	0.246	0.259	0.251	0.252	0.003786	27.692307692	72.307692308	
	0.391	0.344	0.335	0.339	0.339333	0.002603	37.289377289	62.710622711	
	0.195	0.449	0.455	0.452	0.452	0.001732	49.670329670	50.329670330	

Cytotoxicity against HepG-2 cell line

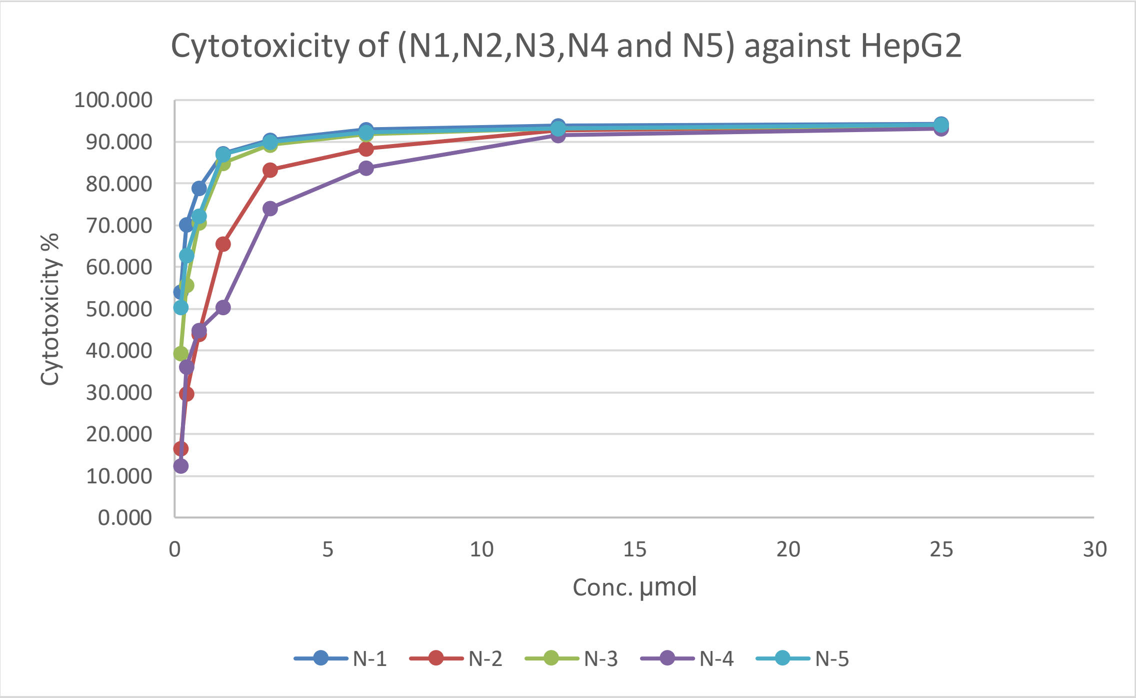

Cytotoxicity against HepG-2 cell line

ID	Conc. ug/ml	O.D			Mean O.D	ST.E	Viability %	Toxicity %	IC50
HepG2	dilution	0.902	0.916	0.912	0.910	0.004163	100	0	
R1	25	0.057	0.061	0.065	0.061	0.002309	6.703296703	93.296703297	**0.7344**
	12.5	0.065	0.072	0.072	0.069667	0.002333	7.655677656	92.344322344	
	6.25	0.108	0.102	0.091	0.100333	0.004978	11.025641026	88.974358974	
	3.125	0.144	0.142	0.149	0.145	0.002082	15.934065934	84.065934066	
	1.563	0.275	0.274	0.266	0.271667	0.002848	29.853479853	70.146520147	
	0.781	0.434	0.436	0.435	0.435	0.000577	47.802197802	52.197802198	
	0.391	0.594	0.600	0.604	0.599333	0.002906	65.860805861	34.139194139	
	0.195	0.811	0.808	0.815	0.811333	0.002028	89.157509158	10.842490842	
R2	25	0.050	0.056	0.055	0.053667	0.001856	5.897435897	94.102564103	**0.1871**
	12.5	0.064	0.058	0.062	0.061333	0.001764	6.739926740	93.260073260	
	6.25	0.077	0.072	0.066	0.071667	0.00318	7.875457875	92.124542125	
	3.125	0.088	0.064	0.085	0.079	0.00755	8.681318681	91.318681319	
	1.563	0.102	0.108	0.111	0.107	0.002646	11.758241758	88.241758242	
	0.781	0.157	0.148	0.152	0.152333	0.002603	16.739926740	83.260073260	
	0.391	0.232	0.247	0.243	0.240667	0.004485	26.446886447	73.553113553	
	0.195	0.417	0.425	0.412	0.418	0.003786	45.934065934	54.065934066	
R3	25	0.055	0.051	0.049	0.051667	0.001764	5.677655678	94.322344322	**0.2242**
	12.5	0.054	0.075	0.063	0.064	0.006083	7.032967033	92.967032967	
	6.25	0.069	0.084	0.076	0.076333	0.004333	8.388278388	91.611721612	
	3.125	0.089	0.094	0.081	0.088	0.003786	9.670329670	90.329670330	
	1.563	0.121	0.129	0.124	0.124667	0.002333	13.699633700	86.300366300	
	0.781	0.256	0.254	0.242	0.250667	0.004372	27.545787546	72.454212454	
	0.391	0.294	0.315	0.301	0.303333	0.006173	33.333333333	66.666666667	
	0.195	0.484	0.483	0.478	0.481667	0.001856	52.930402930	47.069597070	
R4	25	0.052	0.073	0.062	0.062333	0.006064	6.849816850	93.150183150	**0.6358**
	12.5	0.063	0.082	0.075	0.073333	0.005548	8.058608059	91.941391941	
	6.25	0.074	0.092	0.083	0.083	0.005196	9.120879121	90.879120879	
	3.125	0.107	0.092	0.101	0.100	0.004359	10.989010989	89.010989011	
	1.563	0.129	0.125	0.122	0.125333	0.002028	13.772893773	86.227106227	
	0.781	0.382	0.387	0.396	0.388333	0.004096	42.673992674	57.326007326	
	0.391	0.568	0.562	0.57	0.566667	0.002404	62.271062271	37.728937729	
	0.195	0.794	0.792	0.798	0.794667	0.001764	87.326007326	12.673992674	
R5	25	0.068	0.052	0.077	0.065667	0.007311	7.216117216	92.783882784	**1.4357**
	12.5	0.087	0.079	0.092	0.086	0.003786	9.450549451	90.549450549	
	6.25	0.097	0.119	0.108	0.108	0.006351	11.868131868	88.131868132	
	3.125	0.209	0.215	0.207	0.210333	0.002404	23.113553114	76.886446886	
	1.563	0.436	0.426	0.438	0.433333	0.003712	47.619047619	52.380952381	
	0.781	0.562	0.569	0.571	0.567333	0.002728	62.344322344	37.655677656	
	0.391	0.682	0.694	0.687	0.687667	0.00348	75.567765568	24.432234432	
	0.195	0.866	0.842	0.854	0.854	0.006928	93.846153846	6.153846154	

Cytotoxicity against HepG-2 cell line

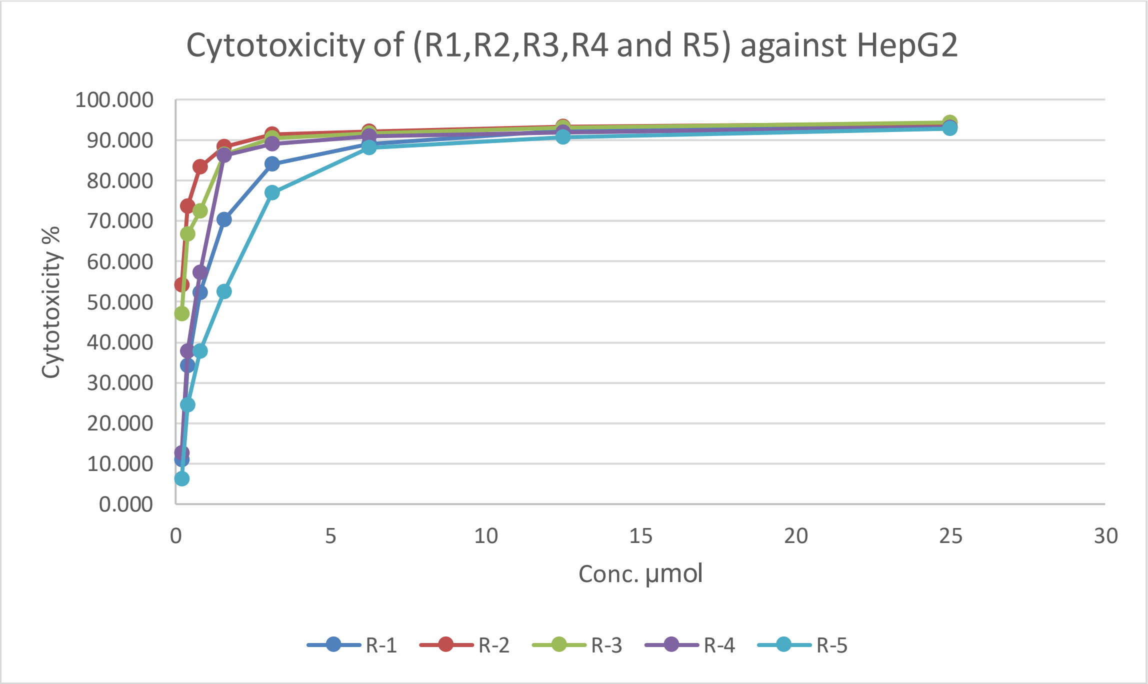

Cytotoxicity against K-562 cell line

Viability assay

Institute / Researcher: **Prof. Dr. Alaa Elwan**

Experiment: **functional assay (MTT)**
(Viability/cytotoxicity)

Samples number: **10**

Experiment design: viability against K-562 cells

Laboratory comments:

Cytotoxicity against K-562 cell line

ID	Conc. umol	O.D			Mean O.D	ST.E	Viability %	Toxicity %	IC50
**K-562**	dilution	0.801	0.816	0.819	0.812	0.005568	100	0	
N-1	25	0.063	0.052	0.056	0.057	0.003215	7.019704433	92.980295567	**0.3858**
	12.5	0.065	0.055	0.069	0.063	0.004163	7.758620690	92.241379310	
	6.25	0.072	0.068	0.074	0.071333	0.001764	8.784893268	91.215106732	
	3.125	0.067	0.072	0.078	0.072333	0.00318	8.908045977	91.091954023	
	1.563	0.132	0.136	0.134	0.134	0.001155	16.502463054	83.497536946	
	0.781	0.249	0.247	0.254	0.250	0.002082	30.788177340	69.211822660	
	0.391	0.394	0.412	0.403	0.403	0.005196	49.630541872	50.369458128	
	0.195	0.548	0.534	0.541	0.541	0.004041	66.625615764	33.374384236	
N-2	25	0.054	0.06	0.057	0.057	0.001732	7.019704433	92.980295567	**0.5388**
	12.5	0.061	0.058	0.062	0.060333	0.001202	7.430213465	92.569786535	
	6.25	0.062	0.067	0.073	0.067333	0.00318	8.292282430	91.707717570	
	3.125	0.089	0.097	0.082	0.089333	0.004333	11.001642036	88.998357964	
	1.563	0.187	0.172	0.184	0.181	0.004583	22.290640394	77.709359606	
	0.781	0.277	0.274	0.267	0.272667	0.002963	33.579638752	66.420361248	
	0.391	0.482	0.492	0.487	0.487	0.002887	59.975369458	40.024630542	
	0.195	0.659	0.648	0.657	0.654667	0.003383	80.623973727	19.376026273	
N-3	25	0.059	0.067	0.055	0.060333	0.003528	7.430213465	92.569786535	**2.2742**
	12.5	0.085	0.072	0.074	0.077	0.004041	9.482758621	90.517241379	
	6.25	0.105	0.094	0.098	0.099	0.003215	12.192118227	87.807881773	
	3.125	0.302	0.312	0.307	0.307	0.002887	37.807881773	62.192118227	
	1.563	0.485	0.492	0.489	0.488667	0.002028	60.180623974	39.819376026	
	0.781	0.581	0.586	0.577	0.581333	0.002603	71.592775041	28.407224959	
	0.391	0.788	0.771	0.776	0.778333	0.005044	95.853858785	4.146141215	
	0.195	0.804	0.814	0.807	0.808333	0.002963	99.548440066	0.451559934	
N-4	25	0.057	0.053	0.049	0.053	0.002309	6.527093596	93.472906404	**0.3469**
	12.5	0.065	0.071	0.062	0.066	0.002646	8.128078818	91.871921182	
	6.25	0.084	0.086	0.092	0.087333	0.002404	10.755336617	89.244663383	
	3.125	0.118	0.104	0.112	0.111333	0.004055	13.711001642	86.288998358	
	1.563	0.161	0.145	0.157	0.154333	0.004807	19.006568144	80.993431856	
	0.781	0.222	0.211	0.215	0.216	0.003215	26.600985222	73.399014778	
	0.391	0.389	0.384	0.372	0.381667	0.005044	47.003284072	52.996715928	
	0.195	0.491	0.495	0.489	0.491667	0.001764	60.550082102	39.449917898	
N-5	25	0.057	0.05	0.044	0.050333	0.003756	6.198686371	93.801313629	**0.1902**
	12.5	0.06	0.055	0.052	0.055667	0.002333	6.855500821	93.144499179	
	6.25	0.065	0.069	0.073	0.069	0.002309	8.497536946	91.502463054	
	3.125	0.076	0.062	0.058	0.065333	0.005457	8.045977011	91.954022989	
	1.563	0.106	0.112	0.114	0.110667	0.002404	13.628899836	86.371100164	
	0.781	0.212	0.207	0.202	0.207	0.002887	25.492610837	74.507389163	
	0.391	0.302	0.312	0.307	0.307	0.002887	37.807881773	62.192118227	
	0.195	0.384	0.385	0.389	0.386	0.001528	47.536945813	52.463054187	

Cytotoxicity against K-562 cell line

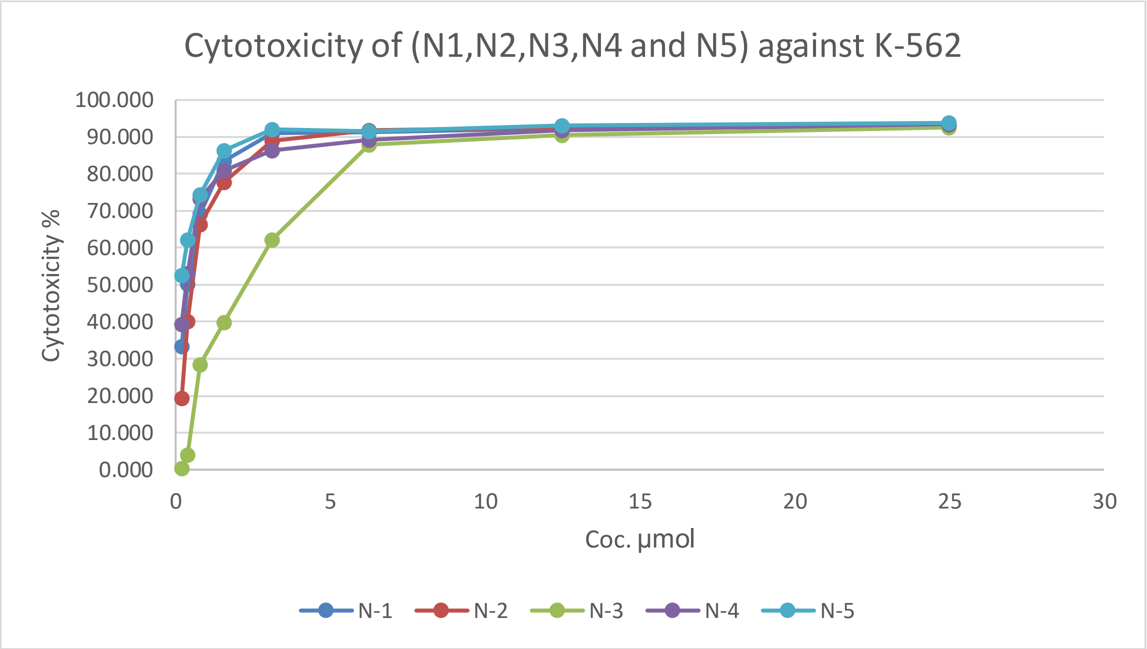

Cytotoxicity against K-562 cell line

ID	Conc. umol	O.D			Mean O.D	ST.E	Viability %	Toxicity %	IC50
**K562**	dilution	0.801	0.816	0.819	0.812	0.005568	100	0	
R-1	25	0.054	0.047	0.058	0.053	0.003215	6.527093596	93.472906404	**0.5444**
	12.5	0.071	0.063	0.067	0.067	0.002309	8.251231527	91.748768473	
	6.25	0.084	0.082	0.075	0.080333	0.002728	9.893267652	90.106732348	
	3.125	0.107	0.112	0.098	0.105667	0.004096	13.013136289	86.986863711	
	1.563	0.159	0.164	0.152	0.158333	0.00348	19.499178982	80.500821018	
	0.781	0.283	0.279	0.275	0.279	0.002309	34.359605911	65.640394089	
	0.391	0.487	0.493	0.484	0.488	0.002646	60.098522167	39.901477833	
	0.195	0.650	0.654	0.665	0.656333	0.004485	80.829228243	19.170771757	
R-2	25	0.045	0.048	0.042	0.045	0.001732	5.541871921	94.458128079	**0.1884**
	12.5	0.049	0.045	0.053	0.049	0.002309	6.034482759	93.965517241	
	6.25	0.058	0.052	0.065	0.058333	0.003756	7.183908046	92.816091954	
	3.125	0.071	0.064	0.067	0.067333	0.002028	8.292282430	91.707717570	
	1.563	0.102	0.107	0.101	0.103333	0.001856	12.725779967	87.274220033	
	0.781	0.172	0.174	0.189	0.178333	0.005364	21.962233169	78.037766831	
	0.391	0.244	0.237	0.232	0.237667	0.00348	29.269293924	70.730706076	
	0.195	0.375	0.374	0.387	0.378667	0.004177	46.633825944	53.366174056	
R-3	25	0.048	0.055	0.06	0.054333	0.00348	6.691297209	93.308702791	**0.4642**
	12.5	0.065	0.066	0.059	0.063333	0.002186	7.799671593	92.200328407	
	6.25	0.063	0.074	0.071	0.069333	0.003283	8.538587849	91.461412151	
	3.125	0.097	0.092	0.081	0.090	0.004726	11.083743842	88.916256158	
	1.563	0.131	0.137	0.132	0.133333	0.001856	16.420361248	83.579638752	
	0.781	0.219	0.221	0.227	0.222333	0.002404	27.380952381	72.619047619	
	0.391	0.453	0.444	0.448	0.448333	0.002603	55.213464696	44.786535304	
	0.195	0.674	0.677	0.663	0.671333	0.004256	82.676518883	17.323481117	
R-4	25	0.62	0.065	0.052	0.245667	0.187204	30.254515599	69.745484401	**1.6713**
	12.5	0.061	0.078	0.070	0.069667	0.00491	8.579638752	91.420361248	
	6.25	0.113	0.108	0.115	0.112	0.002082	13.793103448	86.206896552	
	3.125	0.284	0.287	0.271	0.280667	0.00491	34.564860427	65.435139573	
	1.563	0.419	0.415	0.412	0.415333	0.002028	51.149425287	48.850574713	
	0.781	0.542	0.547	0.546	0.545	0.001528	67.118226601	32.881773399	
	0.391	0.713	0.71	0.718	0.713667	0.002333	87.889983580	12.110016420	
	0.195	0.807	0.811	0.818	0.812	0.003215	100.000	0.000000000	
R-5	25	0.051	0.048	0.045	0.048	0.001732	5.911330049	94.088669951	**0.2664**
	12.5	0.071	0.058	0.052	0.060333	0.005608	7.430213465	92.569786535	
	6.25	0.078	0.062	0.074	0.071333	0.004807	8.784893268	91.215106732	
	3.125	0.091	0.085	0.076	0.084	0.004359	10.344827586	89.655172414	
	1.563	0.172	0.164	0.177	0.171	0.003786	21.059113300	78.940886700	
	0.781	0.26	0.254	0.255	0.256333	0.001856	31.568144499	68.431855501	
	0.391	0.362	0.371	0.369	0.367333	0.002728	45.238095238	54.761904762	
	0.195	0.427	0.435	0.423	0.428333	0.003528	52.750410509	47.249589491	

Cytotoxicity against K-562 cell line

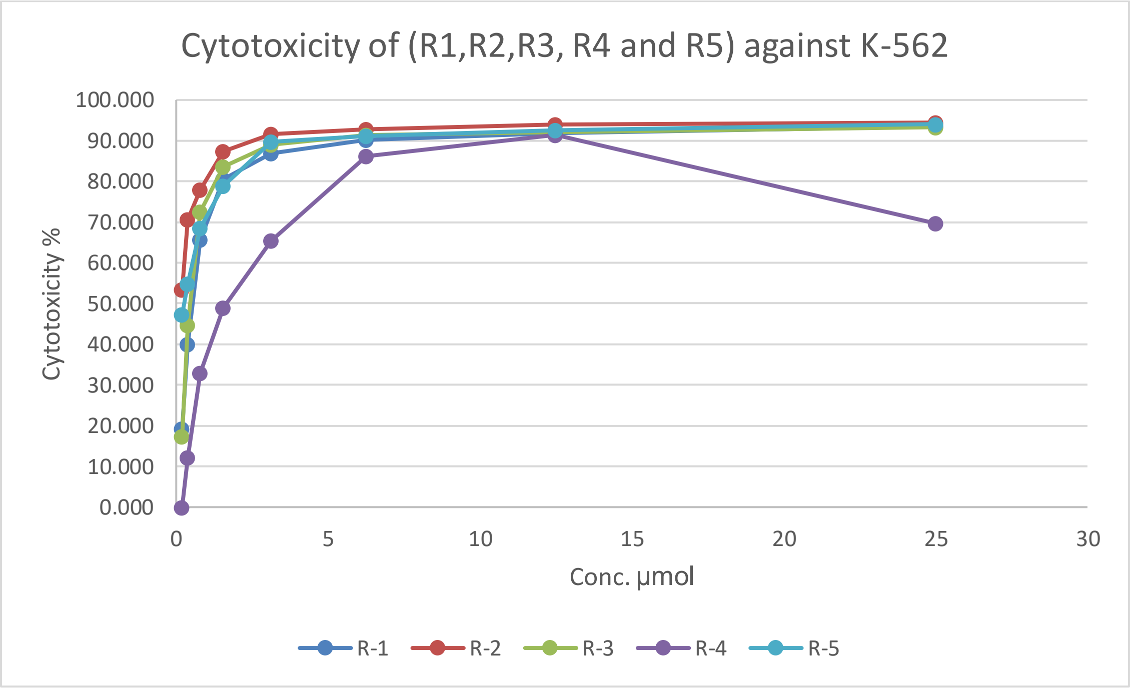

Sorafenib (Nexavar) cytotoxicity against (MCF7, HepG2 and K-562) cells

ID	Conc. umol/ml	O.D			Mean O.D	ST.E	Viability %	Toxicity %	IC50
**MCF7**	dilution	0.645	0.661	0.647	0.651	0.005033	100	0	
Nexavar	10	0.059	0.053	0.048	0.053333	0.00318	8.192524322	91.807475678	**0.1283**
	50	0.063	0.065	0.064	0.064	0.000577	9.831029186	90.168970814	
	2.5	0.078	0.063	0.072	0.071	0.004359	10.906298003	89.093701997	
	1.25	0.073	0.068	0.088	0.076333	0.006009	11.725550435	88.274449565	
	0.625	0.093	0.114	0.108	0.105	0.006245	16.129032258	83.870967742	
	0.313	0.163	0.167	0.172	0.167333	0.002603	25.704045059	74.295954941	
	0.156	0.247	0.244	0.254	0.248333	0.002963	38.146441372	61.853558628	
	0.078	0.473	0.465	0.459	0.465667	0.004055	71.530977983	28.469022017	

ID	Conc. umol/ml	O.D			Mean O.D	ST.E	Viability %	Toxicity %	IC50
**HepG2**	dilution	0.808	0.812	0.828	0.816	0.00611	100	0	
Nexavar	10	0.06	0.053	0.055	0.056	0.002082	6.862745098	93.137254902	**0.0844**
	50	0.058	0.065	0.066	0.063	0.002517	7.720588235	92.279411765	
	2.5	0.065	0.059	0.069	0.064333	0.002906	7.883986928	92.116013072	
	1.25	0.074	0.062	0.077	0.071	0.004583	8.700980392	91.299019608	
	0.625	0.083	0.091	0.098	0.090667	0.004333	11.111111111	88.888888889	
	0.313	0.131	0.132	0.142	0.135	0.003512	16.544117647	83.455882353	
	0.156	0.257	0.254	0.243	0.251333	0.004256	30.800653595	69.199346405	
	0.078	0.427	0.415	0.424	0.422	0.003606	51.715686275	48.284313725	

ID	Conc. umol/ml	O.D			Mean O.D	ST.E	Viability %	Toxicity %	IC50
**K-562**	dilution	0.754	0.739	0.751	0.748	0.004583	100	0	
Nexavar	10	0.058	0.049	0.052	0.053	0.002646	7.085561497	92.914438503	**0.0606**
	50	0.055	0.068	0.066	0.063	0.004041	8.422459893	91.577540107	
	2.5	0.059	0.064	0.055	0.059333	0.002603	7.932263815	92.067736185	
	1.25	0.076	0.076	0.064	0.072	0.00400	9.625668449	90.374331551	
	0.625	0.073	0.061	0.072	0.068667	0.003844	9.180035651	90.819964349	
	0.313	0.098	0.087	0.092	0.092333	0.00318	12.344028520	87.655971480	
	0.156	0.127	0.124	0.134	0.128333	0.002963	17.156862745	82.843137255	
	0.078	0.293	0.295	0.284	0.290667	0.003383	38.859180036	61.140819964	

Cytotoxicity against normal cell (HEK-293)

Viability assay

Institute / Researcher: **Prof. Dr. Alaa Elwan**

Experiment: **functional assay (MTT)**

(Viability/cytotoxicity)

Samples number: **1**

Experiment design: viability against HEK-293 cells

Laboratory comments:

Cytotoxicity against normal cell (HEK-293)

ID	Conc. umol/ml	O.D			Mean O.D	ST.E	Viability %	Toxicity %	IC50
**HEK**	dilution	0.687	0.673	0.689	0.683	0.005033	100	0	
N5	25	0.048	0.042	0.039	0.043	0.002646	6.295754026	93.704245974	**1.7468**
	12.5	0.067	0.059	0.054	0.060	0.003786	8.784773060	91.215226940	
	6.25	0.113	0.118	0.121	0.117333	0.002333	17.179111762	82.820888238	
	3.125	0.207	0.204	0.197	0.202667	0.002963	29.673011225	70.326988775	
	1.563	0.364	0.359	0.357	0.360	0.002082	52.708638360	47.291361640	
	0.781	0.433	0.421	0.419	0.424333	0.004372	62.127867252	37.872132748	
	0.391	0.525	0.522	0.528	0.525	0.001732	76.866764275	23.133235725	
	0.195	0.673	0.666	0.671	0.670	0.002082	98.096632504	1.903367496	

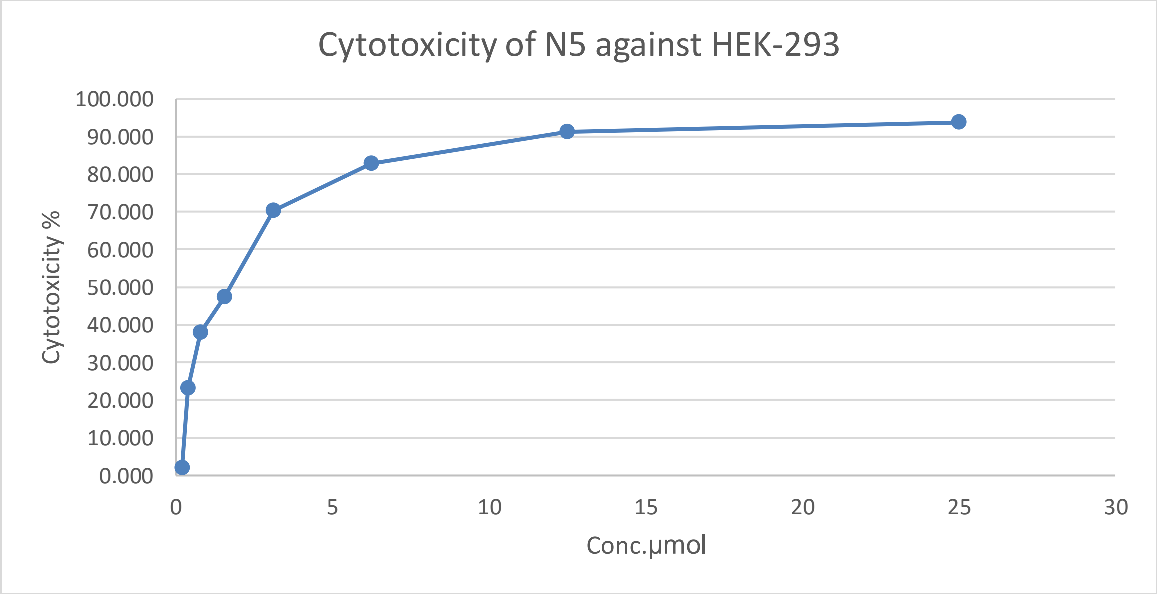

Cytotoxicity against normal cell (HEK-293)

ID	Conc. umol/ml	O.D			Mean O.D	ST.E	Viability %	Toxicity %	IC50
HEK	dilution	0.687	0.673	0.689	0.683	0.005033	100	0	
Nexavar.	10	0.044	0.048	0.037	0.043	0.003215	6.295754026	93.704245974	0.1310
	50	0.062	0.051	0.064	0.059	0.004041	8.638360176	91.361639824	
	2.5	0.073	0.068	0.071	0.070667	0.001453	10.346510493	89.653489507	
	1.25	0.087	0.084	0.097	0.089333	0.00393	13.079551000	86.920449000	
	0.625	0.124	0.119	0.117	0.120	0.002082	17.569546120	82.430453880	
	0.313	0.213	0.211	0.209	0.211	0.001155	30.893118594	69.106881406	
	0.156	0.285	0.272	0.280	0.279	0.003786	40.849194729	59.150805271	
	0.078	0.474	0.476	0.471	0.473667	0.001453	69.350902879	30.649097121	

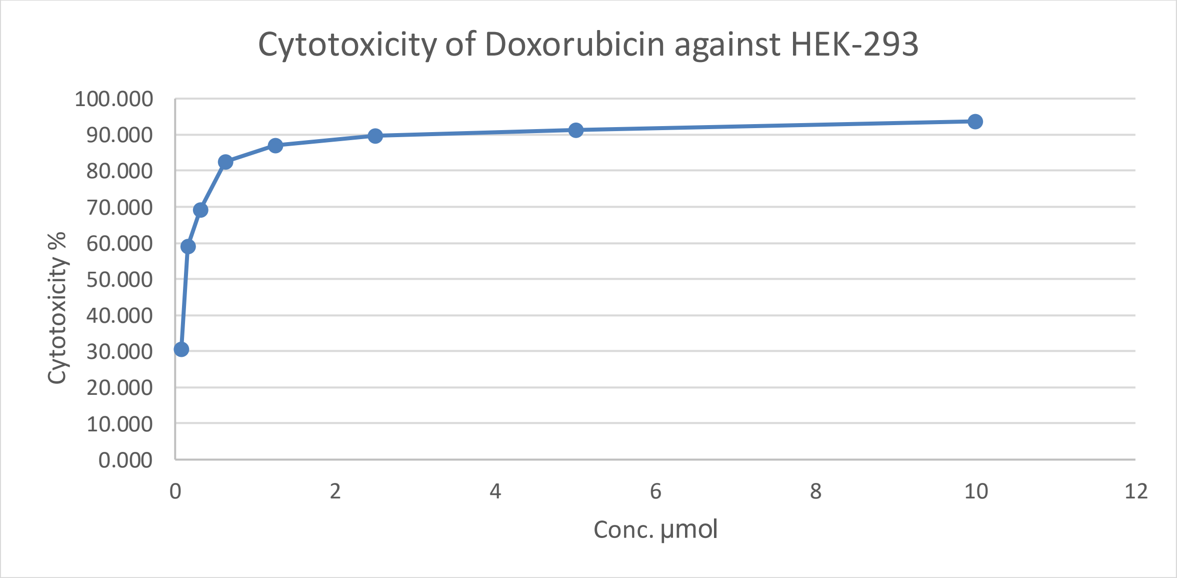

Enzyme assay (VEGFR-2 kinase):

A- MCF7

code	RLU			Mean	Activity %	Inhibition %
**MCF7**	**130.2**	**127.7**	**125.8**	**127.9000**	**100.000**	**0.000**
**Nexavar**	**14.6**	**10.27**	**21.2**	**15.3567**	**12.007**	**87.993**
**N1**	**28.9**	**23.3**	**24.4**	**25.5333**	**19.964**	**80.036**
**N3**	**66.4**	**75.6**	**61.2**	**67.7333**	**52.958**	**47.042**
**N5**	**77.27**	**119.1**	**113.2**	**103.1900**	**80.680**	**19.320**
**R2**	**32.18**	**50.8**	**41.25**	**41.4100**	**32.377**	**67.623**
**R3**	**25.5**	**26.6**	**21.8**	**24.6333**	**19.260**	**80.740**

B- HepG2

code	RLU			Mean	Activity %	Inhibition %
**HepG2**	**473.31**	**452.47**	**452.39**	**459.3900**	**100.000**	**0.000**
**Nexavar**	**32.06**	**35.22**	**32.29**	**33.1900**	**7.225**	**92.775**
**N1**	**89.28**	**47.46**	**66.68**	**67.8067**	**14.760**	**85.240**
**N3**	**141.1**	**81.87**	**50.15**	**91.0400**	**19.818**	**80.182**
**N5**	**113.1**	**174.3**	**175.17**	**154.1900**	**33.564**	**66.436**
**R2**	**201.2**	**57.62**	**87.60**	**115.4733**	**25.136**	**74.864**
**R3**	**76.00**	**45.17**	**39.71**	**53.6267**	**11.673**	**88.327**

C- K-562

code	RLU			Mean	Activity %	Inhibition %
**K-562**	**229.96**	**243.54**	**240.84**	**238.1133**	**100.000**	**0.000**
**Nexavar**	**10.2**	**8.66**	**11.61**	**10.1567**	**4.265**	**95.735**
**N1**	**64.7**	**90.84**	**101.5**	**85.6800**	**35.983**	**64.017**
**N3**	**65.77**	**116.8**	**112.08**	**98.2167**	**41.248**	**58.752**
**N5**	**145.46**	**113.6**	**147.74**	**135.6000**	**56.948**	**43.052**
**R2**	**73.30**	**96.08**	**98.46**	**89.2800**	**37.495**	**62.505**
**R3**	**49.92**	**58.96**	**43.50**	**50.7933**	**21.332**	**78.668**

Enzyme assay (EGFR kinase):

A- MCF7

code	RLU			Mean	Activity %	Inhibition %
MCF7	544.40	593.11	557.05	564.8533	100.00	0.00
Erlotinib	365.7	381.68	376.6	374.66	66.329	33.671
N1	116.0	98.69	116.82	110.5033	19.563	80.437
N3	176.4	175.6	160.19	170.73	30.226	69.774
N5	141.6	138.99	148.0	142.8633	25.292	74.708
R2	113.02	118.2	111.8	114.34	20.242	79.758
R3	110.4	101.20	105.9	105.8333	18.736	81.264

B- HepG2

code	RLU			Mean	Activity %	Inhibition %
HepG2	631.1	681.63	677.7	663.477	100.000	0.000
Erlotinib	390.4	356.9	388.7	378.667	57.073	42.927
N1	107.5	113.46	103.43	108.130	16.297	83.703
N3	119.22	122.2	125.37	122.263	18.428	81.572
N5	379.52	332.88	327.84	346.747	52.262	47.738
R2	101.9	128.50	103.8	111.400	16.790	83.210
R3	105.9	104.3	113.8	108.000	16.278	83.722

C- K-562

code	RLU			Mean	Activity %	Inhibition %
K-562	469.0	471.24	461.5	467.247	100.000	0.000
Erlotinib	264.29	285.57	259.81	269.890	57.762	42.238
N1	94.31	98.63	114.8	102.580	21.954	78.046
N3	88.09	70.62	74.51	77.740	16.638	83.362
N5	247.41	208.5	209.3	221.737	47.456	52.544
R2	132.28	172.73	170.21	158.407	33.902	66.098
R3	79.59	85.97	77.01	80.857	17.305	82.695

Gene Expression of (Caspase-3 and Caspase -9) gene in HepG2 cell line.

Caspase -3 level

Sample code	Caspase-3 (ng/ml) ±SE			Notes
HepG2	0.46	0.013		
N1	0.98	0.04		
N2	1.77	0.08		
N3	0.68	0.013		
N4	0.77	0.069		
N5	1.08	0.028		
R1	1.25	0.090		
R2	1.52	0.017		
R3	1.10	0.046		
R4	1.82	0.047		
R5	1.54	0.11		

Caspase -9 level

Sample code	Caspase-9 (ng/L) ±SE			Notes
HepG2	11.01	±5.42		
N1	30.57	3.39		
N2	70.03	0.47		
N3	43.52	3.69		
N4	25.20	1.27		
N5	7.31	0.93		
R1	26.98	1.30		
R2	74.01	3.92		
R3	83.31	1.28		
R4	50.58	4.19		
R5	48.44	1.24		

Gene Expression of (IL-6 and TNF-alpha gene) in HepG2 cell line.

IL-6 level

Sample code	IL-6 (ng/L) ±SE			Notes
HepG2	44.10	2.57		
8a	26.0	0.97		
8b	11.87	0.80		
8c	16.64	0.83		
8d	25.25	0.16		
8e	28.78	1.23		
9a	14.73	1.06		
9b	19.54	1.62		
9c	24.59	0.95		
9d	10.13	2.40		
9e	11.93	0.25		

Sample code	TNF-alpha (ng/L) ±SE			Notes
HepG2	43.48	0.67		
8a	23.6	1.35		
8b	16.86	2.39		
8c	10.2	1.53		
8d	34.27	1.94		
8e	27.34	1.95		
9a	9.13	0.37		
9b	14.37	0.45		
9c	23.47	0.56		
9d	16.01	1.33		
9e	32.07	2.32		

Gene Expression of (Bax and P-cl2 gene) in HepG2 cell line.

Experiment: **Relative Quantification of Gene expression (Fold change)**

Institute / Researcher: **Dr. Alaa Elwan**

Samples number: **2 group**

Experiment design: **Gene Expression of (Bax and Pcl2 gene) in HepG2 cell line.**

Laboratory comments:

Extraction of the total RNA is according to Qiagen Kit

Purification of total RNA from testis tissue using the RNeasy® Mini Kit

- At the end of purification procedure the extracted RNA will be evaluated to insure that the extracted RNA well purified and free of contamination, this will be achieved by measuring the extract by U.V spectrophotometer at wavelength 260/280 nm (**we use Denovix Spectrophotometer AGBL USA**).

Concentration and purity of Extracted RNA:

Sample	Conc. ng/µl			A 260			A mean (260/280)	Notes
THLE-2	68.08	68.48	66.904	1.702	1.712	1.6726	1.72	
HepG2	69.32	69.80	69.92	1.733	1.745	1.748	1.67	
N1	67.56	67.28	66.92	1.689	1.682	1.673	1.60	
R2	71.48	71.00	70.48	1.787	1.775	1.762	1.63	

Converting extracted RNA into Double Stranded DNA (ds DNA) for PCR reaction

The conversion achieved by reverse transcriptase according to Qiagen QuantiTect RT kit.

For quantitative, real-time PCR:

Optimized kit for quantitative, real-time PCR, which includes Taq polymerase; quantitative, real-time PCR buffer; primers; SYBR® Green I dye; and nucleotides

Primer sequence:

Gene	Sequence	Tm	Product size (bp)	Accession Number
**BAX**	**F:** 5′-GATTACAGACCCCAGGCAGG-3′<br>**R:** 5′-TGGCTCAAGTAGGACGGGTA-3′	54	130	**(NM_001291428)**
**Bcl-2**	**F:** 5′-GCAATGGGCACGAGTTTGTT-3′<br>**R:** 5′- AGTGTGTTCACCAGGCCAAA-3′	60	170	**(NM_000633)**
**GAPDH**	**F:** 5′-CCATCAACGACCCCTTCATT-3′<br>**R:** 5′-CACGACATACTCAGCACCAGC-3′	**58**	193	**(NM_001256799)**

- qPCR data analyzed by double delta Ct analysis

The double delta Ct analysis assumes that:

- There is equal primer efficiency between primer sets (i.e. within 5%);
- There is near 100% amplification efficacy of the reference and the target genes;
- The internal control genes are constantly expressed and aren't affected by the treatment.

The method generally caters to experiments with a large number of DNA samples and a low number of genes to be tested.

1. Take the average of the Ct values for the housekeeping gene and the gene being tested in the experimental and control conditions, returning 4 values. The 4 values are Gene being Tested Experimental (TE), Gene being Tested Control (TC), Housekeeping Gene Experimental (HE), and Housekeeping Gene Control (HC).

2. Calculate the differences between experimental values (TE – HE) and the control values (TC – HC). These are your \DeltaΔCt values for the experimental (\DeltaΔCTE) and control (\DeltaΔCTC) conditions, respectively.

3. Then, calculate the difference between the \DeltaΔCT values for the experimental and the control conditions (\DeltaΔCTE – \DeltaΔCTC) to arrive at the double delta Ct value (ddCt).

4. Since all calculations are in logarithm base 2, every time there is twice as much DNA, your Ct values decrease by 1 and will not halve. You need to calculate the value of 2^{-2\Delta\Delta C_{t}}−2ΔΔ*Ct* to get the expression fold change. **(Kenneth J. Livak and Thomas D. Schmittgen 2001)**.

PCR condition:

Start Activation Temperature: 95 C for 3 min.

Denaturation Temperature: 95 C for 30 sec.

Annealing Temperature: (54 C for 40 sec.)

Extension Temperature: 72 C for 45 Sec.

- There are 40 cycles for the complete run.
- Reaction Volume **10 μL**: Master mix 10 μL, 0.5 μL for each Primer (F-R), 2 μL of DNA template and 7 μL DD – RNAse – DNAse- free Water

Results:

Group type	BAX: Ct			Mean Bax-Ct	GAPDH (H.K.G): Ct			Mean HKG-Ct	ΔCt	ΔΔCt	$2^{-\Delta\Delta Ct}$ Fold Change
THLE-2	31.11	35.04	34.65	**33.60**	19.48	18.02	19.33	**18.94**	**14.66**	**0.1044**	**0.9302**
THLE-2	34.24	34.17	35.24	**34.55**	18.28	20.94	19.79	**19.67**	**14.88**	**-0.1189**	**1.0859**
THLE-2	35.34	34.91	34.44	**34.90**	22.13	20.14	18.18	**20.15**	**14.75**	**0.0144**	**0.9900**
HepG2	36.93	37.18	36.01	**36.71**	21.21	20.24	20.88	**20.78**	**15.93**	**1.1689**	**0.4448**
N1	33.94	30.72	32.24	**32.30**	17.87	19.24	19.73	**18.95**	**13.35**	**-1.4078**	**2.6533**
R2	35.02	33.24	34.05	**34.10**	19.02	20.12	21.13	**20.09**	**14.01**	**-0.7478**	**1.6792**

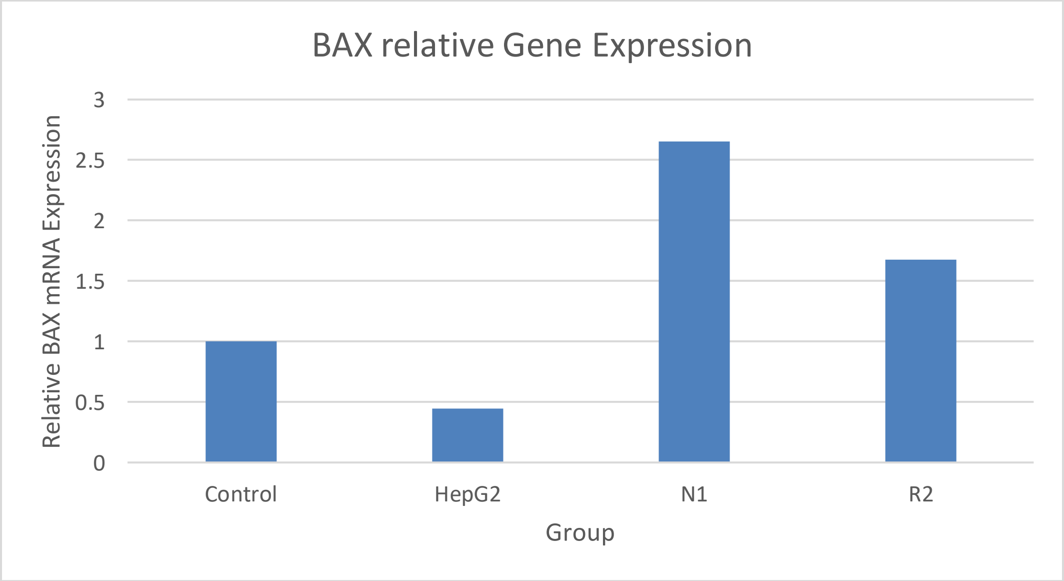

Group type	BCL2: Ct			Mean Bcl2-Ct	GAPDH (H.K.G): Ct			Mean HKG-Ct	ΔCt	ΔΔCt	$2^{-\Delta\Delta Ct}$ Fold Change
THLE-2	36.71	37.47	36.85	**37.01**	19.48	18.02	19.33	**18.94**	**18.07**	**-0.0167**	**1.0116**
THLE-2	37.28	37.73	38.12	**37.71**	18.28	20.94	19.79	**19.67**	**18.04**	**0.0100**	**0.9931**
THLE-2	38.88	38.59	37.11	**38.19**	22.13	20.14	18.18	**20.15**	**18.04**	**0.0067**	**0.9954**
HepG2	37.42	37.98	35.66	37.02	21.21	20.24	20.88	20.78	**16.24**	**-1.8067**	**3.4983**
N1	36.04	37.02	37.01	36.69	17.87	19.24	19.73	18.95	**17.74**	**-0.3067**	**1.2368**
R2	37.92	38.84	37.95	38.24	19.02	20.12	21.13	20.09	**18.15**	**0.0967**	**0.9352**

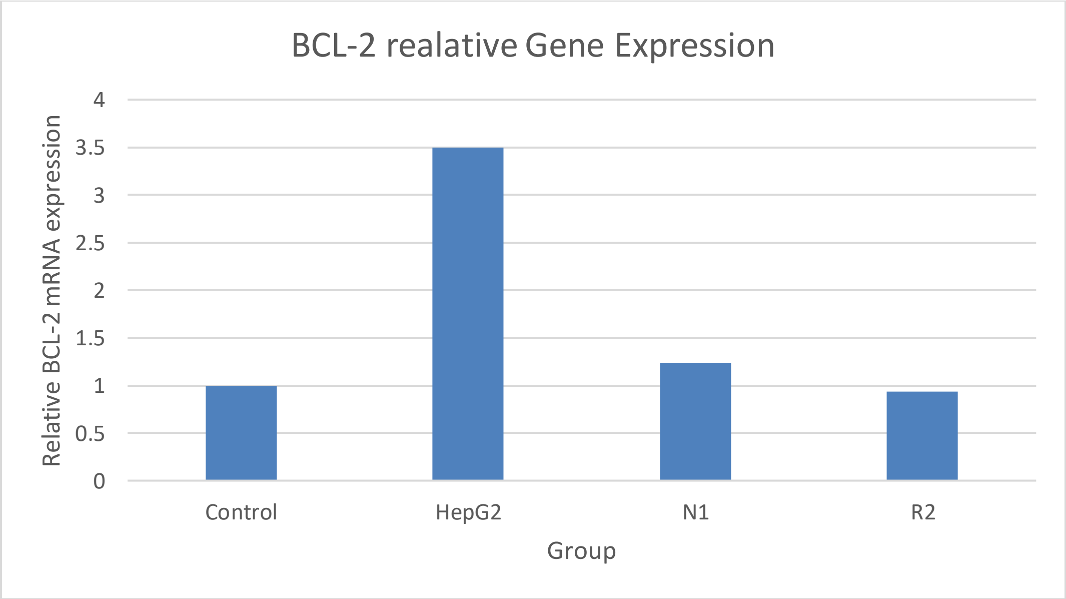

Cell cycle analysis

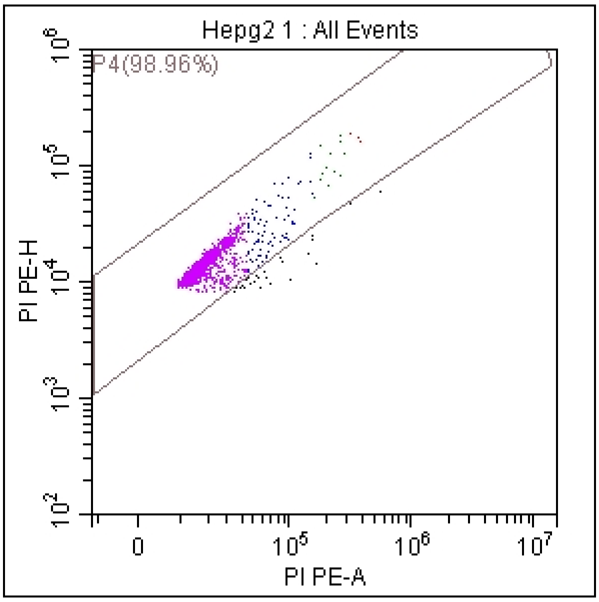

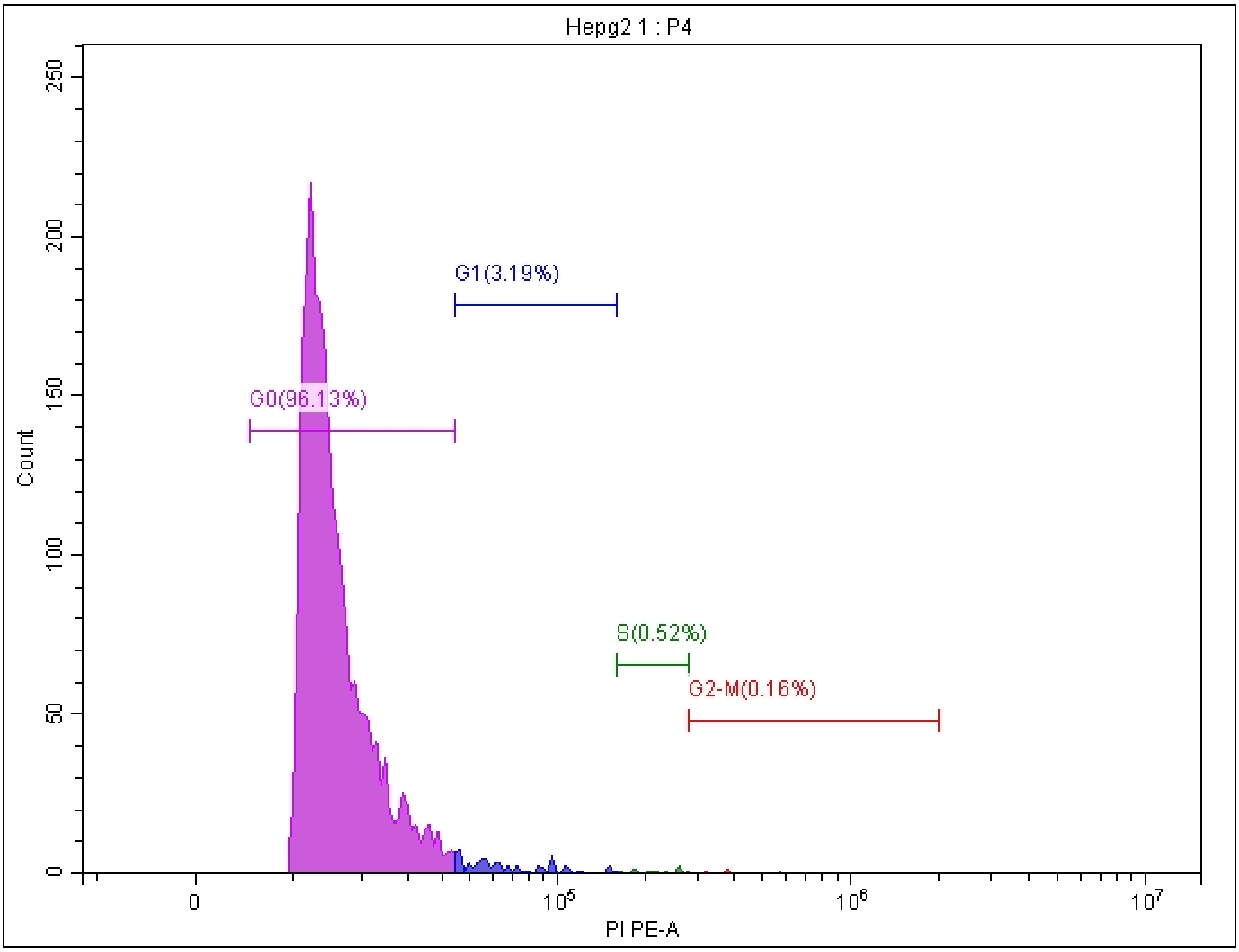

Tube Name: Hepg2 1
Sample ID:

Population	Events	% Total	% Parent
G0	2382	95.13%	96.13%
S	13	0.52%	0.52%
G2-M	4	0.16%	0.16%
G1	79	3.15%	3.19%

Cell cycle analysis

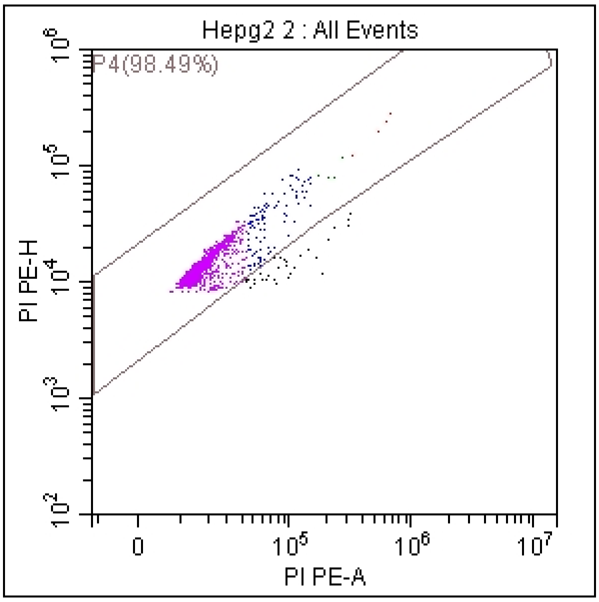

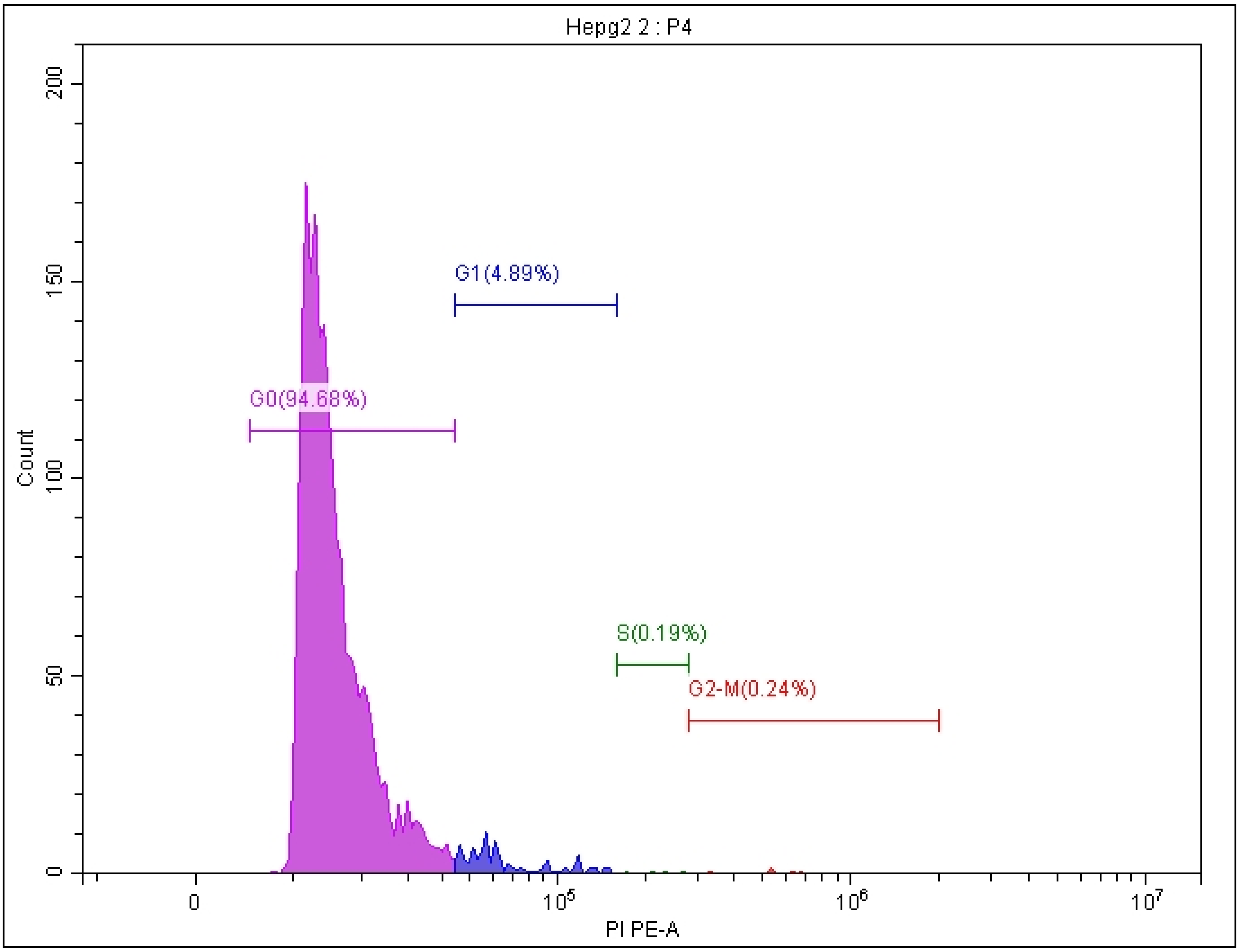

Tube Name: Hepg2 2
Sample ID:

Population	Events	% Total	% Parent
G0	1976	93.25%	94.68%
S	4	0.19%	0.19%
G2-M	5	0.24%	0.24%
G1	102	4.81%	4.89%

5- **Experimental of *in silico* studies**

4.3. Docking studies

The docking studies were performed utilizing MOE.14 software to explore the binding mode of the synthesized compounds towards VEGFR-2. The 3D crystal structures of the target macromolecules VEGFR-2 were downloaded from the protein databank, http://www.pdb.org (PDB ID; 4ASD). Sorafenib was used as reference ligand. To prepare the target protein, water molecules were removed, and the valances of atoms were corrected through protonation of the whole molecule. Then energy minimization was carried out by applying CHARMM and MMFF94 force fields. After that, the active binding site was defined and prepared for docking. The validation process was performed by redocking the co-crystallized ligand. The designed compounds together with sorafenib were drawn using ChemBioDraw Ultra 14.0 and saved as MDL-SD format. The sketched compounds were constructed from fragment libraries in MOE program, protonated, followed by energy minimization then prepared for docking. Docking process was carried through Triangle matcher placement inserted in compute window, and the scoring function was London dG. Ten conformers (poses) for each molecule were generated using genetic algorithm searches. The free energies and binding modes of the designed molecules against VEGFR-2 were determined. The most ideal pose was selected according to its binding free energy as well as its binding mode with target molecules.

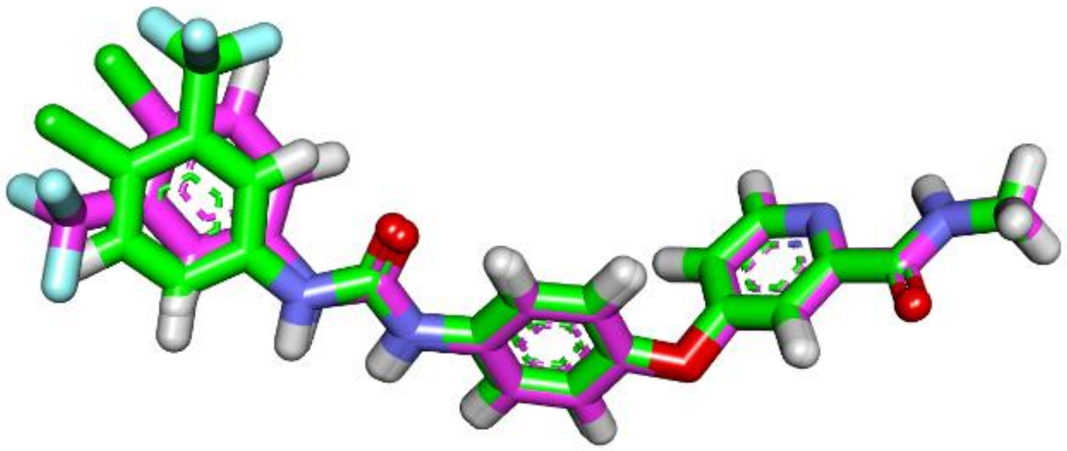

Fig. S1. Alignment of the native and re-docked co-crystallized ligand

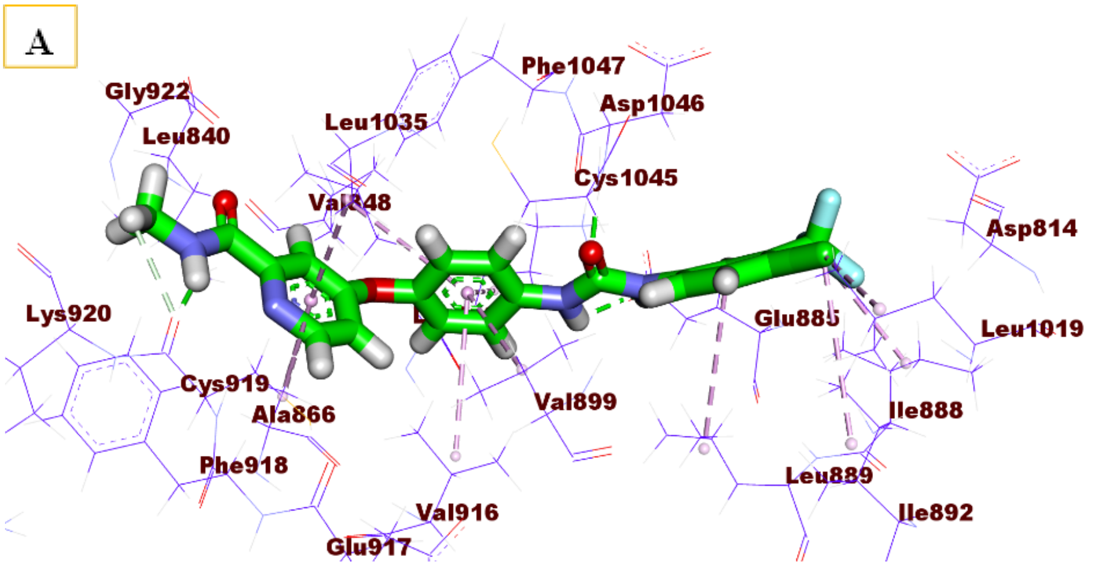

Fig. S2. (A) 3D binding mode of sorafenib into VEGFR-2)

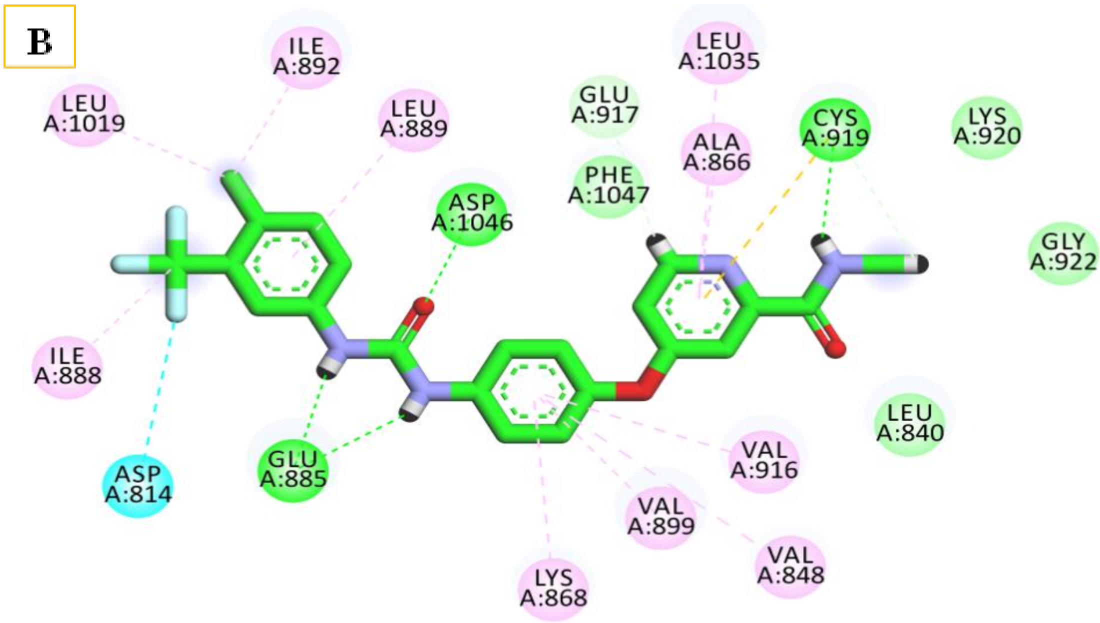

Fig. S2. (B) 2D binding mode of sorafenib into VEGFR-2)
